# Identification of spontaneous mutation for broad-spectrum brown planthopper resistance in a large, long-term fast neutron mutagenized rice population

**DOI:** 10.1186/s12284-019-0274-1

**Published:** 2019-03-19

**Authors:** Wintai Kamolsukyeunyong, Siriphat Ruengphayak, Pantharika Chumwong, Lucia Kusumawati, Ekawat Chaichoompu, Watchareewan Jamboonsri, Chatree Saensuk, Kunyakarn Phoonsiri, Theerayut Toojinda, Apichart Vanavichit

**Affiliations:** 1 Rice Gene Discovery and Utilization Laboratory, Innovative Plant Biotechnology and Precision Agriculture Research Team, National Center for Genetic Engineering and Biotechnology (BIOTEC), National Science and Technology Development Agency (NSTDA), Khlong Luang, Pathum Thani Thailand; 2 Rice Science Center, Kasetsart University Kamphaeng Saen Campus, Nakhon Pathom, Thailand; 3 Interdisciplinary Graduate Program in Genetic Engineering and Bioinformatics, Kasetsart University, Chatuchak, Bangkok Thailand; 4 Integrative Crop Biotechnology and Management Research Group, National Center for Genetic Engineering and Biotechnology (BIOTEC), National Science and Technology Development Agency (NSTDA), Khlong Luang, Pathum Thani Thailand; 5 Agronomy Department, Faculty of Agriculture at Kamphaeng Saen, Kasetsart University, Kamphaeng Saen, Nakhon Pathom Thailand

**Keywords:** Brown planthopper, BPH resistance gene, Double digest restriction site-associated DNA sequencing, QTL-seq, Single-nucleotide polymorphism, Haplotype pattern, Fast-neutron mutagenesis

## Abstract

**Background:**

The development of rice varieties with broad-spectrum resistance to insect pests is the most promising approach for controlling a fast evolving insect pest such as the brown planthopper (BPH). To cope with rapid evolution, discovering new sources of broad-spectrum resistance genes is the ultimate goal.

**Results:**

We used a forward genetics approach to identify BPH resistance genes in rice (*Oryza sativa* L.) using double digest restriction site-associated DNA sequencing (ddRADseq) for quantitative trait loci (QTL)-seq of the backcross inbred lines (BILs) derived from a cross between the BPH-susceptible cultivar KDML105 and BPH-resistant cultivar Rathu Heenati (RH). Two major genomic regions, located between 5.78–7.78 Mb (QBPH4.1) and 15.22–17.22 Mb (QBPH4.2) on rice chromosome 4, showed association with BPH resistance in both pooled BILs and individual highly resistant and susceptible BILs. The two most significant candidate resistance genes located within the QBPH4.1 and QBPH4.2 windows were lectin receptor kinase 3 (*OsLecRK3*) and sesquiterpene synthase 2 (*OsSTPS2*), respectively. Functional markers identified in these two genes were used for reverse screening 9323 lines of the fast neutron (FN)-mutagenized population developed from the BPH-susceptible, purple-pigmented, *indica* cultivar Jao Hom Nin (JHN). Nineteen FN-mutagenized lines (0.24%) carried mutations in the *OsLecRK3* and/or *OsSTPS2* gene. Among these mutant lines, only one highly resistant line (JHN4) and three moderately resistant lines (JHN09962, JHN12005, and JHN19525) were identified using three active, local BPH populations. The 19 mutant lines together with three randomly selected mutant lines, which did not harbor mutations in the two target genes, were screened further for mutations in six known BPH resistance genes including *BPH9*, *BPH14*, *BPH18*, *BPH26*, *BPH29*, and *BPH32*. Multiple single nucleotide polymorphisms (SNPs) and insertion-deletion (Indel) mutations were identified, which formed gene-specific haplotype patterns (HPs) essential for broad-spectrum resistance to BPH in both BILs and JHN mutant populations.

**Conclusion:**

On the one hand, HPs of *OsLekRK2–3*, *OsSTPS2*, and *BPH32* determined broad-spectrum resistance to BPH among RH-derived BILs. On the other hand, in the JHN mutant population, *BPH9* together with seven significant genes on chromosome 4 played a crucial role in BPH resistance.

**Electronic supplementary material:**

The online version of this article (10.1186/s12284-019-0274-1) contains supplementary material, which is available to authorized users.

## Background

Rice (*Oryza sativa* L.) is an important cereal crop that feeds almost half of the world’s population (Mohanty 2013), and is mostly grown in Asia (Muthayya et al. 2014). Insect pests, such as brown planthopper (BPH), white-backed hopper, green leafhopper, stem borer, and gall midge, cause severe damage to the rice crop across the world (Ane and Hussain 2016). Among these, BPH (*Nilaparvata lugens* Stål) is one of the most economically damaging insect pests of rice, and causes substantial losses in yield in the major rice growing areas in Asia each year.

During plant development from the seedling to the reproductive stage, BPH sucks the phloem sap, causing whole plant senescence called hopper burn (Lou et al. 2005; Dale 1994). A significant threat to rice production was reported in the early 1970s, when new high yielding varieties and extensive use of fertilizers and pesticides caused the rapid evolution of pesticide-tolerant biotypes of insect pests. These newly introduced rice cultivars, lacking broad-spectrum resistance, exhibit short life spans. Therefore, the best approach to prolong BPH resistance is to develop rice cultivars with durable resistance against multiple BPH biotypes. More than 30 BPH resistance genes or quantitative trait loci (QTL) have been genetically mapped (Ling and Weilin 2016). However, only eight have been cloned, including *BPH14* (Du et al. 2009), *BPH26* (Tamura et al. 2014), *BPH3* (Liu et al. 2014), *BPH29* (Wang et al. 2015), *BPH 9* (Zhao et al. 2016), *BPH18* (Ji et al. 2016), *BPH32* (Ren et al. 2016), and *BPH31* (Prahalada et al. 2017).

*BPH9* (LOC_Os12g37290) is located on chromosome 12, and is a highly diversified and complex BPH resistance gene, with a complex allelic composition called allelotype (Zhao et al. 2016). Four allelotypes of this locus, including *BPH1/9–1*, *− 2*, *− 7*, and *−* 9, have been identified from eight rice varieties. The first widely used allelotype, *BPH1/9–1*, was identified in the rice cultivar Mudgo (*BPH1*) (Pathak et al. 1969) and three wild rice species, *Oryza officinalis* (*BPH10*) (Thi Lang and Chi Buu 2003), *O. australiensis* (*BPH18*) (Jena et al. 2006), and *O. minuta* (*BPH21*) (Rahman et al. 2009). Two members of the allelotype *BPH 1/9–2* were derived from rice cultivars ASD7 (*BPH2*) (Jena and Kim 2010) and ADR52 (*BPH26*) (Cheng et al. 2013). The last two allelotypes, *BPH1/9–7* and *BPH1/9–9* (known as *BPH7* and *BPH9*, respectively) were identified from the *indica* rice cultivars T12 and Pokkali, respectively (Qiu et al. 2014; Zhao et al. 2016). The coding sequence (CDS) of *BPH9* (Pokkali) is 4042 bp (GenBank accession no. KU216220), and encodes a 1206 amino acid (aa) protein (GenBank accession no. ANC90313). The *BPH9* gene carries 520 polymorphic sites and 21 haplotypes in 117 rice varieties and landraces (Zhao et al. 2016).

Rathu heenati (RH), a Sri Lankan landrace, is one of the most strongest sources of durable resistance against BPH in Southeast Asia. This landrace has been widely used as a BPH resistance donor in breeding programs in the Great Mekong Sub-region, including Thailand, Cambodia, Myanmar, and Laos (Wang et al. 2012). Several BPH resistance loci have been identified in RH, including *BPH3* (Sun et al. 2005) and sesquiterpene synthase II (*OsSTPS2*) (Kamolsukyunyong et al. 2013) on chromosome 4, and *BPH32* (Jairin et al. 2007) on chromosome 6. The *BPH3* locus has been cloned from RH, and comprises the lectin receptor kinase (*OsLecRK1–3*) gene cluster (Liu et al. 2014). The CDSs of *OsLecRK1*, *2*, and *3*genes are 2442, 2436, and 2436 bp, in length, respectively (GenBank accession nos. KF748957, KF748965, and KF748973, respectively). Sequence comparison between rice varieties showing differences in BPH resistance identified 17, 7, and 31 polymorphic sites in *OsLecRK1*, *OsLecRK2*, and *OsLecRK3*, respectively (Liu et al. 2014). The CDS of the *OsSTPS2* gene cloned from RH is 1518 bp in size (GenBank accession no. KC511027), and contains a 21-bp in-frame insertion in exon 5, adding 7-aa to the encoded protein, compared with the protein sequence in the BPH susceptible variety KDML105 (Kamolsukyunyong et al. 2013). The *BPH32* gene contained 20 single nucleotide polymorphisms (SNPs) and several insertion-deletion (Indel) mutations in the 585-bp CDS (Ren et al. 2016).

Rice varieties with durable resistance to BPH are key to sustainable rice production in Asia because of rapidly evolving BPH biotypes and high input farming practices. Natural variation in BPH resistance is limited, as rice is an obligate self-pollinating crop. Chemical and physical mutagenesis has been used to induce mutations and create novel sources of resistance in various genotypes, which are then used as germplasm for the development of new resistant varieties. In rice, induced mutations have been reported using gamma irradiation (Till et al. 2007; Wu et al. 2005), ethyl methanesulfonate (EMS) (Wu et al. 2005), fast neutron (FN) (Wu et al. 2005; Ruengphayak et al. 2015), and ion beam (Yamaguchi et al. 2009). The effects of FN mutagenesis on structural changes in the genome have been reported in soybean (Bolon et al. 2011, 2014; Campbell et al. 2016), *Arabidopsis* (Li et al. 2002), and rice (Ruengphayak et al. 2015; Li et al. 2017). Deletions are the main structural rearrangements induced by FN mutagenesis (Bolon et al. 2011, 2014; Li et al. 2002). In soybean, FN treatment has been shown to induce chromosomal rearrangement near the target gene (Campbell et al. 2016). Induction of single nucleotide variation (SNV) by FN has been reported in the *indica* rice cultivar Jao Hom Nin (JHN) (Ruengphayak et al. 2015) and japonica rice cultivar Kitaake (Li et al. 2017). In Kitaake, SNV is the most abundant mutation, accounting for 48% of the total number of mutations, and 58% of the SNVs are located within rice genes (Li et al. 2017). Structural variation in CDSs may create useful mutations; for example, tandem duplication in the *waxy* gene of rice (Wanchana et al. 2003), structural rearrangement in *NAP1* gene in the *gnarled* trichrome mutant of soybean (Campbell et al. 2016), and haplotype change in *OsFRO1* in a rice mutant tolerant to iron (Fe) toxicity (Ruengphayak et al. 2015).

In the current study, we combined QTL-seq (Takagi et al. 2013) and double digest restriction site-associated DNA sequencing (ddRADseq) (Peterson et al. 2012) to scan the entire rice genome for the identification of genes responsible for BPH resistance. The derived SNPs were used for reverse screening of a large FN-derived mutant population. We demonstrated that haplotype patterns (HPs) of *OsLecRK2*, *OsLecRK3*, *OsSTPS2*, and *BPH32* contributed significantly to broad-spectrum BPH resistance in RH-derived backcross inbred lines (BILs). Additionally, HPs of *BPH9* together with HPs of seven significant genes on chromosome 4 played a crucial role in broad-spectrum BPH resistance in the FN-derived mutant population.

## Results

### Distribution of BPH-damage AUC in BILs

**Fig. 1:**
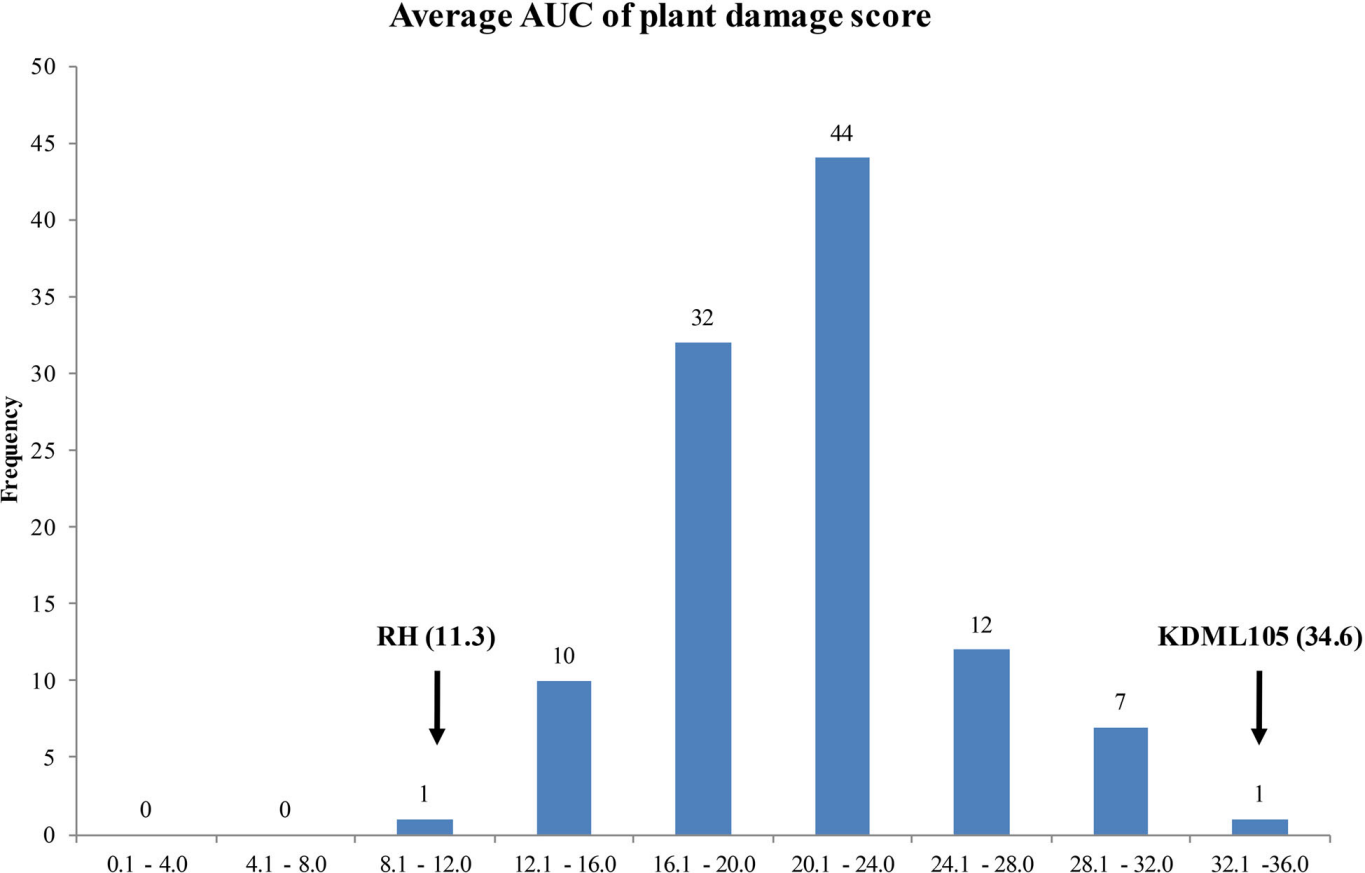
Frequency distribution of average AUC of damage scores on BILs from 6 BPH populations

Based on the average area under the curve (AUC) of BPH damage scores from six BPH populations (Additional file 1: Table S1), the distribution of BILs, KDML105, and RH are shownin Fig.1. According to the AUC scores, KDML105 (AUC = 34.6) and RH (AUC = 11.3) parental lines were marked as the most susceptible and resistant genotypes, respectively, whereas the AUC scores of their progeny ranged from 31.8 to 13.9. On the extreme tails, 16 and 19 BILs selected for susceptible and resistance pools have the average AUCs ranged from 23.4–31.8 and 13.9–18.4, respectively (Additional file 2: Figure S1). Also, within each pool, 4 susceptible and 5 resistance BILs selected as extreme individuals have the average AUCs ranged from 28.0–34.3 and 13.9–16.0, respectively (Additional file 2: Figure S2). These selected BILs were used in the next part for QTL-seq/ddRAD analysis.

### Genome-wide SNP index analysis and SNP mining in BILs

**Fig. 2:**
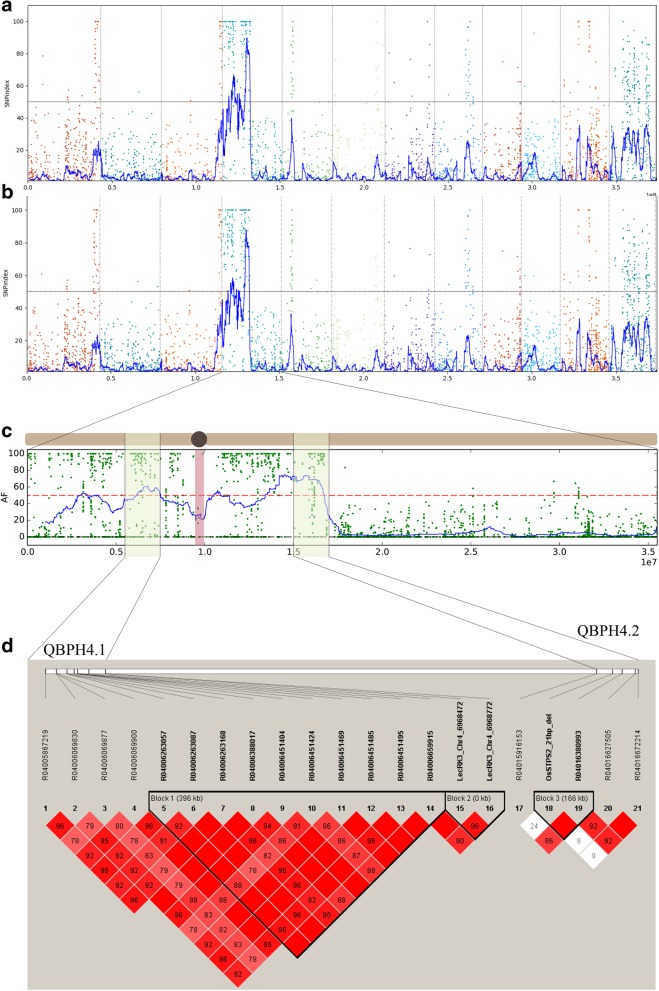
The genome-wide scanning of average ΔSNP-indexes graphs plotted along the rice genome. **a** The individual extreme BILs, **b** the pooled extreme BILs. **c** Candidate QTL locations were identified on rice chromosome 4 with statistical confidence intervals under the null hypothesis of no QTL (*P* < 0.05). **d** Linkage disequilibrium (LD) plot for SNPs in the QBPH4.1 and QBPH4.2 regions. The D’ values (normalized linkage disequilibrium scores) for SNPs covered the LD blocks were shown. The SNPs were genotyped in the BIL population by KASP genotyping

A total of 33.75 million paired-end reads were generated from the sequencing of 11 ddRADseq libraries (NCBI accession nos. SRR7851264 to 69, SRR7851271, SRR7851273 to 76), and SNPs in each library were called for QTL-seq analysis (Additional file 1: Tables S2, S3). Using a 2 Mb-sliding window for genome-wide scanning, 37,212 windows were allocated to the reference genome. Based on the maximum average ΔSNP-indexes (max ΔSNP) of individuals and pools, two QTLs for BPH resistance were mapped on genomic windows between 5.78–7.78 Mb (QBPH4.1) and 15.22–17.22 Mb (QBPH4.2), on the short and long arms of chromosome 4, respectively (Fig. 2a, b). Linkage disequilibrium analysis of polymorphic SNPs in QBPH4.1 and QBPH4.2 in the BIL population revealed three haplotype blocks (Fig. 2d). The first block covered a 396-kb genomic region containing F-box118 (LOC_Os04g11450), F-box119 (LOC_Os04g11660), resistance protein LR10 (LOC_Os04g11780), and an unknown protein (LOC_Os04g12110). The second haplotype block contained *OsLecRK3* (LOC_Os04g12580), whereas the third block contained *OsSTPS2* (LOC_Os04g27430).

A total of 107 and 94 SNPs were identified in QBPH4.1 and QBPH4.2 genomic windows, respectively. In the QBPH4.1 window, 31 SNPs were identified in 11 genes, resulting in 22 non-synonymous mutations and one premature stop codon (Table 1). In the F-box119 protein, two amino acids change from aspartic acid (D) to glutamic acid (E) and lysine (K) to asparagines (N) were found at position 253 and 279 respectively. In the resistance protein LR10, six amino acid changes were identified at positions 371 (isoleucine [I] to threonine [T]), 377 (glutamic acid [E] to aspartic acid [D]), 378 (leucine [L] to methionine [M]), 393 (valine [V] to isoleucine [I]), 398 (lysine [K] to arginine [R]), and 401 (aspartic acid [D[to glutamic acid [E]) which were capable of changing the function of LR10 protein. Twenty-two gene-specific SNPs from eight candidate genes in the QBPH4.1 window were selected for marker development. In the QBPH4.2 window, 14 SNPs and one indel were identified in 11 candidate genes including one non-synonymous SNP (serine [S] to arginine [R]) located at amino acid position 387 in the protein encoded by the terpene synthase gene (LOC_Os04g27720) (Table 2). In total, five SNPs and one indel marker were developed from the QBPH4.2 region. Additionally, the region on chromosome 6 in RH spanning 0.03–2.03 Mb, which harbors the previously cloned BPH resistance-conferring *BPH32* gene, (Ren et al. 2016), showed the max ΔSNP. This window contained nine non-synonymous mutations in eight annotated genes. Unfortunately, no SNP was identified in the *BPH32* gene using ddRADseq data (Additional file 1: Table S4). Therefore, the SNP marker identified previously in *BPH32* by Ren et al. (2016) was used as a marker in this study. All of the SNPs identified in QBPH4.1, QBPH4.2, and *BPH32* were used for QTL detection in the BIL population using single marker analysis.

**Table 1:** List of SNP identified on the QBPH4.1 sliding window (5.78–7.78 Mb) on chromosome 4. SI = SNP index; QS = QBPHS; QR = QBPHR; IndS = BPHS; IndR = BPHR; AAC = amino acid change; (−) = synonymous variant; (+) = missense variant or stop codon

Position	LOC identifier	gene product name	KASP-SNP marker	SNP S/R	SI in QS	SI in QR	SI in IndS	SI in IndR	SNP pos / CDS length	SNP effect	AAC	AAC position/protein length
5,806,676	LOC_Os04g10680	zinc finger, C3HC4 type domain containing protein		T/C	0.0	100.0	0.0	100.0	2071/3059	–		
5,867,185	LOC_Os04g10800	inorganic phosphate transporter		A/G	NA	NA	0.0	100.0	534/1527	–		
5,867,219	LOC_Os04g10800	inorganic phosphate transporter	R04005867219	G/C	0.0	85.0	0.0	100.0	568/1527	+	A > P	190/508
5,867,260	LOC_Os04g10800	inorganic phosphate transporter		T/C	0.9	82.9	0.0	100.0	609/1527	–		
5,867,320	LOC_Os04g10800	inorganic phosphate transporter		T/G	0.0	80.0	0.0	100.0	669/1527	–		
6,069,830	LOC_Os04g11165	gamma-thionin family domain containing protein	R04006069830	G/T	NA	NA	100.0	0.0	201/216	+	stop codon	67/71
6,069,877	LOC_Os04g11165	gamma-thionin family domain containing protein	R04006069877	A/G	0.0	81.0	0.0	81.0	154/216	+	C > R	52/71
6,069,900	LOC_Os04g11165	gamma-thionin family domain containing protein	R04006069900	A/G	0.0	81.0	0.0	81.0	131/216	+	I > T	44/71
6,263,052	LOC_Os04g11450	OsFBX118 - F-box domain containing protein		A/G	8.2	91.7	0.0	88.9	1011/1480	–		
6,263,057	LOC_Os04g11450	OsFBX118 - F-box domain containing protein	R04006263057	G/C	91.8	4.0	100.0	0.0	922/1203	+	Q > E	308/400
6,263,087	LOC_Os04g11450	OsFBX118 - F-box domain containing protein	R04006263087	A/T	8.2	92.0	0.0	90.0	892/1203	+	Y > N	298/400
6,263,168	LOC_Os04g11450	OsFBX118 - F-box domain containing protein	R04006263168	C/G	38.8	96.9	0.0	96.8	811/1203	+	V > L	271/400
6,263,172	LOC_Os04g11450	OsFBX118 - F-box domain containing protein		T/A	36.7	96.5	0.0	96.0	891/1480	–		
6,388,017	LOC_Os04g11660	OsFBX119 - F-box domain containing protein	R04006388017	C/A	16.7	97.7	16.7	97.7	837/1137	+	K > N	279/378
6,388,020	LOC_Os04g11660	OsFBX119 - F-box domain containing protein		A/G	16.7	97.7	16.7	97.7	916/1766	–		
6,388,038	LOC_Os04g11660	OsFBX119 - F-box domain containing protein		G/T	13.5	100.0	0.0	100.0	898/1766	–		
6,388,095	LOC_Os04g11660	OsFBX119 - F-box domain containing protein	R04006388095	G/C	10.8	100.0	0.0	100.0	759/1137	+	D > E	253/378
6,451,402	LOC_Os04g11780	resistance protein LR10		C/A	0.0	100.0	0.0	100.0	1110/1998	–		
6,451,404	LOC_Os04g11780	resistance protein LR10	R04006451404	T/C	0.0	100.0	0.0	100.0	1112/1998	+	I > T	371/665
6,451,423	LOC_Os04g11780	resistance protein LR10		A/T	0.0	100.0	0.0	100.0	1131/1998	+	E > D	377/665
6,451,424	LOC_Os04g11780	resistance protein LR10	R04006451424	C/A	0.0	100.0	0.0	100.0	1132/1998	+	L > M	378/665
6,451,469	LOC_Os04g11780	resistance protein LR10	R04006451469	G/A	2.9	100.0	2.9	100.0	1177/1998	+	V > I	393/665
6,451,485	LOC_Os04g11780	resistance protein LR10	R04006451485	A/G	2.9	100.0	2.9	100.0	1193/1998	+	K > R	398/665
6,451,495	LOC_Os04g11780	resistance protein LR10	R04006451495	T/G	2.9	100.0	2.9	100.0	1203/1998	+	D > E	401/665
6,455,216	LOC_Os04g11790	OsFBX120 - F-box domain containing protein		T/C	25.0	98.3	0.0	95.8	827/1461	–		
6,455,225	LOC_Os04g11790	OsFBX120 - F-box domain containing protein		C/T	75.0	1.6	100.0	4.2	818/1461	–		
6,593,508	LOC_Os04g12010	glycosyltransferase		G/T	24.3	96.6	0.0	94.5	1455/1774	–		
6,593,541	LOC_Os04g12010	glycosyltransferase		G/A	22.2	96.7	0.0	94.8	1422/1774	–		
6,659,796	LOC_Os04g12110	expressed protein		C/A	NA	NA	100.0	0.0	1818/1839	–		
6,659,820	LOC_Os04g12110	expressed protein		G/A	75.9	0.0	75.9	0.0	1794/1839	–		
6,659,826	LOC_Os04g12110	expressed protein		A/G	100.0	11.1	100.0	11.1	1788/1839	–		
6,659,838	LOC_Os04g12110	expressed protein		A/G	100.0	11.1	100.0	11.1	1776/1839	–		
6,659,844	LOC_Os04g12110	expressed protein	R04006659844	C/A	75.9	0.0	75.9	0.0	1770/1839	+	R > S	590/612
6,659,845	LOC_Os04g12110	expressed protein	R04006659845	C/G	75.9	0.0	75.9	0.0	1769/1839	+	R > T	590/612
6,659,847	LOC_Os04g12110	expressed protein		G/T	75.5	0.0	75.5	0.0	1767/1839	–		
6,659,872	LOC_Os04g12110	expressed protein	R04006659872	C/T	88.6	5.3	100.0	7.4	1742/1839	+	C > Y	581/612
6,659,873	LOC_Os04g12110	expressed protein	R04006659873	A/T	88.6	5.3	100.0	7.4	1741/1839	+	C > S	581/612
6,659,874	LOC_Os04g12110	expressed protein		A/G	88.6	5.3	100.0	7.4	1740/1839	–		
6,659,877	LOC_Os04g12110	expressed protein		A/C	88.6	5.3	100.0	7.4	1737/1839	–		
6,659,884	LOC_Os04g12110	expressed protein	R04006659884	G/C	89.1	5.3	100.0	7.4	1730/1839	+	S > C	577/612
6,659,915	LOC_Os04g12110	expressed protein	R04006659915	T/C	88.9	5.4	100.0	7.4	1699/1839	+	I > V	567/612
6,925,203	LOC_Os04g12530	amino acid transporter family protein	R04006925203	T/G	0.0	80.0	0.0	80.0	404/612	+	D > A	135/203
6,925,208	LOC_Os04g12530	amino acid transporter family protein		C/G	NA	NA	0.0	75.0	399/612	–		
6,925,211	LOC_Os04g12530	amino acid transporter family protein		C/G	NA	NA	0.0	71.4	396/612	–		
6,968,050	LOC_Os04g12580	receptor-like protein kinase (OsLecRK3)	OsLecRK3-SNP	A/G	NA	NA	0.0	100.0	990/2346	–		
6,977,171	LOC_Os04g12600	receptor-like protein kinase		G/A	96.6	0.0	96.6	0.0	2079/2415	–		
6,978,761	LOC_Os04g12600	receptor-like protein kinase		C/T	100.0	0.0	100.0	0.0	489/2415	–

**Table 2:** List of SNP identified on the QBPH4.2 sliding window (15.22–17.22 Mb) on chromosome 4. SI = SNP index; QS = QBPHS; QR = QBPHR; IndS = BPHS; IndR = BPHR; AAC = amino acid change; (−) = synonymous variant; (+) = missense variant or stop codon

Position	LOC identifier	gene product name	KASP-SNP marker	SNP S/R	SI in QS	SI in QR	SI in IndS	SI in IndR	SNP pos / CDS length	SNP effect	AAC	AAC position/protein length
15,893,411	LOC_Os04g26870	oxidoreductase		A/G	37.8	100.0	0.0	100.0	906/1053	–		
15,893,519	LOC_Os04g26870	oxidoreductase		C/T	NA	NA	0.0	71.4	1014/1053	–		
15,916,097	LOC_Os04g26910	oxidoreductase		T/C	40.8	97.3	0.0	100.0	255/1056	–		
15,916,138	LOC_Os04g26910	oxidoreductase	R04015916138	A/C	42.6	97.1	0.0	100.0	410/1470	+	E > A	99/351
15,916,153	LOC_Os04g26910	oxidoreductase	R04015916153	A/G	46.2	1.7	100.0	0.0	425/1470	+	D > G	104/351
16,044,907	LOC_Os04g27096	expressed protein		G/A	0.0	95.2	0.0	94.1	418/687	–		
16,214,690	LOC_Os04g27430	sesquiterpene synthase2	OsSTPS2_21bp_del	−/TTTATGCCTCTGGTGTGACCA	0.0	100.0	0.0	100.0	477/1416	+	7-aa-ins	313/472
16,380,993	LOC_Os04g27720	terpene synthase	R04016380993	A/C	100.0	1.7	100.0	1.7	1161/1518	+	S > R	387/505
16,582,690	LOC_Os04g28090	MYB family transcription factor		C/T	0.0	100.0	0.0	100.0	621/2919	–		
16,586,281	LOC_Os04g28090	MYB family transcription factor		G/A	100.0	7.7	100.0	7.7	2337/2919	–		
16,627,505	LOC_Os04g28150	expressed protein	R04016627505	C/G	0.0	100.0	0.0	100.0	795/867	+	D > E	265/288
16,632,510	LOC_Os04g28160	response regulator receiver domain containing protein		T/C	0.0	100.0	0.0	100.0	705/1143	–		
16,672,214	LOC_Os04g28210	verticillium wilt disease resistance protein	R04016672214	T/G	21.9	96.2	0.0	88.9	295/3775	+	D > A	85/1034
16,713,777	LOC_Os04g28270	expressed protein		C/T	1.2	97.2	0.0	98.8	285/438	–		
16,732,393	LOC_Os04g28280	BEE 3		T/C	0.0	97.0	0.0	97.0	792/792	–		264/263

### Roles of *OsLecRK3* and *OsSTPS2* in broad-spectrum BPH resistance

To determine candidate genes that were exclusively involved in BPH resistance, single marker QTL analysis was performed using 28 candidate SNPs and BPH damage AUC of the selected BILs infested by five BPH populations. The results revealed a significant association between the BPH resistant QTL and *OsLecRK3*SNP in all five BPH populations with phenotypic variance ranging from 16% to 44% (Table 3). The second most important marker was the 21-bp indel in *OsSTPS2*, which accounted for 8%–30% of the total phenotypic variance in BPH resistance Specific associations were detected between some SNPs and specific BPH populations. The SNP markers in F-Box118 protein, oxidoreductase (LOC_Os04g26910), and *BPH32* showed major associations, while SNP markers specific to the *Verticillium* wilt resistance (VWR) protein (LOC_Os04g28210) showed a minor association, specifically with the Huai Thalaeng (HTL) BPH population. With the Kamphaeng Phet (KPP) BPH population, SNP markers from the LR10 and unknown protein (LOC_Os04g12110) accounted for 4.5–7% of the total phenotypic variation, whereas terpene synthase gene explained approximately 7.6% of the total phenotypic variation in BPH resistance with the Phitsanulok (PSL) BPH population. Therefore, QTL-seq analysis revealed the two most important regions of chromosome 4, which contributed the RH-derived broad-spectrum BPH resistance in BILs.

**Table 3:** Association between markers and BPH resistance in BIL population based on linear regression analysis. Five BPH populations were used, KPP, PSL, UBN, TPY, and HTL. R^2^ indicating the proportion of the phenotypic variation contributed by each marker

BPH	Sliding window	Marker	LOC no.	Chr	P-value	R^2^	KDML105 allele		RH allele	
							count	AUC means	count	AUC means
HTL	QBPH4.1	OsLecRK3_SNP	LOC_Os04g12580	4	< 0.001**	29.6	16	29.812	76	20.474
		R04006263057	LOC_Os04g11450	4	0.008**	9.6	18	22.333	64	21.375
		R04006263168	LOC_Os04h11450	4	0.048*	5.4	16	21.5	65	21.462
	QBPH4.2	OsSTPS2_21bp_indel	LOC_Os04g27430	4	< 0.001**	17.2	11	28.909	77	20.87
		R04015916153	LOC_Os04g26910	4	0.005**	9.2	20	21.9	62	21.177
		R04016672214	LOC_Os04g28210	4	0.014*	7.1	18	23.167	68	22.456
	Chr6:0.03–2.03 Mb	BPH32-SNP	LOC_Os06g03240	6	0.001**	12.4	19	25.61	72	20.59
KPP	QBPH4.1	OsLecRK3_SNP	LOC_Os04g12580	4	< 0.001**	54.9	16	23	76	13.447
		R04006451469	LOC_Os04g11780	4	0.047*	4.5	17	14.412	66	14.773
		R04006659915	LOC_Os04g12110	4	0.026*	6.9	10	15.7	72	14.944
	QBPH4.2	OsSTPS2_21bp_indel	LOC_Os04g27430	4	< 0.001**	49.8	11	14.182	77	13.649
	Chr6:0.03–2.03 Mb	Bph32-SNP	LOC_Os06g03240	6	0.793	na	na	na	na	na
PSL	QBPH4.1	OsLecRK3-SNP	LOC_Os04g12580	4	< 0.001**	21.8	16	28.5	76	21.118
	QBPH4.2	OsSTPS2_21bp_indel	LOC_Os04g27430	4	< 0.001**	13.9	11	27.273	77	21.377
		R04016380993	LOC_Os04g27720	4	0.011*	7.6	24	21.75	65	23.077
	Chr6:0.03–2.03 Mb	BPH32-SNP	LOC_Os06g03240	6	0.436	na	na	na	na	na
TPY	QBPH4.1	OsLecRK3-SNP	LOC_Os04g12580	4	< 0.001**	16.4	16	24.188	76	19.316
	QBPH4.2	OsSTPS2_21bp_indel	LOC_Os04g27430	4	0.007**	8.5	11	23.636	77	19.532
	Chr6:0.03–2.03 Mb	BPH32-SNP	LOC_Os06g03240	6	0.966	na	na	na	na	na
UBN	QBPH4.1	OsLecRK3-SNP	LOC_Os04g12580	4	< 0.001**	43.7	16	27.25	76	16.908
		R04006659915	LOC_Os04g12110	4	0.033*	6.3	10	19.7	72	18.556
	QBPH4.2	OsSTPS2_21bp_indel	LOC_Os04g27430	4	< 0.001**	29.8	11	26.636	77	17.26
		R04015916153	LOC_Os04g26910	4	0.033*	5.3	20	17.6	62	18.339
		R04016380993	LOC_Os04g27720	4	0.043*	4.7	24	17.542	65	19.462
	Chr6:0.03–2.03 Mb	BPH32-SNP	LOC_Os06g03240	6	0.059	na	na	na	na	na

*Significant at *P* = 0.05

**Significant at *P* = 0.001

### Reverse screening of JHN mutant population for BPH resistance using SNP markers from *OsLekRK3* and *OsSTPS2*

To confirm the functional impact of SNP and 21-bp indel markers in *OsLecRK3* and *OsSTPS2* genes, respectively, we screened 9323 JHN mutant lines for polymorphisms in *OsLecRK3* and *OsSTPS2* genes. This analysis identified 11 mutant lines carrying resistant alleles of both *OsLecRK3* and *OsSTPS2* genes (Table 4), three lines carrying the resistant allele of only *OsLecRK3*, and five lines carrying the resistant allele of only *OsSTP2*. All of these 19 mutant lines along with wild-type (WT) JHN and three WT-like mutant lines were evaluated for BPH resistance with three critical BPH populations, including Ubon Ratchathani (UBN), Ta Phraya (TPY), and Chinart (CNT) (Table 4 and Additional file 2: Figure S3). The UBN BPH population was used as the representative of BPH from the rainfed rice farming area in the Northeastern region of Thailand while the TPY and CNT BPH populations were used as the representative of BPH from the irrigated rice farming area in the Central region of Thailand.

**Table 4:** Genotyping data at two loci used for reverse screening of selected mutant lines and BPH damage AUC tests using 3 BPH populations. The “Ins” indicated insertion allele while “Del” indicated deletion allele of the OsSTPS2-21 bp-indel marker. Letter a, b, c, d, e, f, g indicated similarity of the traits in multiple comparison tests

	QBPH4.1	QBPH4.2	BPH damage AUC		
	OsLecRK3-SNP	OsSTPS2-21 bp-indel	CNT	TPY	UBN
RH	G	Ins	6^a^	7^a^	16^a^
JHN4	G	Ins	10^a^	8^a^	17^a^
JHN12005	G	Ins	13^a^	11.33^ab^	44^c^
JHN19525	G	Ins	30^b^	13.5^ab^	41^c^
JHN09962	G	Ins	45^c^	22.5^b^	27.5^ab^
JHN19874	G	Ins	46^c^	43^cd^	57.67^de^
JHN07766	G	Ins	47^c^	40^c^	61^de^
JHNMT2	A	Ins	50^c^	42^cd^	56.33^de^
JHN11183	G	Del	51^c^	49^cdef^	39^c^
JHNMT1	A	Ins	51^c^	38.33^c^	48.67^d^
JHN05678	G	Ins	51^c^	47^cde^	63^e^
JHN19572	G	Ins	52^c^	52.33^defg^	57.67^de^
JHN21689	G	Ins	52^c^	59^efg^	61.67^e^
JHN16065	A	Ins	54^c^	60.33^g^	59^de^
JHN02313	G	Del	54^c^	61.67^g^	60.33^de^
JHN15723	A	Del	54^c^	63^g^	60.33^de^
JHN18131	A	Del	54^c^	59.33^fg^	60.33^de^
JHN	A	Del	54^c^	63^g^	61.67^e^
JHN19671	A	Ins	54^c^	63^g^	61.67^e^
JHN12686	A	Ins	54^c^	63^g^	63^e^
JHN17767	A	Del	54^c^	60.33^g^	63^e^
JHN19577	G	Ins	54^c^	63^g^	63^e^
JHN19578	G	Ins	54^c^	54^defg^	63^de^
JHN21688	G	Del	54^c^	63^g^	63^e^

Four mutant lines (JHN4, JHN12005, JHN19525, and JHN09962) showed resistance to BPH in the BPH infestation test (Table 4). The mutant line JHN4, like RH, showed resistance to all three BPH populations. The mutant line JHN12005 showed strong resistance to CNT and TPY populations but only moderate resistance to UBN population. The third mutant line, JHN19525, showed strong resistance to TPY and moderate resistance to CNT and UBN. The last mutant line, JHN09962, was resistant to TPY and UBN but susceptible to CNT. Interestingly, JHN19874, JHN07766, and seven other mutant lines carried the same mutations in *OsLecRK3,* and *OsSTPS2* genes as RH but were susceptible to all three BPH populations. This observation indicates that *OsLecRK3* and *OsSTPS2* genes may not fully explain the broad-spectrum resistance to BPH in the FN-induced mutants based on the polymorphic markers, or either RH or FN-mutants may harbor additional R gene besides these two genes.

**Fig. 3:**
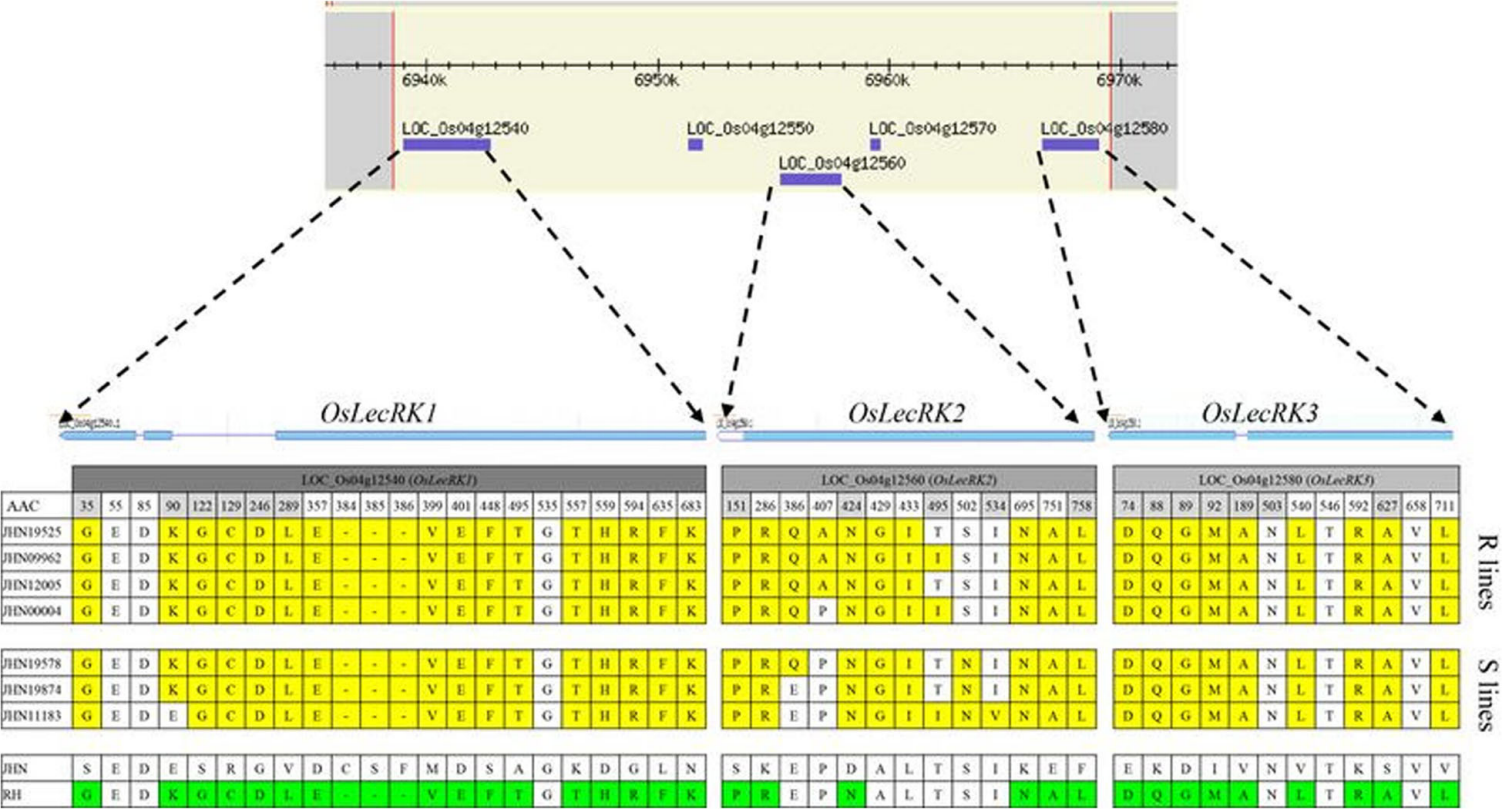
Amino acids analysis of *OsLecRK1–3* in JHN WT, mutant lines and BPH-resistant variety Rathu Heenati (RH). Polymorphic sites are positioned relative to the first amino acid in RH. The sequence information for *OsLecRK1–3* of RH are from the GenBank database (accession no. AIE56222, AIE56230, and AIE56238 respectively). Amino acid changes were highlighted in yellow, and amino acid that JHN WT differed from RH were highlighted in green. The amino acid positions that are corresponding to KASP-SNP markers used for haplotype pattern analysis of mutant lines were highlighted by lighted-gray

Sequence analysis of the *OsLecRK3* gene, the target of reverse screening, showed that four resistant mutant lines (JHN19525, JHN09962, JHN12005, JHN4), three susceptible mutant lines (JHN19578, JHN19874, JHN11183), and RH shared nine synonymous amino acid substitutions (Fig. 3 and Additional file 3: Data S1). Furthermore, extending SNP mining into the adjacent genes (*OsLecRK1* and *OsLecRK2*) revealed similar trends as *OsLecRK3*; the amino acid sequence of resistant and susceptible lines exhibited greater homology with RH than with the WT (Fig. 3). In OsLecRK1, 16 amino acid substitutions, and three amino acid insertions were shared between the resistant lines, susceptible lines, and RH (Additional file 3: Data S2), whereas in OsLecRK2, six amino acid substitutions were shared among these genotypes. Interestingly, novel amino acids were identified at positions 386, 407, 429, 433, 495, 502, and 534 of the OsLecRK2 protein, with mutants differing from both the WT and RH (Additional file 3: Data S3). These seven novel amino acids, found only in OsLecRK3-positive mutants, ruled out the possibility of contamination or outcrossing with RH during seed propagation. In particular, amino acid change at positions 534 was located within the protein kinase domain. Greater homology was detected in the nucleotide sequence of *OsLecRK1–3* and the encoded amino acid sequence among mutants and RH compared with the WT. However, only four mutant lines were BPH resistant. Thus, we speculated that other genetic factors contributed to the BPH resistance of the mutants. Furthermore, the mutant line JHN4 was identified as a promising new BPH resistance donor for pyramiding genes to obtain broad-spectrum BPH resistance in rice.

### Mutant lines carrying *BPH32* are susceptible to local BPH populations

To determine whether *BPH32* imparts BPH resistance in the JHN mutant population, the SNP in *BPH32* was used for reverse screening 9323 randomly selected mutant lines in the M_6_ generation. A total of 16 mutant lines carrying the resistant allele of *BPH32* and susceptible alleles of *OsLecRK3* and *OsSTPS2* were identified. To determine if these BPH32-positive mutant lines showed broad-spectrum resistance, seeds of all 16 lines were retrieved from the mutant seed stock for BPH-infestation test. Unfortunately, only five lines could be germinated and exposed to three BPH populations. As a result, all the lines tested were found to be susceptible to all BPH-populations tested (Additional file 1: Table S5). To find out if the selected mutants carried new SNPs in the *BPH32*, the genomic sequence of the *BPH32* gene of JHN wild-type, selected mutants, and RH were compared. However, no new polymorphic SNPs were found (data not shown), suggesting that the gene had no contribution to BPH resistance in the mutant population. These data suggested that the genetic basis of broad-spectrum BPH resistance in the JHN mutant is complex and different from that in RH-derived BIL populations. Thus, genes other than *OsLecRK*, *OsSTPS2*, and *BPH32* likely confer resistance to BPH in the JHN mutant population.

### HP analysis identified significant genes for BPH resistance of mutants

Single marker analysis unequivocally revealed the molecular marker exhibiting a strong association with broad-spectrum resistance against multiple BPH populations. To investigate whether multiple SNPs determined broad-spectrum resistance, previously developed SNP markers from QBPH4.1 and QBPH4.2, together with known BPH resistance gene-specific SNP markers, were explored for HP mining. SNP markers from previously identified BPH resistance genes were developed for *BPH14* (Du et al. 2009), *BPH3* (Liu et al. 2014), *BPH26* (Tamura et al. 2014), *BPH29* (Wang et al. 2015), *BPH 32* (Ren et al. 2016), and *BPH9* (Zhao et al. 2016) (Additional file 1: Table S6). These SNP markers were used to genotype BPH resistant and susceptible mutants to identify HPs associated with broad-spectrum BPH resistance in mutant lines.

Among the 15candidate genes identified in QBPH4.1 and QBPH4.2 windows and six known BPH resistance genes, 62 SNPs were identified, with 1–8 polymorphic SNPs per gene (Additional file 4: Table S7). No SNPs were identified in *BPH14* or *BPH32* gene, which implied that the two genes were fixed in the selected mutants. The WT HP was designated as HP1, and alternative HPs were then identified. The number of HPs varied from 2 to 10; two HPs were identified in the gene encoding inorganic phosphate transporter protein (LOC_Os04g10800), and 10 HPs were identified in the *OsLecRK3* gene. These mutants were evaluated for BPH damage AUC against three BPH populations. Therefore, it was possible to associate the HPs of candidate genes with BPH resistance (Table 5).

**Table 5:** Association between candidate genes with BPH resistance in mutant lines based on HPs using linear regression and ANOVA. Three BPH populations were used in this study, i.e., CNT, TPY, and UBN. KASP-SNP markers using for HP specification were indicated for each gene

BPH	Gene	*P* value	PVE (%)	Significant HPs	Haplotype patterns						Mean AUC	count	Damage reducing ratio (DRR)
CNT	F-box 119	0.039*	24.8	2	KASP-SNP	R04006388017	R04006388095						0.688
					mutant	Hetero	G				36.667	3	
					wt	C	G				53.25	12	
	BPH9_Chr12	0.005**	40.6	2	KASP-SNP	R1222886067	R1222886105						0.535
					mutant	G	T				28.333	3	
					wt	A	G				52.923	13	
TPY	inorganic phosphate transporter	0.047*	13.5	1 (WT)	KASP-SNP	R04005867219							1.373
					mutant	G					58.951	7	
					wt	C					42.937	16	
	gamma thionin protein	0.012*	38.2	2	KASP-SNP	R04006069830	R04006069877	R04006069900					0.556
					mutant	G	G	G			31.08	8	
					wt	G	A	A			55.78	9	
	F-box 118	0.01**	30.9	3	KASP-SNP	R04006263057	R04006263087	R04006263168					0.581
					mutant	G	T	G			33.707	4	
					wt	C	A	C			57.999	12	
	F-box 119	0.005**	40.4	3	KASP-SNP	R04006388017	R04006388095						0.506
					mutant	A	C				29.332	5	
					wt	C	G				57.999	12	
	LR10	0.046*	26.7	2	KASP-SNP	R04006451404	R04006451424	R04006451469	R04006451485	R04006451495			0.602
					mutant	C	A	A	G	G	34.63	9	
					wt	T	C	G	A	T	57.54	11	
	OsSTPS2	0.006**	38.6	2	KASP-SNP	OsSTPS2_SNP1	OsSTPS2_SNP2	OsSTPS2_SNP3	OsSTPS2_21bp_indel				0.604
					mutant	G	T	C	Insertion		36.25	12	
					wt	G	T	C	deletion		60	6	
	Verticillium wilt resistance protein	0.020*	19.6	2	KASP-SNP	R04016672214							0.688
					mutant	T					42.764	17	
					wt	G					62.112	6	
	BPH9_Chr12	< 0.001**	71	2	KASP-SNP	R1222886067	R1222886105						0.254
					mutant	G	T				14.667	3	
					wt	A	G				57.667	13	
UBN	BPH9_Chr12	< 0.001**	83.3	2	KASP-SNP	R1222886067	R1222886105						0.464
					mutant	G	T				28.5	3	
					wt	A	G				61.411	13	

*Significant at P = 0.05

**Significant at P = 0.001

Single marker analysis of 21 candidate gene HPs from QBPH4.1, QBPH4.2, and BPH resistance genes revealed eight genes in which HPs were significantly associated with BPH resistance in the selected mutants, with phenotypic variance ranging from 13.5% to 83.3% (Table 5). Interestingly, the most significant association with BPH resistance was identified in HP2 of *BPH9* with phenotypic variance in BPH resistance of 83.3%, 71.0%, and 40.6% against UBN, TPY, and CNT populations, respectively. SNPs at nucleotide positions 274 (A:G, WT:mutant) (SNP id. BPH9_Chr12_22,886,067) and 258 (G:T, WT:mutant) (SNP id. BPH9_Chr12_22,886,105) in exon 1 of *BPH9* generated four HPs (Additional file 1: Table S6). The A-G and G-T HPs were associated with susceptibility and resistance, respectively, to all BPH populations. By substituting the susceptible A-G with the resistant G-T HPs, the damage reducing ratios (DRRs) were 0.53, 0.46, and 0.25 against CNT, UBN, and TPY populations, respectively (Table 5). The replacement of A-G HP with G-T HP resulted in one amino acid substitution from arginine (R) in WT and susceptible mutants to glycine (G) in resistant mutants, as the mutation at nucleotide position 258 resulted in synonymous substitution.

The second highest impact HP was linked to the two F-Box genes, *F-Box118* and *F-Box119*. For F-Box119, two SNPs, resulting in non-synonymous substitutions, were identified at nucleotide positions 837 (SNP id. F-box_R04006388017) and 759 (SNP id. F-box_R04006388095) of the single exon gene, generating four HPs (Additional file 1: Table S6). These SNPs caused two amino acid substitutions from lysine (K) and aspartate (D) to asparagine (N) and glutamic acid (E), respectively. The C-G HP was associated with susceptibility, while the A-C HP was associated with resistance against TPY and H (heterozygous)-G HP was associated with resistance against CNT (Table 5). In the *F-Box118* gene, three SNPs generated three HPs (Additional file 1: Table S6). These SNPs caused three non-synonymous amino acid substitutions from glutamine (Q), asparagine (N), and leucine (L) to glutamic acid (E), tyrosine (Y), and valine (V), respectively. The C-A-C and G-T-G HPs were associated with susceptible and resistance, respectively, to the TPY population. Consequently, the DRR of haplotype substitutions in *F-Box118* and *F-Box119* genes were 0.58 and 0.50, respectively, against the TPY population (Table 5).

The third most significant HPs were specifically associated with the TPY population. Three promoter-specific SNPs and the functional 21-bp indel marker created four HPs in the *OsSTPS2* gene (Additional file 1: Table S6). The G-T-C-21 bp_insertion were associated with BPH resistance against the TPY population. The 21-bp insertion in the *OsSTPS2* gene together with the WT HP in its promoter increased resistance to BPH, with a DRR of 0.60 against the TPY population (Table 5). Other significant genes which reduced BPH damage of TPY population were the gamma thionin protein (LOC_Os04g11165), LR10, and VWR protein with DRRs of 0.56, 0.60, and 0.69, respectively. However, a mutation in the gene encoding inorganic phosphate transporter protein increased the BPH damage AUC by DRR of 1.37 against TPY infestation.

### Genes that play a dominant role in broad-spectrum BPH resistance

The *BPH9* gene was considered the most significant gene in the selected mutant lines. Four HPs were identified in mutant lines. The HP2 of *BPH9* had the most substantial impact on the DRR. Three resistant mutant lines (JHN4, JHN19525, and JHN09962) were identified as carrying *BPH9* HP2 (Table 6). The JHN4 mutant was resistant to all BPH populations tested (CNT, TPY, and UBN). On the other hand, JHN19525 showed strong resistance to TPY and moderate resistance to CNT and UBN populations, whereas JHN09962 was resistant to TPY and UBN but susceptible to CNT. Four mutant lines, namely JHN12005, JHNMT1, JHNMT2, and JHN11183, carried HP3 at *BPH9* (Table 6); however, only JHN12005 was strongly resistant to CNT and TPY and moderately resistant to UBN. Three mutant lines carried HP4 at *BPH9* including JHN19874, JHN18131, and JHN12686 were susceptible to all BPH infestation tested.

**Table 6:** HP analysis of significant genes in mutant lines. Number 1 to 10 represented HP number of each gene by which HP number 1 was specific to WT (Additional file 1: Table S6). R = resistance, MR = moderately resistance, S = susceptible

KASP marker name	inorganic phosphate transporter	gamma thionin protein	F-box 118	F-box 119	LR10	OsLecRK1	OsLecRK2	OsLecRK3	OsSTPS2	Verticillium wilt resistance protein	BPH32	BPH9	BPH -CNT	BPH -TPY	BPH- UBN
RH	1	2	3	3	2	2	3	2	2	1	2	1	R	R	R
JHN	1	1	1	1	1	1	1	1	1	1	1	1	S	S	S
JHN4	1	2	2	2	2	2	2	2	2	2	1	2	R	R	R
JHN12005	1	2	3	3	2	3	3	2	2	2	1	3	R	R	MR
JHN19525	1	2	2	3	2	2	3	2	2	2	1	2	MR	R	MR
JHN09962	1	2	3	3	2	2	2	2	2	2	1	2	S	R	R
JHN19874	1	3	2	2	3	–	–	3	2	2	1	4	S	S	S
JHN07766	1	2	2	4	2	3	4	2	2	2	1	1	S	S	S
JHNMT2	1	1	1	1	1	4	5	4	2	2	1	3	S	S	S
JHN11183	2	1	2	4	4	5	6	5	1	2	1	3	S	S	MR
JHNMT1	1	1	1	1	1	4	5	4	2	2	1	3	S	S	S
JHN05678	1	2	3	3	2	2	2	2	2	2	1	1	S	S	S
JHN19572	1	2	2	3	2	2	3	2	2	2	1	1	S	S	S
JHN21689	2	1	1	1	1	1	1	6	1	1	1	1	S	S	S
JHN16065	2	4	1	1	1	1	1	7	3	2	1	1	S	S	S
JHN02313	2	1	1	1	1	6	7	8	4	1	1	1	S	S	S
JHN15723	1	1	1	1	1	1	1	1	1	1	1	1	S	S	S
JHN18131	2	5	1	1	1	1	1	9	4	2	1	4	S	S	S
JHN19671	2	4	1	1	1	1	1	9	3	2	1	1	S	S	S
JHN12686	1	1	1	1	5	–	8	1	1	1	1	4	S	S	S
JHN17767	2	4	1	1	1	–	1	9	4	2	1	1	S	S	S
JHN19577	1	3	2	4	2	2	3	2	2	2	1	1	S	S	S
JHN19578	1	2	3	2	2	2	3	2	2	2	1	1	S	S	S
JHN21688	1	1	1	1	1	1	1	10	1	1	1	1	S	S	S

Five mutant lines (JHN07766, JHN05678, JHN19572, JHN19577, and JHN19578) harbored HPs at significant genes on chromosome 4 similar to those in four resistant lines (JHN4, JHN12005, JHN19525, and JHN09962); however, these five mutants showed susceptibility to all BPH populations tested because their HP on *BPH9* gene was WT (HP1). This observation confirmed *BPH9* as the most important BPH resistance gene in the mutant population (Additional file 1: Table S8).

Therefore, mutations in *BPH9* as well as in inorganic phosphate transporter, gamma thionin, F-Box118, F-Box119, LR10, *OsSTPS2,* and VWR genes (all on chromosome 4) were crucial for obtaining broad-spectrum resistance in the JHN mutant population. RH, the donor of broad-spectrum BPH resistance, carried HP2 at *BPH32* and HP2 or HP3 at all 7 significant genes on chromosome 4. Furthermore, HPs of significant genes for five extreme resistant BILs and susceptible BILs were reported (Table 7) and showed that *OsLecRK2*–*3* and *OsSTPS2* played significant roles in BPH resistance in these BILs, as every BPH resistant BIL carried HPs 3, 2, and 2 in *OsLecRK2*, *OsLecRK3*, and *OsSTPS2* respectively. On the other hand, the *BPH32* gene possibly played interactive roles depending on the HPs of *OsLecRK2*–*3* and the *OsSTPS2* genes conferring broad-spectrum BPH resistance in RH and RH-derived BILs. Moreover, genes encoding inorganic phosphate transporter protein, the gamma thionin protein, F-box118, F-box119, and LR10 may not play a role in the BPH resistance of BILs since the HP at these genes in the resistance BIL 423(4) was different from that in RH. Therefore, mutations in *OsLecRK2*–*3* and *OsSTPS2* were crucial for broad-spectrum resistance to BPH in RH and BIL populations; this suggests that breeding for broad-spectrum BPH resistance must aim to accumulate multiple BPH resistance genes from chromosomes 4, 6, and 12.

**Table 7:** HP analysis of significant genes in 5 extreme resistant-BILs and five extreme susceptible-BILs. Number 1 to 4 represented HP numbers of each gene by which HP number 1 was specific to JHN WT (Additional file 1: Table S6). Six BPH local populations including KPP, NAN, PSL, UBN, TPY, and HTL were used for BPH damage AUC test. - = missing data

BIL no.	inorganic phosphate transporter	gamma thionin protein	F-box 118	F-box 119	LR10	OsLecRK1	OsLecRK2	OsLecRK3	OsSTPS2	Verticillium wilt resistance protein	BPH32	BPH9	BPH damage AUC							
													KPP	NAN	PSL	UBN	TPY	HTL	Average	BPH resistance
RH	1	2	3	3	2	2	3	2	2	1	2	1	12.3	17.3	10.3	9.7	9.7	8.3	11.3	R
UBN03078–101–342-4-147	1	–	–	3	2	2	3	2	2	1	2	1	12	12	14.3	9.3	14.7	21	13.9	R
423(4)	2	4	1	1	1	1	3	2	2	2	2	1	11.7	16.7	13.7	11	16	18.3	14.6	R
UBN03078–101–342-4-20	1	2	3	–	–	2	3	2	2	–	2	1	12	23	12.3	12	14.3	14.3	14.7	R
UBN03078–101–342-4-138	–	2	3	3	2	2	3	2	2	1	2	1	12.3	21	15.3	15.7	15.3	10	14.9	R
UBN03078–101–342-4-24	1	2	3	3	–	2	3	2	2	1	2	1	12	15.3	16.7	12	14.7	19.3	15	R
UBN03078–81–504-1	–	–	3	1	2	2	1	1	H	1	2	1	25.3	35.7	36	32.7	26.7	34.3	31.8	S
UBN03078–80–354-7	1	–	–	–	–	2	1	1	1	1	2	–	29.7	31	31.7	35.7	30	31.3	31.6	S
UBN03078–80–354-7	1	–	–	1	–	2	1	1	1	1	1	1	35.7	30.3	32	32	22	35.7	31.3	S
UBN03078–101–450-1	1	–	3	1	–	2	1	1	1	1	2	1	25.7	33.7	27.3	28.7	29	33	29.6	S
UBN03078–80–28-5	1	2	3	1	2	2	1	1	H	1	2	1	19	36	36	29	25.3	28	28.9	S
KDML105	2	4	1	1	1	1	1	1	1	2	1	1	34	36	36	35.3	30.3	36	34.6	S

### The origin of the BPH resistant JHN mutant line

**Fig. 4:**
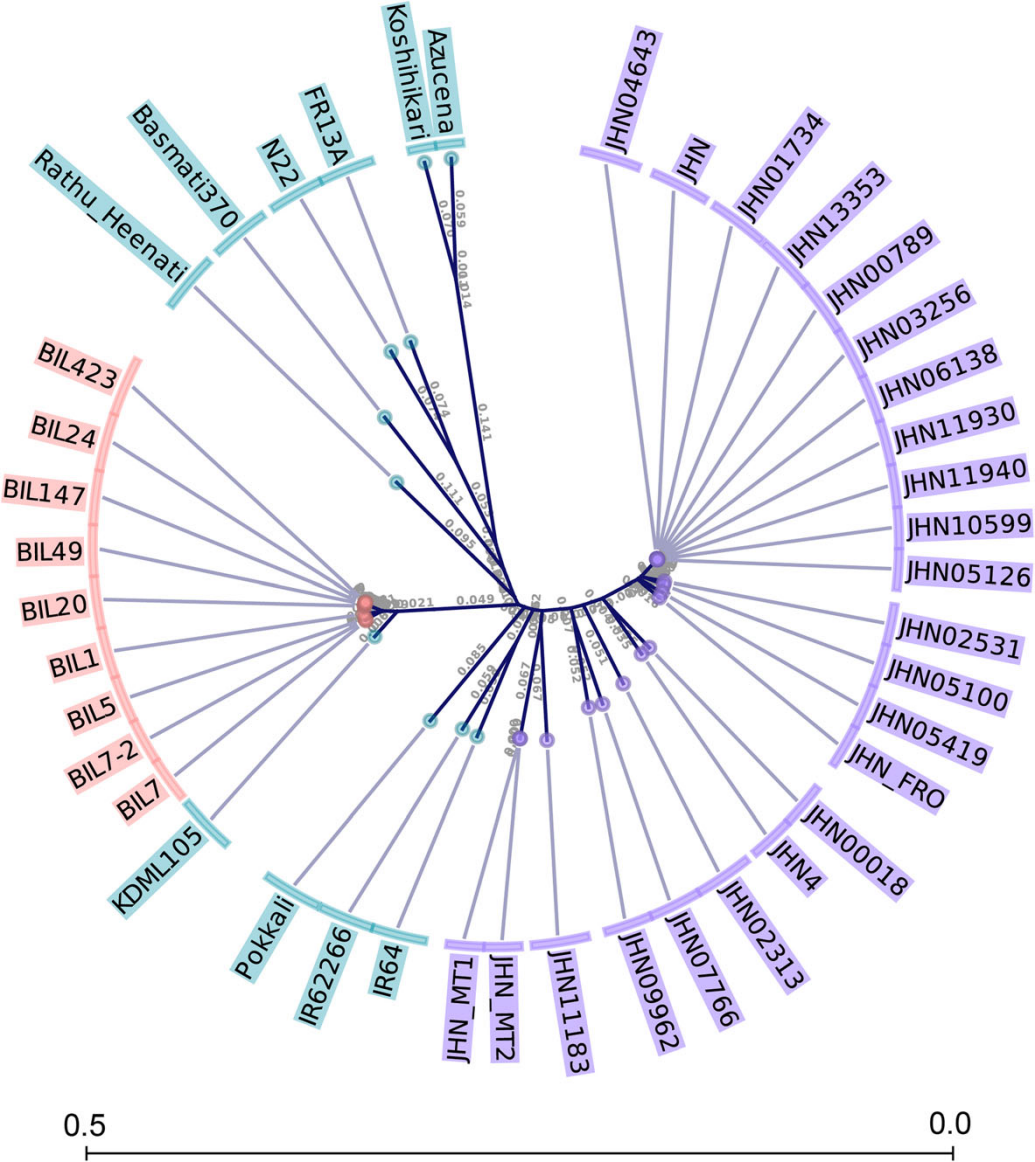
Phylogenetic analysis of the mutant lines, JHN-WT, KDML105, RH, BILs, and other germplasm rice accessions. The evolutionary history was inferred using the UPGMA method

**Fig. 5:**
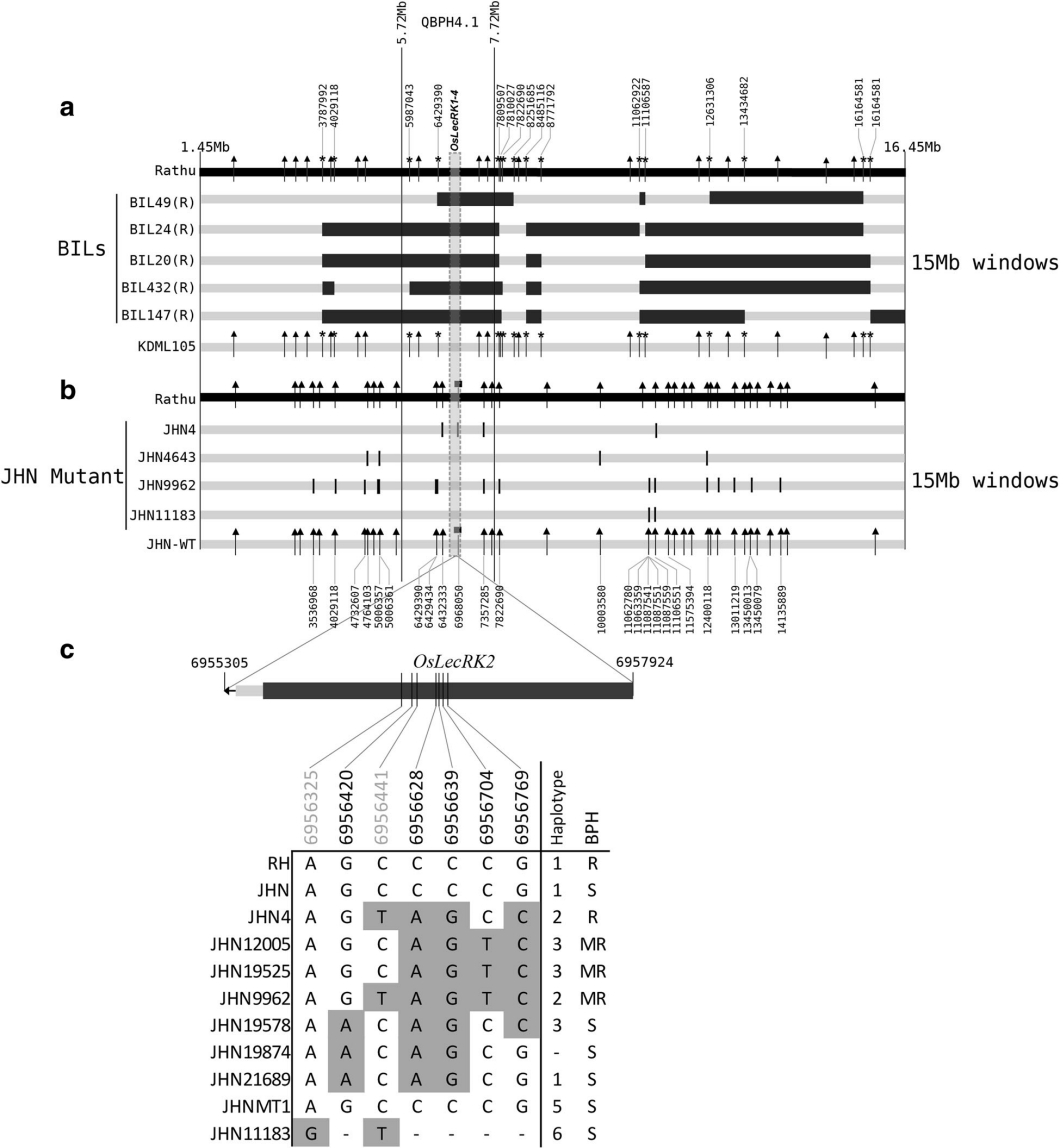
Graphical genotype analysis of 15 Mb spanned QBPH4.1 genomic region. **a** The extreme resistant BILs were analyzed with KDML105 and RH, **b** The JHN mutant lines were analyzed with JHN-WT and RH, **c** Haplotype patterns at seven novels amino acids of mutant lines. SNPs at position 6,956,325 and 6,956,441 were developed as the KASP markers for HP analysis. Filled squares represented gene-specific SNPs, filled arrowheads represented intergenic SNPs, and asterisks represented the recombination breakpoint

To investigate whether BPH resistance found in mutant lines were induced by FN or contamination during mutant population advancement, two pieces of evidence were generated. Based on 8928 genome-wide SNPs, phylogenetic analysis clearly showed four clusters related to JHN, BILs, RH, and the japonica out-group (Fig. 4). The analysis separated the BPH resistant genotypes, including JHN4, resistant BILs, RH, and Pokkali, into different clusters. This result showed that JHN4 and RH were originated independently and ruled out the possibility of the contamination of BPH resistant donors in the JHN mutant population seed stock. We further examined the genomic structure of QBPH4.1 spanning 15 Mb. Four BILs were derived by backcrossing RH with KDML105. With more than 33 informative SNP markers, several large RH-derived DNA fragments ranging from 241 kb to 5.1 Mb were detected (Fig. 5a). The total span of genomic region introgressed from RH ranged from4.9 Mb in BIL49 to11.7 Mb in BIL24. By contrast, very small homologous sequences were identified in the resistant (JHN4) and moderately resistant (JHN09962) mutants (Fig. 5b). Additionally, mutants carried either the same haplotype as that in JHN WT or unique HP within the *OsLecRK2* CDS (Fig. 5c). Four SNPs (positions 6,956,441; 6,956,628; 6,956,639; and 6,956,769) created novel amino acids (positions 495, 433, 429, and 386 respectively) in the *OsLecRK2* gene of JHN4, which were not found in either RH or JHN WT (Fig. 3). Together with its distinguish seed color of dark purple, BPH resistance identified in JHN4 is not contaminated from a pollen or seed source but induced by FN.

## Discussion

### QTL-seq/ddRADseq identified candidate genes for BPH resistance

In this study, we successfully combined QTL-seq with ddRADseq to identify genomic region harboring BPH resistance genes from rice cultivar RH. The two genomic regions, QBPH4.1 and QBPH4.2, were identified on rice chromosome 4. For the QBPH4.1 region, two reportedly candidate genes and one cloned gene for BPH resistance were localized (Kamolsukyunyong et al. 2013; Liu et al. 2014). These genes were LOC_Os04g11660 and LOC_Os04g11780, encoding F-box 119 and LR10 proteins (Kamolsukyunyong et al. 2013) and LOC_Os04g12580, encoding the *OsLecRK3*, one of the members of the gene cluster (*OsLecRK1*–*3*) responsible for BPH resistance in RH (Liu et al. 2014). For the QBPH4.2 region, the most interesting candidate gene is LOC_Os04g27430, encoding the *OsSTPS2* gene, reportedly to play essential roles in antixenosis resistance mechanisms against BPH (Kamolsukyunyong et al. 2013).

The *BPH32* gene (LOC_Os06g03240) has been mapped to an area between the simple sequence repeat markers RM589 and RM588 and served as one of the primary target loci in RH as BPH resistance donor for marker-assisted backcrossing in this BIL population (Jairin et al. 2009). Our QTL-seq analysis could not identify significant SNP with statistical confidence in this gene (Fig. 2a, b). This observation may reflect the minor effect of *BPH32* gene on BPH resistance in the RH-derived BIL population. By using known BPH32-specific SNP (Ren et al. 2016), only 5 out of 16 BPH32-positive lines could be tested for BPH resistance in this study and found that all tested mutants were BPH susceptible (Additional file 1: Table S5). Sequence comparison confirmed that no other new mutation was found in the *BPH32* gene. As such the gene is not essential for BPH resistance in the JHN-mutagenized population.

Our results confirmed that for the durable BPH resistance rice variety RH, not only single genes/ gene cluster of *OsLecRK*s, *OsSTPS2*, or *BPH32* is essential for BPH resistance, but all of them working together in response to BPH infestation. As we shown in Table 3, SNP marker from *OsLecRK3* and indel marker from *OsSTPS2* associated with resistance to all the BPH populations tested but SNP from *BPH32* associated with resistance to only one BPH population. This result may suggest that the gene cluster *OsLecRK*s, and the gene *OsSTPS2* play a major role while the gene *BPH32* play a minor role for BPH resistance in RH. As the single marker analysis may not fully explain the effect of these genes, our HP analysis of the extreme resistance and susceptible BILs may better support this speculation. It showed that HP2 of *BPH32* is useful when HPs at *OsLecRK2*, *OsLecRK3*, and *OsSTPS2* were 3, 2, and 2 respectively (Table 7). On the other hand, HP2 of *BPH32* will not be effective when the HPs of those three genes were not in the said pattern.

### Breeding for broad-spectrum resistance requires multiple gene haplotypes

Improving broad-spectrum resistance creates a solid foundation for cultivars with durable resistance against highly variable insects such as BPH. To date, at least 25 major *BPH* genes located as gene clusters on chromosomes3, 4, 6, and 12 have been identified (Jing et al. 2017). Durable BPH resistance comprises the recognition of herbivore/damage-associated molecular patterns (HAMPs/DAMPs), and the activation of resistance genes encoding the coiled-coil nucleotide-binding leucine-rich repeat (CC-NB-LRR) domain proteins (Jing et al. 2017).To develop new rice varieties with durable BPH resistance, molecular markers must be developed for effective pyramiding of genes with strong resistance to BPH. Pyramiding *BPH14* (a CC-NB-LRR protein) and *BPH15* (a LecRK protein) enabled the successful development of a hybrid rice cultivar with broad-spectrum BPH resistance (Hu et al. 2012). In this study, two major QTLs on rice chromosome 4 (QBPH4.1 and QBPH4.2) harboring *OsLecRK* genes (Liu et al. 2014) and *OsSTPS2* genes (Kamolsukyunyong et al. 2013), respectively, and a minor QTL harboring *BPH32* on chromosome 6 (Jairin et al. 2009; Ren et al. 2016) were shown to be involved in broad-spectrum BPH resistance derived from RH. Pyramiding QBPH4.1, QBPH4.2, and *BPH32* into KDML105 by marker-assisted backcrossing has successfully improved broad-spectrum BPH resistance against diverged BPH populations (Vanavichit et al. 2018). Reverse genetics using the JHN mutant population revealed comprehensive mutations within the two gene clusters on chromosome 4 together with *BPH9*, enabling the induction of a mutant line exhibiting broad-spectrum BPH resistance. We have previously demonstrated the role of insect-inducible volatile factors, including sesquiterpenes and monoterpenes (Pitija et al. 2014), in antixenosis mechanisms supporting durable resistance against BPH populations. In this study, we identified the role of at least eight genes harboring 22 SNPs in broad-spectrum BPH resistance in JHN mutant lines (Table 5). These mutant lines can be used as the donor in the breeding program. However, pyramiding these eight genes or 22 SNPs into one variety using marker-assisted analysis may not be efficient. Our results showed the organization of these target SNPs into eight gene-specific HPs. These results would empower breeders to design a more efficient gene pyramiding program for developing rice cultivars with strong BPH resistance.

### Genome background relationship of JHN and RH

Based on our phylogenetic analysis using SNPs derived from ddRAD, it was clearly illustrated that JHN, BILs, and RH were separated into different clusters. While RH was grouped with Basmati370, N22, and FR13A from South Asia, JHN and 22 JHN M_6_ mutant lines were grouped together. Also, the RH-derived BILs were grouped with KDML105, the recipient parent. The phylogenetic analysis reflected no relationship on the genome background between RH and JHN mutants and thus harboring a different set of BPH resistant genes in selected JHN mutant lines is possible. As reported by Zhao et al. (2016), RH contained HP1 susceptible allelotype at *BPH9* gene, and we also showed that the *BPH9* (HP1) was not essential for BPH resistance in RH-derived BILs (Table 7). On the other hand, as we showed in Table 5, the HP2 of *BPH9* was associated with BPH resistance of mutants in all three BPH populations tested by reducing BPH damage as DRR of 0.535, 0.245, and 0.462 for CNT, TPY, and UBN respectively.

To confirm if the HP2 of *BPH9* is genuinely crucial for BPH resistance in JHN mutant population, the 9313-mutant-lines were screened for HP2 mutation of *BPH9* gene. As a result, there were three more mutant lines found to harbor HP2 of *BPH9* gene. These mutant lines were JHN19727, JHN22247, and JHN22995. HPs of 21 candidate gene from QBPH4.1, QBPH4.2, and BPH resistance genes of these mutant lines were also characterized (Additional file 4: Table S7). Multiple BPH resistance of these lines will be evaluated in the future.

### Rapid induction of spontaneous mutations using FN mutagenesis

FN mutagenesis typically generates deletions and chromosome rearrangements at a genome-wide scale (Gilchrist and Haughn 2010). The induction of multiple nucleotide substitutions is another essential characteristic of FN-induced mutagenesis (Belfield et al. 2012). In the model rice cultivar Kitaake, FN at 20 Gy resulted in single base substitution (SBS), deletion, insertion, translocation, and tandem duplication in the M_2_ population (Li et al. 2017). Because of the high frequency of deletions, loss-of-function mutations are the primary cause of mutation detection in most FN-mutagenized populations (Li et al. 2001, 2002). In the M_2–3_ population developed from FN mutagenesis of Kitaake, SBSs account for 47.5% of all mutations (Li et al. 2017). Similarly, in JHN mutant population generated using FN mutagenesis, SBSs, deletions, and insertions accounted for 56%, 23%, and 21% of the total mutations, respectively (data not shown). These data suggest that the genetic basis of mutagenesis was similar in the JHN and Kitaake mutant populations.

Considering the high homology of the induced mutations in the QBPH4.1, QBPH4.2, and *BPH9* to RH and BPH donors, we believe that the genetic variation detected in M_6_ JHN mutant population was caused by FN-induced spontaneous mutations. Our FN-mutagenized population survived two significant forces during development. First, during the early phase, a major loss of lethal mutants may have eliminated all large chromosomal rearrangements from the mutagenized population. Second, the JHN mutant population was supposedly exposed to environmental stresses during generation advancement by self-pollination in an organic paddy field in Thailand. This exposure of an FN-mutagenized population to biotic and abiotic stresses in an open paddy field may impose natural selection and homologous recombination during the prolonged generation advancement time frame. In *Arabidopsis*, abiotic stresses lead to heritable changes in the frequency of recombination, point mutation, and microsatellite mutation (Yao and Kovalchuk 2011). Additionally, the rate of homologous recombination is strongly affected by high temperature, short day length, and moderate change in environmental conditions (Boyko et al. 2005, Kloosterman et al. 2013). On an average, 9.6 spontaneous mutations per line in the M_2–3_ Kitaake rice population contained 7.6 SBSs and two small indels (Li et al. 2016). On the other hand, in the M_6_ JHN mutant population, only 4% of SBSs were novel, based on the comparison with OrzaSNP database. Therefore, higher mutation rate in the JHN mutant population could be due to 1) synergistic effects of selection against lethal mutations at the beginning of the generation advancement, 2) several generations of genetic recombination during long-term self-pollination, and 3) natural selection during generation x advancement. Spontaneous mutations may arise from local genomic lesions and repair, resulting in SBSs and single base frame shift mutations, which occur in a non-random manner at mutation hotspots throughout the genome (Maki 2002). The induced broad-spectrum BPH resistance identified in JHN4 may represent several mutation hotspots induced by FN mutagenesis and natural exposure to local BPH populations during long-term generation advancement via self-pollination.

Transposable elements (TE) are also sensitive to irradiation and environmental stresses. Stress-responsive *Arabidopsis* mutant lines acquired exapted TE-derived genes coding for new proteins, thus gaining new roles for host adaptation to stressful environments, such as high phosphate and arsenic levels, salinity, and freezing (Hoen and Bureau 2015; Joly-Lopez et al. 2017). In mutagenized Kitaake rice, FN-induce TE mutations at 58.6% compared to 25.7% in the same flanking sequence tag population (Hong and Jung 2018). It would be interesting to further explore the possibility whether FN-induced exapted TE transposition in the JHN mutant population. Thus, FN-mutagenized population contains newly induced suites of SBSs and indels as a basis for creating new genetic variability.

## Conclusion

In this study, we used ddRADseq with QTL-seq analysis to identify SNPs and candidate resistance genes associated with BPH resistance in RH-derived BILs. Two major genomic regions associated with broad-spectrum BPH resistance were localized between 5.78–7.78 Mb (QBPH4.1) and 15.22–17.22 Mb (QBPH4.2), forming three linkage disequilibrium (LD) blocks on the rice chromosome 4. Twenty-one significant SNPs were organized into three haplotype blocks: two blocks in QBPH4.1 and one block in QBPH4.2. Functional markers associated with *OsLecRK3* and *OsSTPS2* genes were used for reverse screening of 9323 FN-mutagenized lines generated from the BPH susceptible rice cultivar JHN in the M_6_ generation. Twenty-two mutants, including 19 identified mutants, were evaluated for BPH resistance using three active BPH local populations representing important rice growing areas in Thailand. As a result, one resistant mutant (JHN4) and three moderately resistant mutants (JHN12005, JHN09962, and JHN19525) were identified. Further screening with six known BPH resistance genes revealed that *BPH9* was dominant to *OsLekRK2*–*3* and *OsSTPS2* genes in reducing BPH damage in the mutant population. On the other hand, *OsLekRK2–3*, *OsSTPS2*, and *BPH32* determined the broad-spectrum BPH resistance in RH-derived BILs. Significant gene-specific HPs involved in broad-spectrum BPH resistance in both BILs and JHN mutant population were identified. We demonstrated that long-term FN mutagenesis is a useful tool for generating not only novel but also a natural genetic variation for functional genetics and molecular breeding. Together, our data suggest that RH and the BPH-resistance mutant lines can be used as the donors in a breeding program to improve broad-spectrum resistance of rice crop against diverged BPH populations. Four gene-specific haplotypes including *OsLecRK2*, *OsLecRK3*, *OsSTPS2*, and *BPH32* are needed in the breeding program using RH as a donor. On the other hand, eight gene-specific haplotypes including *BPH9*, inorganic phosphate transporter, gamma thionin, F-Box118, F-Box119, LR10, *OsSTPS2*, and VWR genes are needed in the breeding program using the BPH-resistance mutants as a donor.

## Methods

### Plant materials

The BILs were developed from a cross between KDML105 and the BPH resistant donor, RH, by backcrossing using marker-assisted selection (Jairin et al. 2009). The F_1_ plants were backcrossed with the recurrent parent. BPH resistant BC_1_ and BC_2_ plants were selected, and the BPH3-linked marker RM589 on chromosome 6 where *BPH32* was localized (Ren et al. 2016), was used to generate BC_2_F_1_ and BC_3_F_1_ generations, respectively. Two individual BC_3_F_3_ plants were developed from the resistant BC_3_F_2_ progenies (*n* = 2343) that were heterozygous at the linked marker on chromosome 6. A total of 105 BC_3_F_5_ BILs were generated by self-pollination of selected heterozygous BC_3_F_3_ plants. These BILs were infested with six local BPH populations collected from intensively irrigated and rain-fed rice production areas. BILs those were highly resistant (19 and 5 for pool and individuals respectively) and susceptible (16 and 4 for pool and individuals respectively) to BPH were identified and used for QTL-seq/ddRADseq analysis both as pools and as individuals.

The FN-mutagenized population was developed from the rice cultivar JHN, a photoperiod insensitive, semi-dwarf purple rice variety (Ruengphayak et al. 2015). Contamination of the JHN population can be readily recognized based on the purple grain color. Initially, 100,000 purified seeds derived from a panicle-to-row of a single JHN plant were treated with 33 Gy of FN by the Office of Atoms for Peace, Thailand, in 2012. Starting with the first generation (M_1_), the whole mutant population was field grown; however, with the impact of FN on germination, more than 70% of the population was lost in subsequent generations. In the M_4_ generation, 21,024 lines were identified as stable. Generation advancement of the mutant population followed the same procedure as that used for the development of a random inbred line population, but with slight modifications. For each line, eight plants were randomly selected, and panicles were individually bagged with a pollination glassine envelope, before pollination, and until seed set. Seed-containing panicles were processed individually, and stored individually or as pools. In total, 9313 M_5–6_ mutant lines were used in this study. The reverse screening was conducted using three selected polymorphic SNP/indel markers in *OsLecRK3*, *OsSTPS2*, and *BPH32* genes. The M_5_ mutants that showed the same alleles as RH for these markers were selected, and M_6_ families derived from these M_5_ mutants were used for the BPH infestation test.

### BPH populations and screening

Six local BPH populations collected from six critical rice-growing provinces in Thailand (HTL, KPP, Nan [NAN], PSL, TPY, and UBN) were used for BPH damage screening of BILs while three BPH population (CNT, TPY, and UBN) were used to evaluate the selected mutants using a modified standard seedbox screening method (SSBS) (Heinrichs et al. 1985).

The BILs and candidate mutant lines were assessed for BPH resistance at the seedling stage. Three replications of each line were under greenhouse conditions and arranged in a randomized complete block design. The second or third instar nymphs of the BPH were released on 7–10-day-old rice seedlings at the rate of 8–10 insects per plant. Seedling reaction to BPH was recorded daily as a damage score for five consecutive days, which started 7–10 days after infestation or when the susceptible check Taichung Native1 (TN1) was completely dead. The 5-day-long damage scores were presented as AUC for rating the damage caused by each BPH population. The AUC of damage rating of BPH populations were calculated using the trapezoid rule (Litsinger 1991). The AUC values were used to compare damage rating among 105 BILs and their parents. The Standard Evaluation System for Rice (SES) (International Rice Research Institute 2013) was used to estimate the initial damage caused by BPH populations on a scale of 1 to 9.

### Genotyping-by-sequencing

Genotyping-by-sequencing of BILs and mutants was performed as an individual plant or as a pool of plants using ddRADseq (Peterson et al. 2012, Pootakham et al. 2015). Genomic DNA was isolated from leaves tissue using DNeasy Plant Mini Kit (Qiagen, Carlsbad, CA, USA), and quantified using NanoDrop ND-8000 Spectrophotometer (Thermo Scientific, Wilmington, DE, USA). Resistance and susceptible pools contained 200 ng of DNA from selected BPH resistant lines (*n* = 19) and susceptible lines (*n* = 16). The two pools were used for ddRADseq library construction. In addition, highly resistant lines (*n* = 5) and highly susceptible lines (*n* = 4) were sequenced individually as internal controls. Paired-end sequencing libraries (mean read length of 112 bp) with an insert size of approximately 200 bp were prepared. The DNA libraries were labeled using *Pst*I-adapters containing specific 9-bp barcodes. Libraries were sequenced using the Ion Proton PITM Chip (Life Technologies, Grand Island, NY, USA), according to the manufacturer’s instructions.

### QTL-seq analysis

Short sequence reads from BPH resistant and susceptible pools and extreme BILs were locally aligned to the Nipponbare reference genome (Os-Nipponbare-Reference-IRGSP-1.0 pseudo-molecules), with a fragment size of 80–300 bp, using Bowtie2 Software (Langmead and Salzberg 2012). Description of sequencing data was added by using the command line AddOrReplaceReadGroups of the Picard command line tools (http://broadinstitute.github.io/picard/). Sequence data were improved in the region spanning the indel polymorphisms using the Genome Analysis Toolkit (GATK) (https://software.broadinstitute.org/gatk/), including indel positioning by GATK Realigner Target Creator and re-alignment by GATK IndelRealigner. An improved overlapping sequence of restriction associated DNA (RAD tag), was used for SNV calling using GATK UnifiedGenotyper, with a minimum confidence threshold and emit confidence score of 0.5 and 0.3, respectively. Low-quality RAD tag with a Phred quality score lower than 15 and base quality score less than 20 (Q > 20, 1:100; 90%) were excluded.

The ddRADseq libraries were sequenced at more than 6X coverage, and the identified SNVs were used for SNP-index calculation. The bi-allelic depths for the reference and alternate allele score at each position were calculated (Takagi et al. 2013). The ΔSNP-index was calculated by subtracting the SNP-index of the resistant pool from that of the susceptible pool. Alternatively, subtracting the SNP-index of the resistant lines from that of the susceptible lines was used for ΔSNP-index analysis. To identify candidate genomic regions responsible for BPH resistance, the ΔSNP-index between susceptible pool vs. resistant pool and susceptible individuals vs. resistant individuals were calculated as absolute values. The average ΔSNP-index of all 2-Mb sliding genomic windows were calculated at 10 kb increments and plotted with a statistical confidence interval.

### LD analysis

LD analysis was performed in the BC_3_F_5_ population (KDML105 × RH) by pairwise comparisons among kompetitive allele-specific PCR(KASP)-SNP markers distributed across the two QTLs for BPH resistance identified by QTL-seq analysis using the HAPLOVIEW software version 4.2 (Barrett et al. 2005) using the following parameters: minor allele frequency (MAF) > 0.05; Hardy–Weinberg *P*-value cut-off, 0; and percentage of genotyped lines > 0.50. LD was estimated using squared allele frequency correlations (r^2^) between pairs of loci. *P*-value < 0.001 was used as a criteria for significant LD, the remaining r^2^ values were considered as uninformative. The pattern and distribution of LD were visualized and studied from LD plots generated for each chromosome using HAPLOVIEW software version 4.2. LD blocks were defined using confidence intervals (Gabriel et al. 2002).

### KASP-SNP design and single marker analysis of BILs

SNP markers from sliding windows QBPH4.1 and QBPH4.2 and previously reported BPH resistance genes were designed using 100 bp flanking sequence on either side of the polymorphisms. Two allele-specific forward primers were designed with differences at the 3′ ends where the target SNP was located, and one common reverse primer was designed following KASP genotyping technology (Additional file 4: Table S7). Single marker analysis was performed by one-way analysis of variance (ANOVA) and simple linear regression using GenStat software (18th edition) (https://www.vsni.co.uk/software/genstat/).

### Reverse screening of JHN mutant population

Screening of 9323 JHN mutant lines was conducted using an SNP in *OsLecRK3*, 21-bp indel in *OsSTPS2*, and SNP in *BPH32*. DNA was extracted using the cetyltrimethylammonium bromide (CTAB) method. Approximately 2–5 ng of DNA was genotyped using the KASP genotyping platform (KBioscience/LGC, Middlesex, UK). The M_6_ seeds of selected mutant lines were retrieved from the mutant seed bank, germinated, and tested for BPH resistance.

### Genomic sequence analysis of *OsLecRK1–3* genes

The genomic sequence of LOC_Os04g12540 (*OsLecRK1*), LOC_Os04g12560 (*OsLecRK2*), and LOC_Os04g12580 (*OsLecRK3*) of rice cultivar Nipponbare were retrieved from the Rice Genome Annotation Project (http://rice.plantbiology.msu.edu). Overlapping primers (Additional file 1: Table S9) spanning the full-length sequence of the target genes and additional 500-bp upstream and downstream sequences were designed using Primer3 version 0.4.0 (http://bioinfo.ut.ee/primer3-0.4.0/). Genomic DNA fragments of the genes were amplified using Dream*Taq* PCR Master Mix (2X) (Thermo Fisher Scientific Inc., Waltham, MA, USA). DNA fragments were purified and sequenced (Pacific Science Co., Ltd., Bangkok, Thailand). Gene sequences were assembled using CAP3 assembly (Huang and Madan 1999). Amino acid gene sequences were translated by using the ExPAsy Translate Tool (https://web.expasy.org/translate/). Nucleotide sequences of the genes from mutant and the predicted amino acid sequences of rice lines were compared using Clustal Omega (https://www.ebi.ac.uk/Tools/msa/clustalo/).

### HP mining

Nucleotide sequences of known BPH resistance genes (*BPH14*, *BPH29*, *BPH18*, *BPH26*, and *BPH9*) from different BPH resistant rice cultivars were compared with the genomic sequence of rice cultivar Nipponbare to identify SNPs. These SNP markers were used together with SNPs identified in QBPH4.1 and QBPH4.2 to generate the HP of candidate genes. HP mining was performed on each gene individually (Toivonen et al. 2000). The impact of each HP on BPH resistance was determined using a single haplotype analysis by one-way ANOVA and simple linear regression using GenStat software (18th edition) (https://www.vsni.co.uk/software/genstat/). HPs that affected BPH damage AUC significantly (*P* < 0.05) were associated with BPH resistance. The DRRs of significant HPs were calculated by dividing the mean AUC of a significant HP by the mean AUC of the JHN WT HP.

### Phylogenetic analysis

To determine whether selected mutant lines were contaminated by outcrossing, phylogenetic analysis was performed. The BPH resistant mutant lines, JHN4 and JHN09962, together with five BPH susceptible mutant lines and 15 WT-like mutant lines were genotyped by sequencing. Nine extreme BILs, RH, KDML105, Pokkali, and other germplasm were included in this analysis. Phylogenetic analysis was conducted in MEGA7 (Kumar et al. 2016). A total of 8928 RAD-derived SNPs were used as inputs in the UPGMA method at 1000 replicates bootstrap tests (Sneath and Sokal 1973). Genetic distances were computed using the p-distance method (Nei and Kumar 2000).

### Graphical genotyping

To dissect the genomic region flanking QBPH4.1 region, 84 polymorphic RAD-derived SNPs located in intergenic regions from 1.45–16.45 Mb on chromosome 4 were analyzed. 32 and 34 Thirty-two intergenic SNPs were used for graphical genotyping analysis of selected BILs while thirty-four intergenic SNPs were used for selected mutants. The KASP-SNP gene-specific markers were used within the *OsLecRK1–3*region.

## Additional files

Additional file 1: **Table S1.** Evaluations for brown planthopper (BPH) resistance of the progeny using 6 BPH populations; KPP = Kamphaeng Phet, NAN = Nan, PSL = Phitsanulok, UBN = Ubon Ratchathani, TPY = Ta Phraya, HTL = Huai Thalaeng. **Table S2.** Summary of ddRADseq data. **Table S3.** distribution on the rice chromosomes of identified SNPs in each ddRADseq library. **Table S4.** List of SNP identified on the BPH32-gene-containing sliding window (0.03–2.03 Mb) on chromosome 6. SI = SNP index; QS = QBPHS; QR = QBPHR; IndS = BPHS; IndR = BPHR; AAC = amino acid change; (−) = synonymous variant; (+) = missense variant or stop codon. DNA markers used for molecular breeding programs to improve BPH resistance were highlighted in gray. **Table S5.** Genotyping data at BPH32-SNP of selected mutant lines by reverse screening and BPH damage AUC tests using 3 BPH populations. **Table S6.** KASP-SNP marker from QBPH4.1, QBPH4.2, and BPH-R genes. **Table S8.** HP analysis of significant genes in 4 resistant mutant lines and 5 susceptible mutant lines. **Table S9.** List of overlapping primers for sequence analysis of OsLecRK1–3 genes in mutant lines. (DOCX 61 kb)

Additional file 2: **Figure S1.** The aggressiveness of three BPH populations used for BPH resistance validation of mutant lines. **Figure S2.** Average AUC of 105 BILs and their parental varieties, KDML105 and RH. The selected extreme lines for susceptible and resistance individuals were lebeled in yellow and red respectively. **Figure S3.** Aggressiveness of three BPH population used for BPH resistance validation of mutant lines. (DOCX 23 kb)

Additional file 3: Data S1. Amino acid sequence analysis of OsLecRK1 gene in JHN, identified mutants and natural BPH resistance varieties, RH. Data S2. Amino acid sequence analysis of OsLecRK2 gene in JHN, identified mutants and natural BPH resistance varieties, RH. Data S3. Amino acid sequence analysis of OsLecRK3 gene in JHN, identified mutants and natural BPH resistance varieties, RH and PTB33. (DOCX 27 kb)

Additional file 4: **Table S7.** Haplotype pattern analysis of candidate genes for BPH resistance in mutant lines. (XLSX 27 kb)
