# Supplementary material for: Identification of spontaneous mutation for broad-spectrum brown planthopper resistance in a large, long-term fast neutron mutagenized rice population
Source: Rice (N Y). 2019 Mar 19;12:16. doi: 10.1186/s12284-019-0274-1 (PMC6424995; doi:10.1186/s12284-019-0274-1)
Supplement: Supplementary file 1 — Table S1. Evaluations for brown planthopper (BPH) resistance of the progeny using 6 BPH populations; KPP = Kamphaeng Phet, NAN = Nan, PSL = Phitsanulok, UBN = Ubon Ratchathani, TPY = Ta Phraya, HTL = Huai Thalaeng. Table S2. Summary of ddRADseq data. Table S3. distribution on the rice chromosomes of identified SNPs in each ddRADseq library. Table S4. List of SNP identified on the BPH32-gene-containing sliding window (0.03–2.03 Mb) on chromosome 6. SI = SNP index; QS = QBPHS; QR = QBPHR; IndS = BPHS; IndR = BPHR; AAC = amino acid change; (−) = synonymous variant; (+) = missense variant or stop codon. DNA markers used for molecular breeding programs to improve BPH resistance were highlighted in gray. Table S5. Genotyping data at BPH32-SNP of selected mutant lines by reverse screening and BPH damage AUC tests using 3 BPH populations. Table S6. KASP-SNP marker from QBPH4.1, QBPH4.2, and BPH-R genes. Table S8. HP analysis of significant genes in 4 resistant mutant lines and 5 susceptible mutant lines. Table S9. List of overlapping primers for sequence analysis of OsLecRK1–3 genes in mutant lines. (DOCX 61 kb) [file 12284_2019_274_MOESM1_ESM.docx]

Additional File 1

Identification of Spontaneous Mutation for Broad-Spectrum Brown Planthopper Resistance in a Large, Long-Term Fast Neutron Mutagenized Rice Population

Wintai Kamolsukyeunyong^1^, Siriphat Ruengphayak^2^, Pantharika Chumwong^1^, Ekawat Chaichumpoo^2^, Watchareewan Jamboonsri^1^, Chatree Saensuk^2^, Kunyakarn Phoonsiri^2^, Theerayut Toojinda^1, 3^ and Apichart Vanavichit^1, 2, 4^*

*** Correspondence:** Dr. ApichartVanavichit: vanavichit@gmail.com

**Tables**

**Table S1** Evaluations for brown planthopper (BPH) resistance of the progeny using 6 BPH populations; KPP = KamphaengPhet, NAN = Nan, PSL = Phitsanulok, UBN = UbonRatchathani, TPY = Ta Phraya, HTL = HuaiThalaeng

| **Entry** | **Code** | **Code WS** | **KPP** | **NAN** | **PSL** | **UBN** | **TPY** | **HTL** | **Average** |
| --- | --- | --- | --- | --- | --- | --- | --- | --- | --- |
| 1 | UBN03078-80-354-11 | WS13-S13-1 | 23.3 | 30.0 | 35.0 | 26.3 | 21.7 | 33.7 | 28.3 |
| 2 | UBN03078-80-354-20 | WS13-S13-2 | 18.3 | 25.3 | 23.0 | 23.0 | 14.3 | 33.0 | 22.8 |
| 3 | UBN03078-81-504-1 | WS13-S13-3 | 25.3 | 35.7 | 36.0 | 32.7 | 26.7 | 34.3 | 31.8 |
| 4 | UBN03078-80-354-7 | WS13-S13-4 | 35.7 | 30.3 | 32.0 | 32.0 | 22.0 | 35.7 | 31.3 |
| 5 | UBN03078-80-354-7 | WS13-S13-5 | 29.7 | 31.0 | 31.7 | 35.7 | 30.0 | 31.3 | 31.6 |
| 6 | UBN03078-80-354-11 | WS13-S13-6 | 15.0 | 21.3 | 19.7 | 24.0 | 25.0 | 34.0 | 23.2 |
| 7 | UBN03078-80-354-12 | WS13-S13-7 | 25.0 | 20.3 | 21.0 | 31.3 | 25.7 | 33.0 | 26.1 |
| 8 | UBN03078-80-354-15 | WS13-S13-8 | 15.7 | 19.3 | 24.0 | 21.3 | 21.7 | 19.3 | 20.2 |
| 9 | UBN03078-80-354-16 | WS13-S13-9 | 25.7 | 25.3 | 26.0 | 23.7 | 20.3 | 29.0 | 25.0 |
| 10 | UBN03078-101-342-4-19 | WS13-S13-10 | 19.7 | 34.7 | 29.3 | 25.0 | 23.7 | 18.0 | 25.1 |
| 11 | UBN03078-101-342-4-20 | WS13-S13-11 | 12.0 | 23.0 | 12.3 | 12.0 | 14.3 | 14.3 | 14.7 |
| 12 | UBN03078-101-342-4-32 | WS13-S13-12 | 13.0 | 28.3 | 22.3 | 15.0 | 15.7 | 25.7 | 20.0 |
| 13 | UBN03078-101-342-4-96 | WS13-S13-13 | 13.7 | 22.0 | 23.0 | 12.3 | 17.7 | 16.0 | 17.4 |
| 14 | UBN03078-101-342-4-106 | WS13-S13-14 | 11.0 | 27.0 | 19.0 | 12.7 | 14.3 | 21.7 | 17.6 |
| 15 | UBN03078-101-342-4-111 | WS13-S13-15 | 12.0 | 19.7 | 13.0 | 13.0 | 23.3 | 15.0 | 16.0 |
| 16 | UBN03078-101-342-4-114 | WS13-S13-16 | 13.7 | 32.7 | 23.7 | 31.7 | 18.7 | 27.3 | 24.6 |
| 17 | UBN03078-101-342-4-141 | WS13-S13-17 | 16.0 | 34.0 | 28.7 | 25.3 | 18.7 | 19.7 | 23.7 |
| 18 | UBN03078-101-342-4-143 | WS13-S13-18 | 12.0 | 25.3 | 17.0 | 17.0 | 23.0 | 19.7 | 19.0 |
| 19 | UBN03078-101-342-4-144 | WS13-S13-19 | 14.7 | 28.0 | 20.7 | 14.3 | 14.3 | 15.7 | 17.9 |
| 20 | UBN03078-101-342-4-147 | WS13-S13-20 | 12.0 | 12.0 | 14.3 | 9.3 | 14.7 | 21.0 | 13.9 |
| 21 | UBN03078-101-342-4-148 | WS13-S13-21 | 17.0 | 35.3 | 30.7 | 29.7 | 23.7 | 30.7 | 27.8 |
| 22 | UBN03078-101-342-6-49 | WS13-S13-22 | 12.0 | 25.3 | 12.3 | 11.0 | 13.3 | 21.7 | 15.9 |
| 23 | UBN03078-101-342-6-58 | WS13-S13-24 | 11.7 | 21.3 | 23.0 | 14.7 | 15.0 | 16.0 | 16.9 |
| 24 | UBN03078-101-342-6-82 | WS13-S13-25 | 14.3 | 30.0 | 32.3 | 17.3 | 18.7 | 33.0 | 24.3 |
| 25 | UBN03078-101-342-6-89 | WS13-S13-26 | 12.0 | 23.7 | 21.3 | 13.0 | 22.0 | 21.7 | 18.9 |
| 26 | UBN03078-101-450-2 | WS13-S13-27 | 20.7 | 29.7 | 19.0 | 20.0 | 21.0 | 26.7 | 22.8 |
| 27 | UBN03078-80-28-1 | WS13-S13-28 | 15.3 | 28.3 | 21.7 | 21.7 | 15.7 | 28.0 | 21.8 |
| 28 | UBN03078-80-28-5 | WS13-S13-29 | 26.7 | 31.3 | 31.7 | 26.7 | 27.0 | 29.0 | 28.7 |
| 29 | UBN03078-101-342-16 | WS13-S13-30 | 14.7 | 31.3 | 31.0 | 19.7 | 23.3 | 23.7 | 23.9 |
| 30 | UBN03078-101-342-4-16 | WS13-S13-31 | 12.0 | 26.0 | 23.0 | 15.0 | 16.3 | 17.3 | 18.3 |
| 31 | UBN03078-101-342-4-24 | WS13-S13-32 | 12.0 | 15.3 | 16.7 | 12.0 | 14.7 | 19.3 | 15.0 |
| 32 | UBN03078-101-342-4-97 | WS13-S13-33 | 13.0 | 33.3 | 27.0 | 21.0 | 21.7 | 24.0 | 23.3 |
| 33 | UBN03078-101-342-4-126 | WS13-S13-34 | 14.7 | 29.0 | 26.0 | 21.0 | 19.3 | 15.3 | 20.9 |
| 34 | UBN03078-101-342-4-135 | WS13-S13-35 | 12.0 | 30.7 | 21.3 | 14.7 | 18.3 | 16.0 | 18.8 |
| 35 | UBN03078-101-342-4-158 | WS13-S13-36 | 13.0 | 22.7 | 21.7 | 20.3 | 17.3 | 15.3 | 18.4 |
| 36 | UBN03078-80-28-5 | WS13-S13-37 | 19.0 | 36.0 | 36.0 | 29.0 | 25.3 | 28.0 | 28.9 |
| 37 | UBN03078-80-28-5 | WS13-S13-38 | 15.7 | 32.7 | 24.3 | 17.3 | 16.7 | 13.7 | 20.1 |
| 38 | UBN03078-101-342-9 | WS13-S13-39 | 14.3 | 17.3 | 12.3 | 16.7 | 15.7 | 21.7 | 16.3 |
| 39 | UBN03078-101-342-4-134 | WS13-S13-41 | 14.7 | 34.0 | 27.3 | 20.3 | 22.0 | 14.3 | 22.1 |
| 40 | UBN03078-101-342-2 | WS13-S13-42 | 15.0 | 28.7 | 25.3 | 20.0 | 25.3 | 16.0 | 21.7 |
| 41 | UBN03078-101-450-1 | WS13-S13-43 | 25.7 | 33.7 | 27.3 | 28.7 | 29.0 | 33.0 | 29.6 |
| 42 | UBN03078-101-342-11 | WS13-S13-44 | 16.7 | 35.7 | 30.7 | 25.7 | 32.3 | 24.7 | 27.6 |
| 43 | UBN03078-101-450-2 | WS13-S13-45 | 12.0 | 23.7 | 19.7 | 14.7 | 14.0 | 10.7 | 15.8 |
| 44 | UBN03078-101-342-4-138 | WS13-S13-46 | 12.3 | 21.0 | 15.3 | 15.7 | 15.3 | 10.0 | 14.9 |
| 45 | UBN03078-101-450-1 | WS13-S13-47 | 14.7 | 22.7 | 20.7 | 22.0 | 17.3 | 21.3 | 19.8 |
| 46 | UBN03078-101-60-20 | WS13-S13-48 | 12.7 | 27.3 | 27.0 | 11.3 | 14.7 | 4.7 | 16.3 |
| 47 | UBN03078-80-28-1 | WS13-S13-49 | 21.0 | 23.3 | 28.7 | 25.3 | 27.3 | 32.3 | 26.3 |
| 48 | UBN03078-80-354-20 | WS13-S13-50 | 14.0 | 24.7 | 25.3 | 23.3 | 21.7 | 19.3 | 21.4 |
| 49 | 29(3) | WS10-SM8-1 | 12.0 | 28.0 | 16.0 | 14.3 | 22.0 | 19.3 | 18.6 |
| 50 | 82(4) | WS10-SM8-2 | 12.0 | 26.0 | 20.0 | 14.0 | 13.3 | 25.0 | 18.4 |
| 51 | 140(4) | WS10-SM8-3 | 16.7 | 35.0 | 28.3 | 20.0 | 22.3 | 18.0 | 23.4 |
| 52 | 158(3) | WS10-SM8-4 | 14.3 | 27.0 | 24.7 | 14.7 | 17.0 | 14.3 | 18.7 |
| 53 | 163(4) | WS10-SM8-5 | 13.7 | 31.7 | 24.7 | 17.3 | 21.7 | 15.7 | 20.8 |
| 54 | 164(4) | WS10-SM8-6 | 12.0 | 28.0 | 14.7 | 13.3 | 16.7 | 14.0 | 16.4 |
| 55 | 170(4) | WS10-SM8-7 | 11.0 | 26.3 | 19.3 | 16.3 | 25.3 | 21.0 | 19.9 |
| 56 | 229(3) | WS10-SM8-8 | 12.0 | 36.0 | 26.3 | 14.7 | 24.3 | 18.7 | 22.0 |
| 57 | 237(4) | WS10-SM8-9 | 12.0 | 21.3 | 14.0 | 10.3 | 19.7 | 23.0 | 16.7 |
| 58 | 282(3) | WS10-SM8-10 | 12.0 | 18.7 | 17.0 | 12.7 | 16.7 | 14.3 | 15.2 |
| 59 | 318(4) | WS10-SM8-12 | 12.0 | 31.3 | 23.0 | 14.7 | 19.0 | 17.3 | 19.6 |
| 60 | 327(4) | WS10-SM8-14 | 12.0 | 22.7 | 12.3 | 12.0 | 16.7 | 16.7 | 15.4 |
| 61 | 329(4) | WS10-SM8-15 | 12.0 | 26.7 | 18.7 | 11.7 | 16.7 | 30.0 | 19.3 |
| 62 | 338(4) | WS10-SM8-16 | 12.0 | 32.3 | 22.7 | 14.0 | 17.0 | 18.3 | 19.4 |
| 63 | 393(4) | WS10-SM8-17 | 12.0 | 28.3 | 15.7 | 15.7 | 17.3 | 20.3 | 18.2 |
| 64 | 410(4) | WS10-SM8-18 | 12.0 | 33.7 | 23.0 | 17.0 | 18.7 | 18.0 | 20.4 |
| 65 | 423(4) | WS10-SM8-19 | 11.7 | 16.7 | 13.7 | 11.0 | 16.0 | 18.3 | 14.6 |
| 66 | 437/2/(4) | WS10-SM8-20 | 15.0 | 27.7 | 19.0 | 19.7 | 21.3 | 28.0 | 21.8 |
| 67 | 1(5) | WS10-SM8-21 | 15.0 | 24.7 | 21.7 | 14.7 | 18.3 | 25.7 | 20.0 |
| 68 | 9(7) | WS10-SM8-22 | 18.0 | 34.7 | 28.3 | 25.3 | 26.7 | 23.0 | 26.0 |
| 69 | 13(5) | WS10-SM8-23 | 14.7 | 34.3 | 21.0 | 15.7 | 18.7 | 25.0 | 21.6 |
| 70 | 42(7) | WS10-SM8-24 | 12.0 | 22.3 | 16.0 | 19.7 | 26.7 | 29.7 | 21.1 |
| 71 | 78(7) | WS10-SM8-25 | 12.7 | 32.7 | 18.3 | 16.7 | 17.7 | 27.7 | 20.9 |
| 72 | 81(7) | WS10-SM8-26 | 13.7 | 34.0 | 26.0 | 21.0 | 17.0 | 22.0 | 22.3 |
| 73 | 114(7) | WS10-SM8-27 | 13.7 | 33.7 | 25.3 | 20.3 | 17.0 | 21.0 | 21.8 |
| 74 | 125(5) | WS10-SM8-28 | 12.0 | 30.0 | 18.0 | 14.7 | 20.7 | 16.7 | 18.7 |
| 75 | 133(8) | WS10-SM8-29 | 14.7 | 34.7 | 26.0 | 25.0 | 24.7 | 25.0 | 25.0 |
| 76 | 141(7) | WS10-SM8-30 | 12.0 | 35.0 | 31.3 | 17.7 | 24.3 | 21.7 | 23.7 |
| 77 | 142(5) | WS10-SM8-31 | 15.0 | 27.7 | 19.7 | 21.0 | 22.0 | 20.7 | 21.0 |
| 78 | 144(7) | WS10-SM8-33 | 14.7 | 36.0 | 23.3 | 24.7 | 21.7 | 21.7 | 23.7 |
| 79 | 146(5) | WS10-SM8-34 | 12.0 | 35.0 | 15.0 | 14.7 | 20.0 | 27.3 | 20.7 |
| 80 | 148(5) | WS10-SM8-35 | 12.0 | 32.0 | 18.7 | 16.7 | 23.7 | 21.3 | 20.7 |
| 81 | 155(5) | WS10-SM8-37 | 14.7 | 32.3 | 32.0 | 20.7 | 25.3 | 22.0 | 24.5 |
| 82 | 161(5) | WS10-SM8-38 | 13.0 | 24.0 | 22.0 | 20.0 | 17.7 | 24.7 | 20.2 |
| 83 | 169(7) | WS10-SM8-39 | 14.3 | 22.0 | 21.7 | 13.0 | 23.0 | 25.7 | 19.9 |
| 84 | 179(5) | WS10-SM8-40 | 14.3 | 31.7 | 23.7 | 20.0 | 23.3 | 17.7 | 21.8 |
| 85 | 180(5) | WS10-SM8-41 | 12.0 | 33.7 | 17.3 | 16.0 | 24.0 | 22.0 | 20.8 |
| 86 | 192(5) | WS10-SM8-42 | 13.0 | 28.0 | 24.3 | 18.0 | 20.0 | 25.3 | 21.4 |
| 87 | 193(5) | WS10-SM8-43 | 12.0 | 31.3 | 17.7 | 12.3 | 19.3 | 24.0 | 19.4 |
| 88 | 194(5) | WS10-SM8-44 | 15.7 | 28.3 | 21.7 | 18.3 | 21.3 | 28.3 | 22.3 |
| 89 | 213(6) | WS10-SM8-45 | 14.7 | 35.3 | 21.0 | 18.7 | 18.3 | 20.0 | 21.3 |
| 90 | 228(6) | WS10-SM8-46 | 12.3 | 28.7 | 18.7 | 17.0 | 15.3 | 16.7 | 18.1 |
| 91 | 249(7) | WS10-SM8-48 | 16.0 | 30.0 | 21.7 | 15.0 | 20.3 | 20.3 | 20.6 |
| 92 | 251(7) | WS10-SM8-49 | 12.7 | 26.0 | 22.3 | 23.3 | 20.3 | 26.0 | 21.8 |
| 93 | 257(7) | WS10-SM8-50 | 13.0 | 23.7 | 16.3 | 17.0 | 14.3 | 19.7 | 17.3 |
| 94 | 258(7) | WS10-SM8-51 | 12.0 | 33.7 | 24.7 | 19.3 | 20.7 | 24.3 | 22.4 |
| 95 | 269(7) | WS10-SM8-52 | 16.7 | 28.3 | 21.3 | 22.7 | 23.0 | 20.7 | 22.1 |
| 96 | 286(5) | WS10-SM8-53 | 12.0 | 20.7 | 13.0 | 14.0 | 18.0 | 24.0 | 16.9 |
| 97 | 306(5) | WS10-SM8-55 | 12.0 | 21.0 | 17.3 | 15.7 | 21.7 | 21.3 | 18.2 |
| 98 | 315(5) | WS10-SM8-56 | 19.7 | 29.0 | 27.0 | 18.3 | 20.0 | 25.0 | 23.2 |
| 99 | 323(5) | WS10-SM8-57 | 15.0 | 36.0 | 29.0 | 19.0 | 22.7 | 16.3 | 23.0 |
| 100 | 330(7) | WS10-SM8-59 | 12.3 | 24.7 | 20.7 | 15.0 | 19.3 | 12.7 | 17.4 |
| 101 | 332(7) | WS10-SM8-60 | 12.0 | 29.3 | 18.3 | 17.3 | 17.3 | 26.7 | 20.2 |
| 102 | 333(5) | WS10-SM8-61 | 12.0 | 35.3 | 21.7 | 22.7 | 20.0 | 18.0 | 21.6 |
| 103 | 345(7) | WS10-SM8-62 | 13.3 | 33.7 | 20.7 | 18.7 | 24.3 | 24.3 | 22.5 |
| 104 | 349(7) | WS10-SM8-63 | 12.0 | 35.7 | 31.7 | 28.3 | 24.3 | 20.0 | 25.3 |
| 105 | 428(7) | WS10-SM8-64 | 14.3 | 30.7 | 27.7 | 19.7 | 25.7 | 21.3 | 23.2 |
| - | KD |  | 34.0 | 36.0 | 36.0 | 35.3 | 30.3 | 36.0 | 34.6 |
| - | Rathu |  | 12.3 | 17.3 | 10.3 | 9.7 | 9.7 | 8.3 | 11.3 |

**Table S2** Summary of ddRAD data

| ddRAD | number of lines | Read | mean read length | total read length | % reference genome covered | genome covered | sequence depth (x) |
| --- | --- | --- | --- | --- | --- | --- | --- |
| BPHS1 | 1 | 3,567,944 | 98 | 349,338,262 | 6 | 22,394,731 | 16 |
| BPHS2 | 1 | 2,650,493 | 100 | 266,215,580 | 6 | 22,394,731 | 12 |
| BPHS3 | 1 | 3,926,772 | 100 | 392,780,494 | 6 | 22,394,731 | 18 |
| BPHS4 | 1 | 4,720,123 | 99 | 469,511,780 | 7 | 26,127,186 | 18 |
| BPHR1 | 1 | 4,414,647 | 97 | 430,280,866 | 6 | 22,394,731 | 19 |
| BPHR2 | 1 | 1,979,334 | 96 | 189,796,400 | 5 | 18,662,276 | 10 |
| BPHR3 | 1 | 1,698,324 | 99 | 167,770,189 | 4 | 14,929,821 | 11 |
| BPHR4 | 1 | 3,430,300 | 98 | 337,021,050 | 6 | 22,394,731 | 15 |
| BPHR5 | 1 | 3,038,004 | 98 | 297,004,737 | 6 | 22,394,731 | 13 |
| QBPHR | 19 | 4,325,303 | 79 | 339,894,574 | 2 | 7,464,910 | 46 |
| QBPHS | 16 | 2,784,445 | 80 | 223,743,742 | 2 | 7,464,910 | 30 |

**Table S3** distribution on the rice chromosomes of identified SNPs in each ddRAD library.

|  | Counted SNP | | | | | | | | | | | |
| --- | --- | --- | --- | --- | --- | --- | --- | --- | --- | --- | --- | --- |
|  | Chr1 | Chr2 | Chr3 | Chr4 | Chr5 | Chr6 | Chr7 | Chr8 | Chr9 | Chr10 | Chr11 | Chr12 |
| BPHS1 | 812 | 758 | 635 | 578 | 475 | 473 | 553 | 381 | 321 | 370 | 538 | 384 |
| BPHS2 | 770 | 597 | 521 | 501 | 354 | 491 | 461 | 291 | 291 | 361 | 409 | 326 |
| BPHS3 | 1139 | 989 | 836 | 848 | 618 | 675 | 712 | 542 | 453 | 510 | 749 | 547 |
| BPHS4 | 1447 | 1170 | 938 | 866 | 674 | 721 | 830 | 646 | 512 | 649 | 787 | 563 |
| BPHR1 | 1145 | 933 | 770 | 763 | 546 | 610 | 676 | 485 | 457 | 471 | 659 | 487 |
| BPHR2 | 380 | 329 | 256 | 267 | 173 | 190 | 203 | 171 | 121 | 131 | 197 | 165 |
| BPHR3 | 359 | 288 | 258 | 289 | 165 | 232 | 194 | 149 | 143 | 148 | 203 | 162 |
| BPHR4 | 827 | 700 | 593 | 598 | 418 | 485 | 469 | 352 | 346 | 375 | 559 | 396 |
| BPHR5 | 811 | 630 | 549 | 562 | 404 | 436 | 455 | 306 | 295 | 349 | 532 | 324 |
| QBPHR1 | 251 | 222 | 210 | 235 | 128 | 137 | 173 | 89 | 117 | 107 | 159 | 137 |
| QBPHS1 | 211 | 176 | 177 | 177 | 109 | 135 | 130 | 71 | 111 | 98 | 136 | 112 |

**Table S4** List of SNP identified on the BPH 32-gene-containing sliding window (0.03 – 2.03 Mb) on chromosome 6. SI = SNP index; QS = QBPHS; QR = QBPHR; IndS = BPHS; IndR = BPHR; AAC = amino acid change; (-) = synonymous variant; (+) = missense variant or stop codon. DNA markers used for molecular breeding programs to improve BPH resistance were highlighted in gray.

| **Position** | **LOC identifier** | **gene product name** | **SNP** | **SI in QS** | **SI in QR** | **SI in InS** | **SI in InR** | **SNP pos / CDS length** | **SNP effect** | **AAC** | **AAC position/protein length** |
| --- | --- | --- | --- | --- | --- | --- | --- | --- | --- | --- | --- |
| 197010 | LOC_Os06g01320 | chromo domain containing protein | C/T | 35.71 | 79.49 | 35.71 | 79.49 | SNP in intron | - |  |  |
| 380568 | LOC_Os06g01640 | arginine N-methyltransferase 7 | G/A | 61.54 | 9.09 | 61.54 | 10.00 | SNP in intron | - |  |  |
| 777658 | LOC_Os06g02330 | expressed protein | C/T | NA | NA | 41.67 | 0.00 | 1186/1622 | + | D>N | 242/271 |
| 895398 | LOC_Os06g02560 | growth-regulating factor | G/A | 41.67 | 0.00 | 41.67 | 0.00 | 521/1703 | - |  |  |
| 1380866 | RM589 | SSR marker linked to Bph32 |  |  |  |  |  |  |  |  |  |
| 1426863 | LOC_Os06g03660 | peroxisomal biogenesis factor 11 | C/T | 56.92 | 0.00 | 56.92 | 0.00 | 1110/1823 | - |  |  |
| 1446275 | LOC_Os06g03682 | calcium-dependent protein kinase isoform AK1 | G/A | 40.00 | 4.90 | 42.86 | 1.27 | 1324/2092 | + | A>V | 352/506 |
| 1476793 | RM586 | SSR marker linked to Bph32 |  |  |  |  |  |  |  |  |  |
| 1530948 | LOC_Os06g03820 | expressed protein | A/G | NA | NA | 37.50 | 0.00 | 839/1577 | + | S>G | 254/393 |
| 1545234 | LOC_Os06g03840 | H-BTB4 - Bric-a-Brac, Tramtrack, Broad Complex BTB domain with H family conserved sequence | G/C | NA | NA | 23.81 | 100.00 | 657/1847 | - |  |  |
| 1545246 | LOC_Os06g03840 | H-BTB4 - Bric-a-Brac, Tramtrack, Broad Complex BTB domain with H family conserved sequence | C/T | 51.43 | 100.00 | 51.43 | 100.00 | 645/1847 | - |  |  |
| 1545250 | LOC_Os06g03840 | H-BTB4 - Bric-a-Brac, Tramtrack, Broad Complex BTB domain with H family conserved sequence | C/T | 50.00 | 100.00 | 50.00 | 100.00 | 641/1847 | + | R>Q | 104/433 |
| 1545370 | LOC_Os06g03840 | H-BTB4 - Bric-a-Brac, Tramtrack, Broad Complex BTB domain with H family conserved sequence | C/T | 50.48 | 90.91 | 52.70 | 88.46 | 521/1847 | + | G>E | 64/433 |
| 1562619 | SSR25 | SSR marker linked to Bph32 |  |  |  |  |  |  |  |  |  |
| 1592445 | LOC_Os06g03940 | spastin | C/G | 70.00 | 0.00 | 70.00 | 0.00 | SNP in intron | - |  |  |
| 1595702 | LOC_Os06g03940 | spastin | A/G | 57.69 | 4.35 | 57.69 | 0.00 | 1477/1934 | + | E>G | 456/487 |
| 1611398 | RM588 | SSR linked to Bph32 |  |  |  |  |  |  |  |  |  |
| 1637636 | LOC_Os06g04010 | GAGA-binding protein | A/G | 60.53 | 0.00 | 60.53 | 0.00 | 508/1620 | - |  |  |
| 1669353 | LOC_Os06g04060 | expressed protein | A/C | 37.50 | 0.00 | 37.50 | 0.00 | 227/495 | + | E>A | 76/164 |
| 1675574 | LOC_Os06g04070 | pyridoxal-dependent decarboxylase protein | A/C | 45.00 | 8.33 | 64.71 | 0.00 | 1833/3362 | - |  |  |
| 1675659 | LOC_Os06g04070 | pyridoxal-dependent decarboxylase protein | G/A | 69.57 | 10.71 | 69.57 | 10.71 | 1748/3362 | + | A>V | 372/702 |
| 1692963 | LOC_Os06g04080 | glycosyl hydrolases family 17 | A/G | 57.14 | 0.00 | 57.14 | 0.00 | 1079/1716 | - |  |  |
| 1693774 | LOC_Os06g04080 | glycosyl hydrolases family 17 | T/G | 52.94 | 0.00 | 52.94 | 0.00 | 1350/1716 | + | S>A | 443/449 |

**Table S5** Genotyping data at BPH32-SNP of selected mutant lines by reverse screening and BPH damage AUC tests using 3 BPH populations.

| JHN no. | Bph32_2_1223332 | BPH damage AUC | | |
| --- | --- | --- | --- | --- |
|  |  | CNT | TPY | UBN |
| JHN14361 | G:G |  |  |  |
| JHN18922 | G:G |  |  |  |
| JHN19466 | G:G | 63 | 63 | 63 |
| JHN19524 | G:G | 63 | 63 | 63 |
| JHN19601 | G:G |  |  |  |
| JHN19619 | G:G |  |  |  |
| JHN19647 | G:G |  |  |  |
| JHN21425 | G:G | 62 | 63 | 63 |
| JHN21595 | G:G | 63 | 63 | 63 |
| JHN21692 | G:G |  |  |  |
| JHN22060 | G:G |  |  |  |
| JHN23040 | G:G |  |  |  |
| JHN23122 | G:G |  |  |  |
| JHN23234 | G:G |  |  |  |
| JHN23370 | G:G |  |  |  |
| JHN00332 | G:G | 63 | 63 | 63 |
| TN1 | G:G | 63 | 63 | 63 |
| JHN | C:C | 63 | 63 | 63 |
| RH | G:G | 21.67 | 14.33 | 8.33 |

**Table S6** KASP-SNP marker from QBPH4.1, QBPH4.2, and BPH-R genes

| KASP marker name | FAM Allele | HEX Allele | Aliquot ID^a^ |
| --- | --- | --- | --- |
| BPH14_Chr3_35698065 | C | A | 236766037 |
| BPH14_Chr3_35697944 | A | G | 236766038 |
| R04005867219 | C | G | 239933038 |
| R04006069830 | G | T | 0239932267 |
| R04006069877 | A | G | 0239932268 |
| R04006069900 | A | G | 0239932269 |
| R04006263057 | G | C | 0239932270 |
| R04006263087 | A | T | 0239932271 |
| R04006263168 | C | G | 0239932272 |
| R04006388017 | C | A | 0239932273 |
| R04006388095 | G | C | 0239932274 |
| R04006451404 | T | C | 0239932275 |
| R04006451424 | C | A | 0239932277 |
| R04006451469 | G | A | 0239932238 |
| R04006451485 | A | G | 0239932279 |
| R04006451495 | T | G | 0239932280 |
| R04006659844 | C | A | 0239932281 |
| R04006659845 | C | G | 0239932282 |
| R04006659872 | C | T | 0239932283 |
| R04006659873 | A | T | 0239932284 |
| R04006659884 | G | C | 0239932285 |
| R04006659915 | T | C | 0239932286 |
| R04006925203 | T | G | 0239932287 |
| LecRK1_aac289 | G | T | 1162033478 |
| LecRK1_aac246 | G | A | 1162033477 |
| LecRK1_aac129 | C | T | 1162033476 |
| LecRK1_aac122 | CA | AG | 1162033475 |
| LecRK1_aac90 | G | A | 1162033474 |
| LecRK1_aac35 | A | G | 1162028619 |
| LecRK2_aac758 | C | G | 1162033482 |
| LecRK2_SNP8_aac534 | G | A | 1145074633 |
| LecRK2_SNP7_aac495 | T | C | 1145074612 |
| LecRK2_aac424 | G | A | 1162033481 |
| LecRK2_aac151 | T | C | 1162033480 |
| LecRK3_Chr4_6967158_aac627 | TT | GG | 0221537212 |
| OsLecRK3-SNP | A | G | 1126716796. 1132528804. 1140749254 |
| LecRK3_Chr4_6968472_aac189 | T | C | 0221537152 |
| LecRK3_Chr4_6968762_aac92 | G | A | 0221537215 |
| LecRK3_Chr4_6968772_aac89 | G | A | 0221537125 |
| LecRK3_Chr4_6968776_aac88 | A | C | 0221537165 |
| LecRK3_Chr4_6968816_aac74 | G | C | 0221537189 |
| LecRK3_Chr4_6969215_5’utr | G | A | 0221537177 |
| R04015916138 | A | C | 239932288 |
| OsSTPS2_SNP1 | G | A | 1114308048 |
| OsSTPS2_SNP2 | T | G | 1114308033. 1103441200. 1114308033_ 1103441200 |
| OsSTPS2_SNP3 | CA | TG | 1114308024 |
| OsSTPS2_21bp_del | TTTATGCCTCTGGTGTGACCA | - | 1122144750 |
| R04016380993 | A | C | 0239932289 |
| R04016627505 | C | G | 0239932290 |
| R04016672214 | T | G | 0239932291 |
| BPH29_Chr6_484454 | - | GGCGGCGGCGGC | 236766036 |
| BPH29_Chr6_484507 | C | T | 236766035 |
| Bph32_2_1223332 | C | G | 1140710816 |
| BPH18_Chr12_22868930 | T | C | 236766044 |
| BPH18_Chr12_22869061 | A | G | 236766043 |
| BPH18_Chr12_22885393 | T | A | 236766042 |
| BPH26_Chr12_22875096 | T | A | 236766040 |
| BPH26_Chr12_22885905 | G | A | 236766039 |
| BPH9_Chr12_22886051 | G | C | 236766033 |
| BPH9_Chr12_22886067 | A | G | 236766032 |
| BPH9_Chr12_22886105 | G | T | 236766034 |

^a^LGCgroup (2015). KASP genotyping technology. <https://www.lgcgroup.com/products/kasp-genotyping-chemistry/#.Wrhk3dR94_4>

**Table S8** HP analysis of significant genes in 4 resistance mutant lines and 5 susceptible mutant lines. Number 1 to 4 represented HP number of each gene by which HP number 1 was specific to WT (Additional file 4: Table S6). The yellow color was highlighted for the genes which HPs similar to those HPs found in 4 resistance mutant lines and the green color was highlighted for the genes which HPs differ from those found in 4 resistance mutant lines. R = resistance, MR = moderately resistance, S = susceptible

The five susceptible mutant lines including JHN07766, JHN05678, JHN19572, JHN19577, and JHN19578 showed almost similar HPs at significant genes on chromosome 4 and *BPH32* gene on chromosome 6 as those four resistance mutant lines except *BPH9* gene on chromosome 12.

| KASP marker name | inorganic phosphate transporter | gamma thionin protein | F-box 118 | F-box 119 | LR10 | OsLecRK1 | OsLecRK2 | OsLecRK3 | OsSTPS2 | Verticillium wilt resistance protein | BPH32 | BPH9 | BPH -CNT | BPH -TPY | BPH- UBN |
| --- | --- | --- | --- | --- | --- | --- | --- | --- | --- | --- | --- | --- | --- | --- | --- |
| RH | 1 | 2 | 3 | 3 | 2 | 2 | 3 | 2 | 2 | 1 | 2 | 1 | R | R | R |
| JHN | 1 | 1 | 1 | 1 | 1 | 1 | 1 | 1 | 1 | 1 | 1 | 1 | S | S | S |
| JHN4 | 1 | 2 | 2 | 2 | 2 | 2 | 2 | 2 | 2 | 2 | 1 | 2 | R | R | R |
| JHN12005 | 1 | 2 | 3 | 3 | 2 | 3 | 3 | 2 | 2 | 2 | 1 | 3 | R | R | MR |
| JHN19525 | 1 | 2 | 2 | 3 | 2 | 2 | 3 | 2 | 2 | 2 | 1 | 2 | MR | R | MR |
| JHN09962 | 1 | 2 | 3 | 3 | 2 | 2 | 2 | 2 | 2 | 2 | 1 | 2 | S | R | R |
| JHN07766 | 1 | 2 | 2 | 4 | 2 | 3 | 4 | 2 | 2 | 2 | 1 | 1 | S | S | S |
| JHN05678 | 1 | 2 | 3 | 3 | 2 | 2 | 2 | 2 | 2 | 2 | 1 | 1 | S | S | S |
| JHN19572 | 1 | 2 | 2 | 3 | 2 | 2 | 3 | 2 | 2 | 2 | 1 | 1 | S | S | S |
| JHN19577 | 1 | 3 | 2 | 4 | 2 | 2 | 3 | 2 | 2 | 2 | 1 | 1 | S | S | S |
| JHN19578 | 1 | 2 | 3 | 2 | 2 | 2 | 3 | 2 | 2 | 2 | 1 | 1 | S | S | S |

**Table S9** List of overlapping primers for sequence analysis of OsLecRK1-3 genes in mutant lines

| name | forward (5'--> 3') | reverse (5' --> 3') |
| --- | --- | --- |
| Os04g12540_1 | ttcagagacacatgtcacacatt | gctagcatatgccacactgg |
| Os04g12540_2 | gccatctgattcttttctcca | agtcagacagtggccaatcc |
| Os04g12540_3 | atgcccaccatggtacaagt | caagcgcgatatgaactcg |
| Os04g12540_4 | atgaaggagccgaaagactg | agctcaaagagatggaccagtt |
| Os04g12540_5 | tgcgtcaaaacaataagcattt | tagcaagccggataccttcc |
| Os04g12540_6 | tcgtgctactggcttcctgt | tcaggttctcaggttgcaga |
| Os04g12560_1 | acgatcagcaaagcaggttc | tagcccacatcagtgactcg |
| Os04g12560_2 | accgatacaagtccaagctg | cggcattcggtctcatctat |
| Os04g12560_3 | gttgcaggccagattttgag | cgtgaaaccccaagtgctac |
| Os04g12560_4 | tgcttggtttttgcaatgag | tgcttctatcatgcgagtgaa |
| Os04g12560_5 | gttgctgtggcactgtggt | tggggttttgaactatttgg |
| Os04g12580_1 | ttggaggtctatatgtttcatcca | acggtcttgtcagcgatctt |
| Os04g12580_2 | atctcgccctctgctgatt | atgctccactacccaccatt |
| Os04g12580_3 | acatgccaggcccttatg | ttgcagctgtcctttgtaga |
| Os04g12580_4 | cctccaaagattttcacttacagc | accacagtgccacagcaac |
| Os04g12580_5 | ttgggcaaatgattgctaca | gcatctaactgaatggccaac |
