# Supplementary material for: Identification of spontaneous mutation for broad-spectrum brown planthopper resistance in a large, long-term fast neutron mutagenized rice population
Source: Rice (N Y). 2019 Mar 19;12:16. doi: 10.1186/s12284-019-0274-1 (PMC6424995; doi:10.1186/s12284-019-0274-1)
Supplement: Supplementary file 2 — Figure S1. The aggressiveness of three BPH populations used for BPH resistance validation of mutant lines. Figure S2. Average AUC of 105 BILs and their parental varieties, KDML105 and RH. The selected extreme lines for susceptible and resistance individuals were lebeled in yellow and red respectively. Figure S3. Aggressiveness of three BPH population used for BPH resistance validation of mutant lines. (DOCX 23 kb) [file 12284_2019_274_MOESM2_ESM.docx]

Additional File 2

Identification of Spontaneous Mutation for Broad-Spectrum Brown Planthopper Resistance in a Large, Long-Term Fast Neutron Mutagenized Rice Population

Wintai Kamolsukyeunyong^1^, Siriphat Ruengphayak^2^, Pantharika Chumwong^1^, Ekawat Chaichumpoo^2^, Watchareewan Jamboonsri^1^, Chatree Saensuk^2^, Kunyakarn Phoonsiri^2^, Theerayut Toojinda^1, 3^ and Apichart Vanavichit^1, 2, 4^*

*** Correspondence:** Dr. Apichart Vanavichit: vanavichit@gmail.com

# Figure

**Figure S1** Average AUC of 105 BILs and their parental varieties, KDML105 and RH. The selected extreme lines for susceptible and resistance pools were lebeled in yellow and red respectively.

**Figure S2** Average AUC of 105 BILs and their parental varieties, KDML105 and RH. The selected extreme lines for susceptible and resistance individuals were lebeled in yellow and red respectively.

**Figure S3** Aggressiveness of three BPH population used for BPH resistance validation of mutant lines.
