# Supplementary material for: Identification of spontaneous mutation for broad-spectrum brown planthopper resistance in a large, long-term fast neutron mutagenized rice population
Source: Rice (N Y). 2019 Mar 19;12:16. doi: 10.1186/s12284-019-0274-1 (PMC6424995; doi:10.1186/s12284-019-0274-1)
Supplement: Supplementary file 3 — Data S1. Amino acid sequence analysis of OsLecRK1 gene in JHN, identified mutants and natural BPH resistance varieties, RH. Data S2. Amino acid sequence analysis of OsLecRK2 gene in JHN, identified mutants and natural BPH resistance varieties, RH. Data S3. Amino acid sequence analysis of OsLecRK3 gene in JHN, identified mutants and natural BPH resistance varieties, RH and PTB33. (DOCX 27 kb) [file 12284_2019_274_MOESM3_ESM.docx]

Additional File 3

Identification of Spontaneous Mutation for Broad-Spectrum Brown Planthopper Resistance in a Large, Long-Term Fast Neutron Mutagenized Rice Population

Wintai Kamolsukyeunyong^1^, Siriphat Ruengphayak^2^, Pantharika Chumwong^1^, Ekawat Chaichumpoo^2^, Watchareewan Jamboonsri^1^, Chatree Saensuk^2^, Kunyakarn Phoonsiri^2^, Theerayut Toojinda^1, 3^ and Apichart Vanavichit^1, 2, 4^*

*** Correspondence:** Dr. Apichart Vanavichit: vanavichit@gmail.com

# Data

**Data S1** Amino acid sequence analysis of OsLecRK3 gene in JHN, identified mutants and natural BPH resistance varieties, RH and PTB33

JHN00004_LecRK3 MAHLLFLPILQLLLLYCTKSAQAQLNISIGSSLTPQGVNNSWISPSADFAFGFRAVDGNS

JHN12005_LecRK3 MAHLLFLPILQLLLLYCTKSAQAQLNISIGSSLTPQGVNNSWISPSADFAFGFRAVDGNS

JHN19525_LecRK3 MAHLLFLPILQLLLLYCTKSAQAQLNISIGSSLTPQGVNNSWISPSADFAFGFRAVDGNS

JHN09962_LecRK3 MAHLLFLPILQLLLLYCTKSAQAQLNISIGSSLTPQGVNNSWISPSADFAFGFRAVDGNS

JHN19578_LecRK3 MAHLLFLPILQLLLLYCTKSAQAQLNISIGSSLTPQGVNNSWISPSADFAFGFRAVDGNS

JHN19874_LecRK3 MAHLLFLPILQLLLLYCTKSAQAQLNISIGSSLTPQGVNNSWISPSADFAFGFRAVDGNS

JHN12689_LecRK3 MAHLLFLPILQLLLLYCTKSAQAQLNISIGSSLTPQGVNNSWISPSADFAFGFRAVDGNS

JHN11183_LecRK3 MAHLLFLPILQLLLLYCTKSAQAQLNISIGSSLTPQGVNNSWISPSADFAFGFRAVDGNS

JHN05678_LecRK3 MAHLLFLPILQLLLLYCTKSAQAQLNISIGSSLTPQGVNNSWISPSADFAFGFRAVDGNS

PTH33_LecRK3 MAHLLFLPILQLLLLYCTKSAQAQLNISIGSSLTPQGVNNSWISPSADFAFGFRAVDGNS

RH_LecRK3 MAHLLFLPILQLLLLYCTKSAQAQLNISIGSSLTPQGVNNSWISPSADFAFGFRAVDGNS

JHN_LecRK3 MAHLLFLPILQLLLLYCTKSAQAQLNISIGSSLTPQGVNNSWISPSADFAFGFRAVDGNS

************************************************************

JHN00004_LecRK3 SSYLLAVWFNKIADKTVVWYARTSSNGQGDTMPVQVQSGSVLKLADGALSLRDPSGNEVW

JHN12005_LecRK3 SSYLLAVWFNKIADKTVVWYARTSSNGQGDTMPVQVQSGSVLKLADGALSLRDPSGNEVW

JHN19525_LecRK3 SSYLLAVWFNKIADKTVVWYARTSSNGQGDTMPVQVQSGSVLKLADGALSLRDPSGNEVW

JHN09962_LecRK3 SSYLLAVWFNKIADKTVVWYARTSSNGQGDTMPVQVQSGSVLKLADGALSLRDPSGNEVW

JHN19578_LecRK3 SSYLLAVWFNKIADKTVVWYARTSSNGQGDTMPVQVQSGSVLKLADGALSLRDPSGNEVW

JHN19874_LecRK3 SSYLLAVWFNKIADKTVVWYARTSSNGQGDTMPVQVQSGSVLKLADGALSLRDPSGNEVW

JHN12689_LecRK3 SSYLLAVWFNKIADKTVVWYARTSSNGQGDTMPVQVQSGSVLKLADGALSLRDPSGNEVW

JHN11183_LecRK3 SSYLLAVWFNKIADKTVVWYARTSSNGQGDTMPVQVQSGSVLKLADGALSLRDPSGNEVW

JHN05678_LecRK3 SSYLLAVWFNKIADKTVVWYARTSSNGQGDTMPVQVQSGSVLKLADGALSLRDPSGNEVW

PTH33_LecRK3 SSYLLAVWFNKIADKTVVWYARTSSNGQGDTMPVQVQSGSVLKLADGALSLRDPSGNEVW

RH_LecRK3 SSYLLAVWFNKIADKTVVWYARTSSNGQGDTMPVQVQSGSVLKLADGALSLRDPSGNEVW

JHN_LecRK3 SSYLLAVWFNKIAEKTVVWYARTSSNGKDDTIPVQVQSGSVLKLADGALSLRDPSGNEVW

*************:*************:.**:****************************

JHN00004_LecRK3 NPQVTDVGYARMLDTGNFRLLGTDGATKWESFGDPSDTILPTQVLSLGTALHSRLLATDY

JHN12005_LecRK3 NPQVTDVGYARMLDTGNFRLLGTDGATKWESFGDPSDTILPTQVLSLGTALHSRLLATDY

JHN19525_LecRK3 NPQVTDVGYARMLDTGNFRLLGTDGATKWESFGDPSDTILPTQVLSLGTALHSRLLATDY

JHN09962_LecRK3 NPQVTDVGYARMLDTGNFRLLGTDGATKWESFGDPSDTILPTQVLSLGTALHSRLLATDY

JHN19578_LecRK3 NPQVTDVGYARMLDTGNFRLLGTDGATKWESFGDPSDTILPTQVLSLGTALHSRLLATDY

JHN19874_LecRK3 NPQVTDVGYARMLDTGNFRLLGTDGATKWESFGDPSDTILPTQVLSLGTALHSRLLATDY

JHN12689_LecRK3 NPQVTDVGYARMLDTGNFRLLGTDGATKWESFGDPSDTILPTQVLSLGTALHSRLLATDY

JHN11183_LecRK3 NPQVTDVGYARMLDTGNFRLLGTDGATKWESFGDPSDTILPTQVLSLGTALHSRLLATDY

JHN05678_LecRK3 NPQVTDVGYARMLDTGNFRLLGTDGATKWESFGDPSDTILPTQVLSLGTALHSRLLATDY

PTH33_LecRK3 NPQVTDVGYARMLDTGNFRLLGTDGATKWESFGDPSDTILPTQVLSLGTALHSRLLATDY

RH_LecRK3 NPQVTDVGYARMLDTGNFRLLGTDGATKWESFGDPSDTILPTQVLSLGTALHSRLLATDY

JHN_LecRK3 NPQVTDVGYARMLDTGNFRLLGTDGATKWESFGDPSDTILPTQVLSLGTALHSRLLATDY

************************************************************

JHN00004_LecRK3 SNGRFQLKAQRDGNLVMYPDAVPSGYLYDPYWASNTVDNGSQLVFNETGRIYFTIINGSQ

JHN12005_LecRK3 SNGRFQLKAQRDGNLVMYPDAVPSGYLYDPYWASNTVDNGSQLVFNETGRIYFTIINGSQ

JHN19525_LecRK3 SNGRFQLKAQRDGNLVMYPDAVPSGYLYDPYWASNTVDNGSQLVFNETGRIYFTIINGSQ

JHN09962_LecRK3 SNGRFQLKAQRDGNLVMYPDAVPSGYLYDPYWASNTVDNGSQLVFNETGRIYFTIINGSQ

JHN19578_LecRK3 SNGRFQLKAQRDGNLVMYPDAVPSGYLYDPYWASNTVDNGSQLVFNETGRIYFTIINGSQ

JHN19874_LecRK3 SNGRFQLKAQRDGNLVMYPDAVPSGYLYDPYWASNTVDNGSQLVFNETGRIYFTIINGSQ

JHN12689_LecRK3 SNGRFQLKAQRDGNLVMYPDAVPSGYLYDPYWASNTVDNGSQLVFNETGRIYFTIINGSQ

JHN11183_LecRK3 SNGRFQLKAQRDGNLVMYPDAVPSGYLYDPYWASNTVDNGSQLVFNETGRIYFTIINGSQ

JHN05678_LecRK3 SNGRFQLKAQRDGNLVMYPDAVPSGYLYDPYWASNTVDNGSQLVFNETGRIYFTIINGSQ

PTH33_LecRK3 SNGRFQLKAQRDGNLVMYPDAVPSGYLYDPYWASNTVDNGSQLVFNETGRIYFTIINGSQ

RH_LecRK3 SNGRFQLKAQRDGNLVMYPDAVPSGYLYDPYWASNTVDNGSQLVFNETGRIYFTIINGSQ

JHN_LecRK3 SNGRFQLKVQRDGNLVMYPDAVPSGYLYDPYWASNTVDNGSQLVFNETGRIYFTIINGSQ

********.***************************************************

JHN00004_LecRK3 VNITSAGVDSMGDFFHRATLDTDGVFRQYVYPKNIHARPLWPEQWTAVDVLPENICQSIQ

JHN12005_LecRK3 VNITSAGVDSMGDFFHRATLDTDGVFRQYVYPKNIHARPLWPEQWTAVDVLPENICQSIQ

JHN19525_LecRK3 VNITSAGVDSMGDFFHRATLDTDGVFRQYVYPKNIHARPLWPEQWTAVDVLPENICQSIQ

JHN09962_LecRK3 VNITSAGVDSMGDFFHRATLDTDGVFRQYVYPKNIHARPLWPEQWTAVDVLPENICQSIQ

JHN19578_LecRK3 VNITSAGVDSMGDFFHRATLDTDGVFRQYVYPKNIHARPLWPEQWTAVDVLPENICQSIQ

JHN19874_LecRK3 VNITSAGVDSMGDFFHRATLDTDGVFRQYVYPKNIHARPLWPEQWTAVDVLPENICQSIQ

JHN12689_LecRK3 VNITSAGVDSMGDFFHRATLDTDGVFRQYVYPKNIHARPLWPEQWTAVDVLPENICQSIQ

JHN11183_LecRK3 VNITSAGVDSMGDFFHRATLDTDGVFRQYVYPKNIHARPLWPEQWTAVDVLPENICQSIQ

JHN05678_LecRK3 VNITSAGVDSMGDFFHRATLDTDGVFRQYVYPKNIHARPLWPEQWTAVDVLPENICQSIQ

PTH33_LecRK3 VNITSAGVDSMGDFFHRATLDTDGVFRQYVYPKNIHARPLWPEQWTAVDVLPENICQSIQ

RH_LecRK3 VNITSAGVDSMGDFFHRATLDTDGVFRQYVYPKNIHARPLWPEQWTAVDVLPENICQSIQ

JHN_LecRK3 VNITSAGVDSMGDFFHRATLDTDGVFRQYVYPKNIHARPLWPEQWTAVDVLPENICQSIQ

************************************************************

JHN00004_LecRK3 TMVGSGACGFNSYCTIDGTKNTTSCLCPQNYKFIDDKRKYKGCRPDFEPQNCDLDETTAM

JHN12005_LecRK3 TMVGSGACGFNSYCTIDGTKNTTSCLCPQNYKFIDDKRKYKGCRPDFEPQNCDLDETTAM

JHN19525_LecRK3 TMVGSGACGFNSYCTIDGTKNTTSCLCPQNYKFIDDKRKYKGCRPDFEPQNCDLDETTAM

JHN09962_LecRK3 TMVGSGACGFNSYCTIDGTKNTTSCLCPQNYKFIDDKRKYKGCRPDFEPQNCDLDETTAM

JHN19578_LecRK3 TMVGSGACGFNSYCTIDGTKNTTSCLCPQNYKFIDDKRKYKGCRPDFEPQNCDLDETTAM

JHN19874_LecRK3 TMVGSGACGFNSYCTIDGTKNTTSCLCPQNYKFIDDKRKYKGCRPDFEPQNCDLDETTAM

JHN12689_LecRK3 TMVGSGACGFNSYCTIDGTKNTTSCLCPQNYKFIDDKRKYKGCRPDFEPQNCDLDETTAM

JHN11183_LecRK3 TMVGSGACGFNSYCTIDGTKNTTSCLCPQNYKFIDDKRKYKGCRPDFEPQNCDLDETTAM

JHN05678_LecRK3 TMVGSGACGFNSYCTIDGTKNTTSCLCPQNYKFIDDKRKYKGCRPDFEPQNCDLDETTAM

PTH33_LecRK3 TMVGSGACGFNSYCTIDGTKNTTSCLCPQNYKFIDDKRKYKGCRPDFEPQNCDLDETTAM

RH_LecRK3 TMVGSGACGFNSYCTIDGTKNTTSCLCPQNYKFIDDKRKYKGCRPDFEPQNCDLDETTAM

JHN_LecRK3 TMVGSGACGFNSYCTIDGTKNTTSCLCPQNYKFIDDKRKYKGCRPDFEPQNCDLDETTAM

************************************************************

JHN00004_LecRK3 LQYDMAPIDRVDWPLSDYEQYNPIDQTECRRLCVIDCFCAVAVFDKASSTCWKKRFPLSN

JHN12005_LecRK3 LQYDMAPIDRVDWPLSDYEQYNPIDQTECRRLCVIDCFCAVAVFDKASSTCWKKRFPLSN

JHN19525_LecRK3 LQYDMAPIDRVDWPLSDYEQYNPIDQTECRRLCVIDCFCAVAVFDKASSTCWKKRFPLSN

JHN09962_LecRK3 LQYDMAPIDRVDWPLSDYEQYNPIDQTECRRLCVIDCFCAVAVFDKASSTCWKKRFPLSN

JHN19578_LecRK3 LQYDMAPIDRVDWPLSDYEQYNPIDQTECRRLCVIDCFCAVAVFDKASSTCWKKRFPLSN

JHN19874_LecRK3 LQYDMAPIDRVDWPLSDYEQYNPIDQTECRRLCVIDCFCAVAVFDKASSTCWKKRFPLSN

JHN12689_LecRK3 LQYDMAPIDRVDWPLSDYEQYNPIDQTECRRLCVIDCFCAVAVFDKASSTCWKKRFPLSN

JHN11183_LecRK3 LQYDMAPIDRVDWPLSDYEQYNPIDQTECRRLCVIDCFCAVAVFDKASSTCWKKRFPLSN

JHN05678_LecRK3 LQYDMAPIDRVDWPLSDYEQYNPIDQTECRRLCVIDCFCAVAVFDKASSTCWKKRFPLSN

PTH33_LecRK3 LQYDMAPIDRVDWPLSDYEQYNPIDQTECRRLCVIDCFCAVAVFDKASSTCWKKRFPLSN

RH_LecRK3 LQYDMAPIDRVDWPLSDYEQYNPIDQTECRRLCVIDCFCAVAVFDKASSTCWKKRFPLSN

JHN_LecRK3 LQYDMAPIDRVDWPLSDYEQYNPIDQTECRRLCVIDCFCAVAVFDKASSTCWKKRFPLSN

************************************************************

JHN00004_LecRK3 GKMDVNVPRTVLIKVPRSTNSPSVFSSGSSKWKEDKKYWILGSSLLFGSSVLVNFLLISV

JHN12005_LecRK3 GKMDVNVPRTVLIKVPRSTNSPSVFSSGSSKWKEDKKYWILGSSLLFGSSVLVNFLLISV

JHN19525_LecRK3 GKMDVNVPRTVLIKVPRSTNSPSVFSSGSSKWKEDKKYWILGSSLLFGSSVLVNFLLISV

JHN09962_LecRK3 GKMDVNVPRTVLIKVPRSTNSPSVFSSGSSKWKEDKKYWILGSSLLFGSSVLVNFLLISV

JHN19578_LecRK3 GKMDVNVPRTVLIKVPRSTNSPSVFSSGSSKWKEDKKYWILGSSLLFGSSVLVNFLLISV

JHN19874_LecRK3 GKMDVNVPRTVLIKVPRSTNSPSVFSSGSSKWKEDKKYWILGSSLLFGSSVLVNFLLISV

JHN12689_LecRK3 GKMDVNVPRTVLIKVPRSTNSPSVFSSGSSKWKEDKKYWILGSSLLFGSSVLVNFLLISV

JHN11183_LecRK3 GKMDVNVPRTVLIKVPRSTNSPSVFSSGSSKWKEDKKYWILGSSLLFGSSVLVNFLLISV

JHN05678_LecRK3 GKMDVNVPRTVLIKVPRSTNSPSVFSSGSSKWKEDKKYWILGSSLLFGSSVLVNFLLISV

PTH33_LecRK3 GKMDVNVPRTVLIKVPRSTNSPSVFSSGSSKWKEDKKYWILGSSLLFGSSVLVNFLLISV

RH_LecRK3 GKMDVNVPRTVLIKVPRSTNSPSVFSSGSSKWKEDKKYWILGSSLLFGSSVLVNFLLISV

JHN_LecRK3 GKMDVNVPRTVLIKVPRSTNSPSVFSSGSSKWKEDKKYWILGSSLLFGSSVLVNFLLISV

************************************************************

JHN00004_LecRK3 MLFGTYCSITSRKKIQLSQPSNNSGLPPKIFTYSELEKATGGFQEVLGTGASGVVYKGQL

JHN12005_LecRK3 MLFGTYCSITSRKKIQLSQPSNNSGLPPKIFTYSELEKATGGFQEVLGTGASGVVYKGQL

JHN19525_LecRK3 MLFGTYCSITSRKKIQLSQPSNNSGLPPKIFTYSELEKATGGFQEVLGTGASGVVYKGQL

JHN09962_LecRK3 MLFGTYCSITSRKKIQLSQPSNNSGLPPKIFTYSELEKATGGFQEVLGTGASGVVYKGQL

JHN19578_LecRK3 MLFGTYCSITSRKKIQLSQPSNNSGLPPKIFTYSELEKATGGFQEVLGTGASGVVYKGQL

JHN19874_LecRK3 MLFGTYCSITSRKKIQLSQPSNNSGLPPKIFTYSELEKATGGFQEVLGTGASGVVYKGQL

JHN12689_LecRK3 MLFGTYCSITSRKKIQLSQPSNNSGLPPKIFTYSELEKATGGFQEVLGTGASGVVYKGQL

JHN11183_LecRK3 MLFGTYCSITSRKKIQLSQPSNNSGLPPKIFTYSELEKATGGFQEVLGTGASGVVYKGQL

JHN05678_LecRK3 MLFGTYCSITSRKKIQLSQPSNNSGLPPKIFTYSELEKATGGFQEVLGTGASGVVYKGQL

PTH33_LecRK3 MLFGTYCSITSRKKIQLSQPSNNSGLPPKIFTYSELEKATGGFQEVLGTGASGVVYKGQL

RH_LecRK3 MLFGTYCSITSRKKIQLSQPSNNSGLPPKIFTYSELEKATGGFQEVLGTGASGVVYKGQL

JHN_LecRK3 MLFGTYCSITSRKKIQLSQPSNNSGLPPKIFTYSELEKATGGFQEVLGTGASGVVYKGQV

***********************************************************:

JHN00004_LecRK3 QDEFGTNIAVKKIEKLQQEAQKEFLVEVQTIGQTFHRNLVRLLGFCNEGTERLLVYEFMS

JHN12005_LecRK3 QDEFGTNIAVKKIEKLQQEAQKEFLVEVQTIGQTFHRNLVRLLGFCNEGTERLLVYEFMS

JHN19525_LecRK3 QDEFGTNIAVKKIEKLQQEAQKEFLVEVQTIGQTFHRNLVRLLGFCNEGTERLLVYEFMS

JHN09962_LecRK3 QDEFGTNIAVKKIEKLQQEAQKEFLVEVQTIGQTFHRNLVRLLGFCNEGTERLLVYEFMS

JHN19578_LecRK3 QDEFGTNIAVKKIEKLQQEAQKEFLVEVQTIGQTFHRNLVRLLGFCNEGTERLLVYEFMS

JHN19874_LecRK3 QDEFGTNIAVKKIEKLQQEAQKEFLVEVQTIGQTFHRNLVRLLGFCNEGTERLLVYEFMS

JHN12689_LecRK3 QDEFGTNIAVKKIEKLQQEAQKEFLVEVQTIGQTFHRNLVRLLGFCNEGTERLLVYEFMS

JHN11183_LecRK3 QDEFGTNIAVKKIEKLQQEAQKEFLVEVQTIGQTFHRNLVRLLGFCNEGTERLLVYEFMS

JHN05678_LecRK3 QDEFGTNIAVKKIEKLQQEAQKEFLVEVQTIGQTFHRNLVRLLGFCNEGTERLLVYEFMS

PTH33_LecRK3 QDEFGTNIAVKKIEKLQQEAQKEFLVEVQTIGQTFHRNLVRLLGFCNEGTERLLVYEFMS

RH_LecRK3 QDEFGTNIAVKKIEKLQQEAQKEFLVEVQTIGQTFHRNLVRLLGFCNEGTERLLVYEFMS

JHN_LecRK3 QDEFGTNIAVKKIEKLQQEAQKEFLVEVQTIGQTFHRNLVRLLGFCNEGTEKLLVYEFMS

***************************************************:********

JHN00004_LecRK3 NGSLNTFLFSDTHPHWSLRVQVALGVARGLLYLHEECNKQIIHCDMKPQNILLDDNFVAK

JHN12005_LecRK3 NGSLNTFLFSDTHPHWSLRVQVALGVARGLLYLHEECNKQIIHCDMKPQNILLDDNFVAK

JHN19525_LecRK3 NGSLNTFLFSDTHPHWSLRVQVALGVARGLLYLHEECNKQIIHCDMKPQNILLDDNFVAK

JHN09962_LecRK3 NGSLNTFLFSDTHPHWSLRVQVALGVARGLLYLHEECNKQIIHCDMKPQNILLDDNFVAK

JHN19578_LecRK3 NGSLNTFLFSDTHPHWSLRVQVALGVARGLLYLHEECNKQIIHCDMKPQNILLDDNFVAK

JHN19874_LecRK3 NGSLNTFLFSDTHPHWSLRVQVALGVARGLLYLHEECNKQIIHCDMKPQNILLDDNFVAK

JHN12689_LecRK3 NGSLNTFLFSDTHPHWSLRVQVALGVARGLLYLHEECNKQIIHCDMKPQNILLDDNFVAK

JHN11183_LecRK3 NGSLNTFLFSDTHPHWSLRVQVALGVARGLLYLHEECNKQIIHCDMKPQNILLDDNFVAK

JHN05678_LecRK3 NGSLNTFLFSDTHPHWSLRVQVALGVARGLLYLHEECNKQIIHCDMKPQNILLDDNFVAK

PTH33_LecRK3 NGSLNTFLFSDTHPHWSLRVQVALGVARGLLYLHEECNKQIIHCDMKPQNILLDDNFVAK

RH_LecRK3 NGSLNTFLFSDTHPHWSLRVQVALGVARGLLYLHEECNKQIIHCDMKPQNILLDDNFVAK

JHN_LecRK3 NGSLNTFLFSDTHPHWSLRVQVALGVSRGLLYLHEECNKQIIHCDMKPQNILLDDNFVAK

**************************:*********************************

JHN00004_LecRK3 ISDFGLAKLLPVNQTQTNTGIRGTRGYVAPEWFKNIGITSKVDVYSFGVILLELVCCRKN

JHN12005_LecRK3 ISDFGLAKLLPVNQTQTNTGIRGTRGYVAPEWFKNIGITSKVDVYSFGVILLELVCCRKN

JHN19525_LecRK3 ISDFGLAKLLPVNQTQTNTGIRGTRGYVAPEWFKNIGITSKVDVYSFGVILLELVCCRKN

JHN09962_LecRK3 ISDFGLAKLLPVNQTQTNTGIRGTRGYVAPEWFKNIGITSKVDVYSFGVILLELVCCRKN

JHN19578_LecRK3 ISDFGLAKLLPVNQTQTNTGIRGTRGYVAPEWFKNIGITSKVDVYSFGVILLELVCCRKN

JHN19874_LecRK3 ISDFGLAKLLPVNQTQTNTGIRGTRGYVAPEWFKNIGITSKVDVYSFGVILLELVCCRKN

JHN12689_LecRK3 ISDFGLAKLLPVNQTQTNTGIRGTRGYVAPEWFKNIGITSKVDVYSFGVILLELVCCRKN

JHN11183_LecRK3 ISDFGLAKLLPVNQTQTNTGIRGTRGYVAPEWFKNIGITSKVDVYSFGVILLELVCCRKN

JHN05678_LecRK3 ISDFGLAKLLPVNQTQTNTGIRGTRGYVAPEWFKNIGITSKVDVYSFGVILLELVCCRKN

PTH33_LecRK3 ISDFGLAKLLPVNQTQTNTGIRGTRGYVAPEWFKNIGITSKVDVYSFGVILLELVCCRKN

RH_LecRK3 ISDFGLAKLLPVNQTQTNTGIRGTRGYVAPEWFKNIGITSKVDVYSFGVILLELVCCRKN

JHN_LecRK3 ISDFGLAKLLPVNQTQTNTGIRGTRGYVAPEWFKNIGITSKVDVYSFGVIVLELVCCRKN

**************************************************:*********

JHN00004_LecRK3 VELEVLDEEQTILTYWANDCYKCGRIDLLVAGDDEAIFNIKKVERFVAVALWCLQEEPSM

JHN12005_LecRK3 VELEVLDEEQTILTYWANDCYKCGRIDLLVAGDDEAIFNIKKVERFVAVALWCLQEEPSM

JHN19525_LecRK3 VELEVLDEEQTILTYWANDCYKCGRIDLLVAGDDEAIFNIKKVERFVAVALWCLQEEPSM

JHN09962_LecRK3 VELEVLDEEQTILTYWANDCYKCGRIDLLVAGDDEAIFNIKKVERFVAVALWCLQEEPSM

JHN19578_LecRK3 VELEVLDEEQTILTYWANDCYKCGRIDLLVAGDDEAIFNIKKVERFVAVALWCLQEEPSM

JHN19874_LecRK3 VELEVLDEEQTILTYWANDCYKCGRIDLLVAGDDEAIFNIKKVERFVAVALWCLQEEPSM

JHN12689_LecRK3 VELEVLDEEQTILTYWANDCYKCGRIDLLVAGDDEAIFNIKKVERFVAVALWCLQEEPSM

JHN11183_LecRK3 VELEVLDEEQTILTYWANDCYKCGRIDLLVAGDDEAIFNIKKVERFVAVALWCLQEEPSM

JHN05678_LecRK3 VELEVLDEEQTILTYWANDCYKCGRIDLLVAGDDEAIFNIKKVERFVAVALWCLQEEPSM

PTH33_LecRK3 VELEVLDEEQTILTYWANDCYKCGRIDLLVAGDDEAIFNIKKVERFVAVALWCLQEEPSM

RH_LecRK3 VELEVLDEEQTILTYWANDCYKCGRIDLLVAGDDEAIFNIKKVERFVAVALWCLQEEPSM

JHN_LecRK3 VELEVLDEEQTILTYWANDCYKCGRIDLLVAGDDEAIFNIKKVERFVAVALWCLQEEPSM

************************************************************

JHN00004_LecRK3 RPTMLKVTQMLDGAVQIPTPPDPSSYISSLA

JHN12005_LecRK3 RPTMLKVTQMLDGAVQIPTPPDPSSYISSLA

JHN19525_LecRK3 RPTMLKVTQMLDGAVQIPTPPDPSSYISSLA

JHN09962_LecRK3 RPTMLKVTQMLDGAVQIPTPPDPSSYISSLA

JHN19578_LecRK3 RPTMLKVTQMLDGAVQIPTPPDPSSYISSLA

JHN19874_LecRK3 RPTMLKVTQMLDGAVQIPTPPDPSSYISSLA

JHN12689_LecRK3 RPTMLKVTQMLDGAVQIPTPPDPSSYISSLA

JHN11183_LecRK3 RPTMLKVTQMLDGAVQIPTPPDPSSYISSLA

JHN05678_LecRK3 RPTMLKVTQMLDGAVQIPTPPDPSSYISSLA

PTH33_LecRK3 RPTMLKVTQMLDGAVQIPTPPDPSSYISSLA

RH_LecRK3 RPTMLKVTQMLDGAVQIPTPPDPSSYISSLA

JHN_LecRK3 RPTMLKVTQMLDGAVQIPTPPDPSSYISSLA

*************************************

**Data S2** Amino acid sequence analysis of OsLecRK1 gene in JHN, identified mutants and natural BPH resistance varieties, RH.

JHN_LecRK1 MVALLLFPMLLQLLSPTCAQTQKNITLGSTLAPQSPASSWLSPSGDFAFGFRPVEGNTSF

JHN11183_LecRK1 MVALLLFPMLLQLLSPTCAQTQKNITLGSTLAPQGPASSWLSPSGDFAFGFRPVEGNTSF

JHN19525_LecRK1 MVALLLFPMLLQLLSPTCAQTQKNITLGSTLAPQGPASSWLSPSGDFAFGFRPVEGNTSF

JHN09962_LecRK1 MVALLLFPMLLQLLSPTCAQTQKNITLGSTLAPQGPASSWLSPSGDFAFGFRPVEGNTSF

JHN19578_LecRK1 MVALLLFPMLLQLLSPTCAQTQKNITLGSTLAPQGPASSWLSPSGDFAFGFRPVEGNTSF

JHN19874_LecRK1 MVALLLFPMLLQLLSPTCAQTQKNITLGSTLAPQGPASSWLSPSGDFAFGFRPVEGNTSF

JHN09962_LecRK1 MVALLLFPMLLQLLSPTCAQTQKNITLGSTLAPQGPASSWLSPSGDFAFGFRPVEGNTSF

RH_LecRK1 MVALLLFPMLLQLLSPTCAQTQKNITLGSTLAPQGPASSWLSPSGDFAFGFRPVEGNTSF

JHN19671_LecRK1 MVALLLFPMLLQLLSPTCAQTQKNITLGSTLAPQGPASSWLSPSGDFAFGFRPVKGNTSF

JHNMT2_LecRK1 MVALLLFPMLLQLLSPTCAQTQKNITLGSTLAPQGPASSWLSPSGDFAFGFRPVEGNTSF

JHNMT1_LecRK1 MVALLLFPMLLQLLSPTCAQTQKNITLGSTLAPQGPASSWLSPSGDFAFGFRPVEGNTSF

**********************************.*******************:*****

JHN_LecRK1 YLIAVWFNKISDKTVVWYAKNTDQDPSIVEVPSDSFLQLTNDGALSLKDRSGQEGWNPQV

JHN11183_LecRK1 YLIAVWFNKISDKTVVWYAKNTDQDPSIVEVPSDSFLQLTNDGALSLKDRSGQEGWNPQV

JHN19525_LecRK1 YLIAVWFNKISDKTVVWYAKNTDQDPSIVKVPSDSFLQLTNDGALSLKDRSGQEGWNPQV

JHN09962_LecRK1 YLIAVWFNKISDKTVVWYAKNTDQDPSIVKVPSDSFLQLTNDGALSLKDRSGQEGWNPQV

JHN19578_LecRK1 YLIAVWFNKISDKTVVWYAKNTDQDPSIVKVPSDSFLQLTNDGALSLKDRSGQEGWNPQV

JHN19874_LecRK1 YLIAVWFNKISDKTVVWYAKNTDQDPSIVKVPSDSFLQLTNDGALSLKDRSGQEGWNPQV

JHN09962_LecRK1 YLIAVWFNKISDKTVVWYAKNTDQDPSIVKVPSDSFLQLTNDGALSLKDRSGQEGWNPQV

RH_LecRK1 YLIAVWFNKISDKTVVWYAKNTDQDPSIVKVPSDSFLQLTNDGALSLKDRSGQEGWNPQV

JHN19671_LecRK1 YLIAVWFNKISDKTVVWYAKNTDQNPSIVEVPSDSFLQLTNDGALSLKDRSGQEGWNPQV

JHNMT2_LecRK1 YLIAVWFNKISDKTVVWYAKNTDQDPSIVEVPSDSFLQLTNDGALSLKDRSGQEGWNPQV

JHNMT1_LecRK1 YLIAVWFNKISDKTVVWYAKNTDQDPSIVEVPSDSFLQLTNDGALSLKDRSGQEGWNPQV

************************:****:******************************

JHN_LecRK1 TSVAYASMRDTGNFVLLGADGTTKWQTFDMPSDTILPTQVIPCNKTRNKSLRARLDIDDY

JHN11183_LecRK1 TGVAYASMCDTGNFVLLGADGTTKWQTFDMPSDTILPTQVIPCNKTRNKSLRARLDIDDY

JHN19525_LecRK1 TGVAYASMCDTGNFVLLGADGTTKWQTFDMPSDTILPTQVIPCNKTRNKSLRARLDIDDY

JHN09962_LecRK1 TGVAYASMCDTGNFVLLGADGTTKWQTFDMPSDTILPTQVIPCNKTRNKSLRARLDIDDY

JHN19578_LecRK1 TGVAYASMCDTGNFVLLGADGTTKWQTFDMPSDTILPTQVIPCNKTRNKSLRARLDIDDY

JHN19874_LecRK1 TGVAYASMCDTGNFVLLGADGTTKWQTFDMPSDTILPTQVIPCNKTRNKSLRARLDIDDY

JHN09962_LecRK1 TGVAYASMCDTGNFVLLGADGTTKWQTFDMPSDTILPTQVIPCNKTRNKSLRARLDIDDY

RH_LecRK1 TGVAYASMCDTGNFVLLGADGTTKWQTFDMPSDTILPTQVIPCNKTRNKSLRARLDIDDY

JHN19671_LecRK1 TGVAYASMRDTGNFVLLGADGTTKWQTFDMPSDTILPTQVIPCNKTRNKSLRARLDIDDY

JHNMT2_LecRK1 TGVAYASMRDTGNFVLLGADGTTKWQTFDMPSDTILPTQVIPCNKTRNKSLRARLDIDDY

JHNMT1_LecRK1 TGVAYASMRDTGNFVLLGADGTTKWQTFDMPSDTILPTQVIPCNKTRNKSLRARLDIDDY

*.****** ***************************************************

JHN_LecRK1 SSGRFLLDVQTDGNLALYLVAVPSGSKYQQYWSTDTTGNGSELVFSETGKVYFALTDGTQ

JHN11183_LecRK1 SSGRFLLDVQTDGNLALYLVAVPSGSKYQQYWSTDTTGNGSELVFSETGKVYFALTDGTQ

JHN19525_LecRK1 SSGRFLLDVQTDGNLALYLVAVPSGSKYQQYWSTDTTGNGSELVFSETGKVYFALTDGTQ

JHN09962_LecRK1 SSGRFLLDVQTDGNLALYLVAVPSGSKYQQYWSTDTTGNGSELVFSETGKVYFALTDGTQ

JHN19578_LecRK1 SSGRFLLDVQTDGNLALYLVAVPSGSKYQQYWSTDTTGNGSELVFSETGKVYFALTDGTQ

JHN19874_LecRK1 SSGRFLLDVQTDGNLALYLVAVPSGSKYQQYWSTDTTGNGSELVFSETGKVYFALTDGTQ

JHN09962_LecRK1 SSGRFLLDVQTDGNLALYLVAVPSGSKYQQYWSTDTTGNGSELVFSETGKVYFALTDGTQ

RH_LecRK1 SSGRFLLDVQTDGNLALYLVAVPSGSKYQQYWSTDTTGNGSELVFSETGKVYFALTDGTQ

JHN19671_LecRK1 SSGRFLLDVQTDGNLALYLVAVPSGSKYQQYWSTDTTGNGSELVFSETGKVYFALTDGTQ

JHNMT2_LecRK1 SSGRFLLDVQTDGNLALYLVAVPSGSKYQQYWSTDTTGNGSELVFSETGKVYFALTDGTQ

JHNMT1_LecRK1 SSGRFLLDVQTDGNLALYLVAVPSGSKYQQYWSTDTTGNGSELVFSETGKVYFALTDGTQ

************************************************************

JHN_LecRK1 INISSGAGIGSMADYFHRATLDPDGVFRQYVYPKKANAGILGGETWTAVSMQPQNICHAI

JHN11183_LecRK1 INISSDAGIGSMADYFHRATLDPDGVFRQYVYPKKANAGILGGETWTALSMQPQNICHAI

JHN19525_LecRK1 INISSDAGIGSMADYFHRATLDPDGVFRQYVYPKKANAGILGGETWTALSMQPQNICHAI

JHN09962_LecRK1 INISSDAGIGSMADYFHRATLDPDGVFRQYVYPKKANAGILGGETWTALSMQPQNICHAI

JHN19578_LecRK1 INISSDAGIGSMADYFHRATLDPDGVFRQYVYPKKANAGILGGETWTALSMQPQNICHAI

JHN19874_LecRK1 INISSDAGIGSMADYFHRATLDPDGVFRQYVYPKKANAGILGGETWTALSMQPQNICHAI

JHN09962_LecRK1 INISSDAGIGSMADYFHRATLDPDGVFRQYVYPKKANAGILGGETWTALSMQPQNICHAI

RH_LecRK1 INISSDAGIGSMADYFHRATLDPDGVFRQYVYPKKANAGILGGETWTALSMQPQNICHAI

JHN19671_LecRK1 INISSDAGIGSMADYFHRATLDPDGVFRQYVYPKKANAGILGGETWTALSMQPQNICHAI

JHNMT2_LecRK1 INISSDAGIGSMADYFHRATLDPDGVFRQYVYPKKANAGILGGETWTALSMQPQNICHAI

JHNMT1_LecRK1 INISSDAGIGSMADYFHRATLDPDGVFRQYVYPKKANAGILGGETWTALSMQPQNICHAI

*****.******************************************:***********

JHN_LecRK1 VSDVGSGVCGFNSYCTFDGTRNQIASCQCPPWYKFFDEQKKYKGCKQDFQPHSCDLDEAT

JHN11183_LecRK1 VSDVGSGVCGFNSYCTFDGTRNQIASCQCPPWYKFFDEQKKYKGCKQDFQPHSCDLEEAT

JHN19525_LecRK1 VSDVGSGVCGFNSYCTFDGTRNQIASCQCPPWYKFFDEQKKYKGCKQDFQPHSCDLEEAT

JHN09962_LecRK1 VSDVGSGVCGFNSYCTFDGTRNQIASCQCPPWYKFFDEQKKYKGCKQDFQPHSCDLEEAT

JHN19578_LecRK1 VSDVGSGVCGFNSYCTFDGTRNQIASCQCPPWYKFFDEQKKYKGCKQDFQPHSCDLEEAT

JHN19874_LecRK1 VSDVGSGVCGFNSYCTFDGTRNQIASCQCPPWYKFFDEQKKYKGCKQDFQPHSCDLEEAT

JHN09962_LecRK1 VSDVGSGVCGFNSYCTFDGTRNQIASCQCPPWYKFFDEQKKYKGCKQDFQPHSCDLEEAT

RH_LecRK1 VSDVGSGVCGFNSYCTFDGTRNQIASCQCPPWYKFFDEQKKYKGCKQDFQPHSCDLEEAT

JHN19671_LecRK1 VSDVGSGVCGFNSYCTFDGTRNQIASCQCPPWYKFFDEQKKYKGCKQDFQPHSCDLEEAT

JHNMT2_LecRK1 VSDVGSGVCGFNSYCTFDGTRNQIASCQCPPWYKFFDEQKKYKGCKQDFQPHSCDLEEAT

JHNMT1_LecRK1 VSDVGSGVCGFNSYCTFDGTRNQIASCQCPPWYKFFDEQKKYKGCKQDFQPHSCDLEEAT

********************************************************:***

JHN_LecRK1 ALAQFELRPIYGVDWPLSDYEKYCSFEPIGQDDCGRLCMIDCFCAMAVYNQSTSTCWKKK

JHN11183_LecRK1 ALAQFELRPIYGVDWPLSDYEKY---EPIGQDDCGRLCVIECFCAMAVYNQSTSTCWKKK

JHN19525_LecRK1 ALAQFELRPIYGVDWPLSDYEKY---EPIGQDDCGRLCVIECFCAMAVYNQSTSTCWKKK

JHN09962_LecRK1 ALAQFELRPIYGVDWPLSDYEKY---EPIGQDDCGRLCVIECFCAMAVYNQSTSTCWKKK

JHN19578_LecRK1 ALAQFELRPIYGVDWPLSDYEKY---EPIGQDDCGRLCVIECFCAMAVYNQSTSTCWKKK

JHN19874_LecRK1 ALAQFELRPIYGVDWPLSDYEKY---EPIGQDDCGRLCVIECFCAMAVYNQSTSTCWKKK

JHN09962_LecRK1 ALAQFELRPIYGVDWPLSDYEKY---EPIGQDDCGRLCVIECFCAMAVYNQSTSTCWKKK

RH_LecRK1 ALAQFELRPIYGVDWPLSDYEKY---EPIGQDDCGRLCVIECFCAMAVYNQSTSTCWKKK

JHN19671_LecRK1 ALAQFELRPIYGVDWPLSDYEKY---EPIGQDDCGRLCVIECFCAMAVYNQSTSTCWKKK

JHNMT2_LecRK1 ALAQFELRPIYGVDWPLSDYEKY---EPIGQDDCGRLCVIECFCAMAVYNQSTSTCWKKK

JHNMT1_LecRK1 ALAQFELRPIYGVDWPLSDYEKY---EPIGQDDCGRLCVIECFCAMAVYNQSTSTCWKKK

*********************** ************:*:*******************

JHN_LecRK1 LPLSNGNMADYVQRTVLLKVPSSNSSQSMISTSSNKWKRNRKHWVLGSSLILGTSILVNF

JHN11183_LecRK1 LPLSNGNMADYVQRTVLLKVPSSNSSQFMISTSSNKWKRNRKHWVLGSSLILGTSILVNF

JHN19525_LecRK1 LPLSNGNMADYVQRTVLLKVPSSNSSQFMISTSSNKWKRNRKHWVLGSSLILGTSILVNF

JHN09962_LecRK1 LPLSNGNMADYVQRTVLLKVPSSNSSQFMISTSSNKWKRNRKHWVLGSSLILGTSILVNF

JHN19578_LecRK1 LPLSNGNMADYVQRTVLLKVPSSNSSQFMISTSSNKWKRNRKHWVLGSSLILGTSILVNF

JHN19874_LecRK1 LPLSNGNMADYVQRTVLLKVPSSNSSQFMISTSSNKWKRNRKHWVLGSSLILGTSILVNF

JHN09962_LecRK1 LPLSNGNMADYVQRTVLLKVPSSNSSQFMISTSSNKWKRNRKHWVLGSSLILGTSILVNF

RH_LecRK1 LPLSNGNMADYVQRTVLLKVPSSNSSQFMISTSSNKWKRNRKHWVLGSSLILGTSILVNF

JHN19671_LecRK1 LPLSNGNMADYVQRTVLLKVPSSNSSQFMISTSSNKWKRNRKHWVLGSSLILGTSILVNF

JHNMT2_LecRK1 LPLSNGNMADYVQRTVLLKVPSSNSSQFMISTSSNKWKRNRKHWVLGSSLILGTSILVNF

JHNMT1_LecRK1 LPLSNGNMADYVQRTVLLKVPSSNSSQFMISTSSNKWKRNRKHWVLGSSLILGTSILVNF

*************************** ********************************

JHN_LecRK1 ALISIFLFGTYCRIATKKNIPLSQASSKSQLPLKTFTYKELEKATAGFHEILGAGASGVV

JHN11183_LecRK1 ALISIFLFGTYCRITTKKNIPLSQASSKSQLPLKTFTYKELEKATAGFHEILGAGASGVV

JHN19525_LecRK1 ALISIFLFGTYCRITTKKNIPLSQASSKSQLPLKTFTYKELEKATAGFHEILGAGASGVV

JHN09962_LecRK1 ALISIFLFGTYCRITTKKNIPLSQASSKSQLPLKTFTYKELEKATAGFHEILGAGASGVV

JHN19578_LecRK1 ALISIFLFGTYCRITTKKNIPLSQASSKSQLPLKTFTYKELEKATAGFHEILGAGASGVV

JHN19874_LecRK1 ALISIFLFGTYCRITTKKNIPLSQASSKSQLPLKTFTYKELEKATAGFHEILGAGASGVV

JHN09962_LecRK1 ALISIFLFGTYCRITTKKNIPLSQASSKSQLPLKTFTYKELEKATAGFHEILGAGASGVV

RH_LecRK1 ALISIFLFGTYCRITTKKNIPLSQASSKSQLPLKTFTYKELEKATAGFHEILGAGASGVV

JHN19671_LecRK1 ALISIFLFGTYCRITTKKNIPLSQASSKSQLPLKTFTYKELEKATAGFHEILGASASGVV

JHNMT2_LecRK1 ALISIFLFGTYCRITTKKNIPLSQASSKSQLPLKTFTYKELEKATAGFHEILGASASGVV

JHNMT1_LecRK1 ALISIFLFGTYCRITTKKNIPLSQASSKSQLPLKTFTYKELEKATAGFHEILGASASGVV

**************:***************************************.*****

JHN_LecRK1 YKGQLEDELKTNIAVKKIDKLQPETEKEFMVEVETIGQTFHKNLVRLLGFCNEGAERLLV

JHN11183_LecRK1 YKGQLEDELKTNIAVKTIHKLQPETEKEFMVEVETIGQTFHKNLVRLLGFCNERAERLLV

JHN19525_LecRK1 YKGQLEDELKTNIAVKTIHKLQPETEKEFMVEVETIGQTFHKNLVRLLGFCNERAERLLV

JHN09962_LecRK1 YKGQLEDELKTNIAVKTIHKLQPETEKEFMVEVETIGQTFHKNLVRLLGFCNERAERLLV

JHN19578_LecRK1 YKGQLEDELKTNIAVKTIHKLQPETEKEFMVEVETIGQTFHKNLVRLLGFCNERAERLLV

JHN19874_LecRK1 YKGQLEDELKTNIAVKTIHKLQPETEKEFMVEVETIGQTFHKNLVRLLGFCNERAERLLV

JHN09962_LecRK1 YKGQLEDELKTNIAVKTIHKLQPETEKEFMVEVETIGQTFHKNLVRLLGFCNERAERLLV

RH_LecRK1 YKGQLEDELKTNIAVKTIHKLQPETEKEFMVEVETIGQTFHKNLVRLLGFCNERAERLLV

JHN19671_LecRK1 YKGQLEDELKTNIAVKTIHKLQPETEKEFMVEVETIGQTFHKNLVRLLGFCNERAERLLV

JHNMT2_LecRK1 YKGQLEDELKTNIAVKTIHKLQPETEKEFMVEVETIGQTFHKNLVRLLGFCNERAERLLV

JHNMT1_LecRK1 YKGQLEDELKTNIAVKTIHKLQPETEKEFMVEVETIGQTFHKNLVRLLGFCNERAERLLV

****************.*.********************************** ******

JHN_LecRK1 YEFMTNGPLNRLLFDNSRPHWNTRVHIALGVARGLLYLHDECSKQIIHCDIKPQNILLDD

JHN11183_LecRK1 YEFMTNGPLNRLLFDNSRPHWNTRVHIALGVARGFLYLHDECSKQIIHCDIKPQNILLDD

JHN19525_LecRK1 YEFMTNGPLNRLLFDNSRPHWNTRVHIALGVARGFLYLHDECSKQIIHCDIKPQNILLDD

JHN09962_LecRK1 YEFMTNGPLNRLLFDNSRPHWNTRVHIALGVARGFLYLHDECSKQIIHCDIKPQNILLDD

JHN19578_LecRK1 YEFMTNGPLNRLLFDNSRPHWNTRVHIALGVARGFLYLHDECSKQIIHCDIKPQNILLDD

JHN19874_LecRK1 YEFMTNGPLNRLLFDNSRPHWNTRVHIALGVARGFLYLHDECSKQIIHCDIKPQNILLDD

JHN09962_LecRK1 YEFMTNGPLNRLLFDNSRPHWNTRVHIALGVARGFLYLHDECSKQIIHCDIKPQNILLDD

RH_LecRK1 YEFMTNGPLNRLLFDNSRPHWNTRVHIALGVARGFLYLHDECSKQIIHCDIKPQNILLDD

JHN19671_LecRK1 YEFMTNGPLNRLLFDNSRPHWNTRVHIALGVARGLLYLHDECSKQIIHCDIKPQNILLDD

JHNMT2_LecRK1 YEFMTNGPLNRLLFDNSRPHWNTRVHIALGVARGLLYLHDECSKQIIHCDIKPQNILLDD

JHNMT1_LecRK1 YEFMTNGPLNRLLFDNSRPHWNTRVHIALGVARGLLYLHDECSKQIIHCDIKPQNILLDD

**********************************:*************************

JHN_LecRK1 NLVAKISDFGLAKLLLTNQTRTNTGIRGTRGYVAPEWFKNIGISTKVDVYSFGVILLELV

JHN11183_LecRK1 NLVAKISDFGLAKLLLTNQTRTKTGIRGTRGYVAPEWFKNIGISTKVDVYSFGVILLELV

JHN19525_LecRK1 NLVAKISDFGLAKLLLTNQTRTKTGIRGTRGYVAPEWFKNIGISTKVDVYSFGVILLELV

JHN09962_LecRK1 NLVAKISDFGLAKLLLTNQTRTKTGIRGTRGYVAPEWFKNIGISTKVDVYSFGVILLELV

JHN19578_LecRK1 NLVAKISDFGLAKLLLTNQTRTKTGIRGTRGYVAPEWFKNIGISTKVDVYSFGVILLELV

JHN19874_LecRK NLVAKISDFGLAKLLLTNQTRTKTGIRGTRGYVAPEWFKNIGISTKVDVYSFGVILLELV

JHN09962_LecRK1 NLVAKISDFGLAKLLLTNQTRTKTGIRGTRGYVAPEWFKNIGISTKVDVYSFGVILLELV

RH_LecRK1 NLVAKISDFGLAKLLLTNQTRTKTGIRGTRGYVAPEWFKNIGISTKVDVYSFGVILLELV

JHN19671_LecRK1 NLVAKISDFGLAKLLLTNQTRTKTGIRGTRGYVAPEWFKNIGISTKVDVYSFGVILLELV

JHNMT2_LecRK1 NLVAKISDFGLAKLLLTNQTRTKTGIRGTRGYVAPEWFKNIGISTKVDVYSFGVILLELV

JHNMT1_LecRK1 NLVAKISDFGLAKLLLTNQTRTKTGIRGTRGYVAPEWFKNIGISTKVDVYSFGVILLELV

**********************:*************************************

JHN_LecRK1 CCRRNVELEVVDEEQTIVTYWANDCYRSGRIDLLVEGDDEAIYNIKKVERFVTVALWCLQ

JHN11183_LecRK1 CCRRNVELEVVDEEQTIVTYWANDCYRSGRIDLLVEGDDEAIYNIKKVERFVTVALWCLQ

JHN19525_Lec CCRRNVELEVVDEEQTIVTYWANDCYRSGRIDLLVEGDDEAIYNIKKVERFVTVALWCLQ

JHN09962_LecR CCRRNVELEVVDEEQTIVTYWANDCYRSGRIDLLVEGDDEAIYNIKKVERFVTVALWCLQ

JHN19578_LecRK CCRRNVELEVVDEEQTIVTYWANDCYRSGRIDLLVEGDDEAIYNIKKVERFVTVALWCLQ

JHN19874_LecR CCRRNVELEVVDEEQTIVTYWANDCYRSGRIDLLVEGDDEAIYNIKKVERFVTVALWCLQ

JHN09962_LecRK1 CCRRNVELEVVDEEQTIVTYWANDCYRSGRIDLLVEGDDEAIYNIKKVERFVTVALWCLQ

RH_LecRK1 CCRRNVELEVVDEEQTIVTYWANDCYRSGRIDLLVEGDDEAIYNIKKVERFVTVALWCLQ

JHN19671_LecRK1 CCRRNVELEVVDEEQTIVTD----------------------------------------

JHNMT2_LecRK1 CCRRNVELEVVDEEQTIVTD----------------------------------------

JHNMT1_LecRK1 CCRRNVELEVVDEEQTIVTD----------------------------------------

*******************

JHN_LecRK1 EDPSMRPNMLKVTQMLDGAVAIPSPPDLCSFISSLP

JHN11183_LecRK1 EDPSMRPNMLKVTQMLDGAVAIPSPPDPCSFISSLP

JHN19525_LecRK1 EDPSMRPNMLKVTQMLDGAVAIPSPPDPCSFISSLP

JHN09962_LecRK1 EDPSMRPNMLKVTQMLDGAVAIPSPPDPCSFISSLP

JHN19578_LecRK1 EDPSMRPNMLKVTQMLDGAVAIPSPPDPCSFISSLP

JHN19874_LecRK1 EDPSMRPNMLKVTQMLDGAVAIPSPPDPCSFISSLP

JHN09962_LecRK1 EDPSMRPNMLKVTQMLDGAVAIPSPPDPCSFISSLP

RH_LecRK1 EDPSMRPNMLKVT-----------------------

JHN19671_LecRK1 ------------------------------------

JHNMT2_LecRK1 ------------------------------------

JHNMT1_LecRK1 ------------------------------------

**Data S3** Amino acid sequence analysis of OsLecRK2 gene in JHN, identified mutants and natural BPH resistance varieties, RH. Yellow color was highlighted at amino acid position that found in selected mutant lines.

JHN_LecRK2 MAPILFLPILQILLIYCTKSAQAQLNISIGSSLTPQEVNNSWISPSSDFAFGFRAVDGNS

JHNMT1_LecRK2 MAPILFLPILQILLIYCTKSAQAQLNISIGSSLTPQEVNNSWISPSSDFAFGFRAVDGNS

JHN19525_LecRK2 MAPILFLPILQILLIYCTKSAQAQLNISIGSSLTPQEVNNSWISPSSDFAFGFRAVDGNS

JHN09962_LecRK2 MAPILFLPILQILLIYCTKSAQAQLNISIGSSLTPQEVNNSWISPSSDFAFGFRAVDGNS

JHN12005_LecRK2 MAPILFLPILQILLIYCTKSAQAQLNISIGSSLTPQEVNNSWISPSSDFAFGFRAVDGNS

JHN00004_LecRK2 MAPILFLPILQILLIYCTKSAQAQLNISIGSSLTPQEVNNSWISPSSDFAFGFRAVDGNS

JHN19578_LecRK2 MAPILFLPILQILLIYCTKSAQAQLNISIGSSLTPQEVNNSWISPSSDFAFGFRAVDGNS

JHN19874_LecRK2 MAPILFLPILQILLIYCTKSAQAQLNISIGSSLTPQEVNNSWISPSSDFAFGFRAVDGNS

JHN21689_LecRK2 MAPILFLPILQILLIYCTKSAQAQLNISIGSSLTPQEVNNSWISPSSDFAFGFRAVDGNS

************************************************************

JHN_LecRK2 SSYLLAVWFNKIADKTVIWYAKTSSNGQDDTIPVQVQSGSVLKLADGALSLRDPSGNEVW

JHNMT1_LecRK2 SSYLLAVWFNKIADKTVIWYAKTSSNGQDDTIPVQVQSGSVLKLADGALSLRDPSGNEVW

JHN19525_LecRK2 SSYLLAVWFNKIADKTVIWYAKTSSNGQDDTIPVQVQSGSVLKLADGALSLRDPSGNEVW

JHN09962_LecRK2 SSYLLAVWFNKIADKTVIWYAKTSSNGQDDTIPVQVQSGSVLKLADGALSLRDPSGNEVW

JHN12005_LecRK2 SSYLLAVWFNKIADKTVIWYAKTSSNGQDDTIPVQVQSGSVLKLADGALSLRDPSGNEVW

JHN00004_LecRK2 SSYLLAVWFNKIADKTVIWYAKTSSNGQDDTIPVQVQSGSVLKLADGALSLRDPSGNEVW

JHN19578_LecRK2 SSYLLAVWFNKIADKTVIWYAKTSSNGQDDTIPVQVQSGSVLKLADGALSLRDPSGNEVW

JHN19874_LecRK2 SSYLLAVWFNKIADKTVIWYAKTSSNGQDDTIPVQVQSGSVLKLADGALSLRDPSGNEVW

JHN21689_LecRK2 SSYLLAVWFNKIADKTVIWYAKTSSNGQDDTIPVQVQSGSVLKLADGALSLRDPSGNEVW

************************************************************

JHN_LecRK2 NPRVTDVGYARMLNTGNFRLLGTDGATKWESFGDPSDTILPTQVLPLGTALHSRLLATDY

JHNMT1_LecRK2 NPRVTDVGYARMLNTGNFRLLGTDGATKWESFGDPSDTILPTQVLPLGTALHSRLLATDY

JHN19525_LecRK2 NPRVTDVGYARMLNTGNFRLLGTDGATKWEPFGDPSDTILPTQVLPLGTALHSRLLATDY

JHN09962_LecRK2 NPRVTDVGYARMLNTGNFRLLGTDGATKWEPFGDPSDTILPTQVLPLGTALHSRLLATDY

JHN12005_LecRK2 NPRVTDVGYARMLNTGNFRLLGTDGATKWEPFGDPSDTILPTQVLPLGTALHSRLLATDY

JHN00004_LecRK2 NPRVTDVGYARMLNTGNFRLLGTDGATKWEPFGDPSDTILPTQVLPLGTALHSRLLATDY

JHN19578_LecRK2 NPRVTDVGYARMLNTGNFRLLGTDGATKWEPFGDPSDTILPTQVLPLGTALHSRLLATDY

JHN19874_LecRK2 NPRVTDVGYARMLNTGNFRLLGTDGATKWEPFGDPSDTILPTQVLPLGTALHSRLLATDY

JHN21689_LecRK2 NPRVTDVGYARMLNTGNFRLLGTDGATKWEPFGDPSDTILPTQVLPLGTALHSRLLATDY

****************************** *****************************

JHN_LecRK2 SNGRFQLNVQDDGNLVLYLVAVPSAYYHDPYWASNTVGNGSQLVFNETGRIYFTLTNGSQ

JHNMT1_LecRK2 SNGRFQLNVQDDGNLVLYLVAVPSAYYHDPYWASNTVGNGSQLVFNETGRIYFTLTNGSQ

JHN19525_LecRK2 SNGRFQLNVQDDGNLVLYLVAVPSAYYHDPYWASNTVGNGSQLVFNETGRIYFTLTNGSQ

JHN09962_LecRK2 SNGRFQLNVQDDGNLVLYLVAVPSAYYHDPYWASNTVGNGSQLVFNETGRIYFTLTNGSQ

JHN12005_LecRK2 SNGRFQLNVQDDGNLVLYLVAVPSAYYHDPYWASNTVGNGSQLVFNETGRIYFTLTNGSQ

JHN00004_LecRK2 SNGRFQLNVQDDGNLVLYLVAVPSAYYHDPYWASNTVGNGSQLVFNETGRIYFTLTNGSQ

JHN19578_LecRK2 SNGRFQLNVQDDGNLVLYLVAVPSAYYHDPYWASNTVGNGSQLVFNETGRIYFTLTNGSQ

JHN19874_LecRK2 SNGRFQLNVQDDGNLVLYLVAVPSAYYHDPYWASNTVGNGSQLVFNETGRIYFTLTNGSQ

JHN21689_LecRK2 SNGRFQLNVQDDGNLVLYLVAVPSAYYHDPYWASNTVGNGSQLVFNETGRIYFTLTNGSQ

************************************************************

JHN_LecRK2 INITSAGVDSMGDFFHRATLDTDGVFRQYIYPKSKQARSLWQEQWKAVDALPENICQTIQ

JHNMT1_LecRK2 INITSAGVDSMGDFFHRATLDTDGVFRQYIYPKSKQARSLWQEQWRAVDALPENICQTIQ

JHN19525_LecRK2 INITSAGVDSMGDFFHRATLDTDGVFRQYIYPKSKQARSLWQEQWRAVDALPENICQTIQ

JHN09962_LecRK2 INITSAGVDSMGDFFHRATLDTDGVFRQYIYPKSKQARSLWQEQWRAVDALPENICQTIQ

JHN12005_LecRK2 INITSAGVDSMGDFFHRATLDTDGVFRQYIYPKSKQARSLWQEQWRAVDALPENICQTIQ

JHN00004_LecRK2 INITSAGVDSMGDFFHRATLDTDGVFRQYIYPKSKQARSLWQEQWRAVDALPENICQTIQ

JHN19578_LecRK2 INITSAGVDSMGDFFHRATLDTDGVFRQYIYPKSKQARSLWQEQWRAVDALPENICQTIQ

JHN19874_LecRK2 INITSAGVDSMGDFFHRATLDTDGVFRQYIYPKSKQARSLWQEQWRAVDALPENICQTIQ

JHN21689_LecRK2 INITSAGVDSMGDFFHRATLDTDGVFRQYIYPKSKQARSLWQEQWRAVDALPENICQTIQ

*********************************************:**************

JHN_LecRK2 TKVGSGACGFNSYCTFDGTKNTTNCLCPQRYKFFDNERTYKGCRPDFEPQSCDLDETAAM

JHNMT1_LecRK2 TKVGSGACGFNSYCTFDGTKNTTNCLCPQRYKFFDNERTYKGCRPDFEPQSCDLDETAAM

JHN19525_LecRK2 TKVGSGACGFNSYCTFDGTKNTTNCLCPQRYKFFDNERTYKGCRPDFEPQSCDLDETAAM

JHN09962_LecRK2 TKVGSGACGFNSYCTFDGTKNTTNCLCPQRYKFFDNERTYKGCRPDFEPQSCDLDETAAM

JHN12005_LecRK2 TKVGSGACGFNSYCTFDGTKNTTNCLCPQRYKFFDNERTYKGCRPDFEPQSCDLDETAAM

JHN00004_LecRK2 TKVGSGACGFNSYCTFDGTKNTTNCLCPQRYKFFDNERTYKGCRPDFEPQSCDLDETAAM

JHN19578_LecRK2 TKVGSGACGFNSYCTFDGTKNTTNCLCPQRYKFFDNERTYKGCRPDFEPQSCDLDETAAM

JHN19874_LecRK2 TKVGSGACGFNSYCTFDGTKNTTNCLCPQRYKFFDNERTYKGCRPDFEPQSCDLDETAAM

JHN21689_LecRK2 TKVGSGACGFNSYCTFDGTKNTTNCLCPQRYKFFDNERTYKGCRPDFEPQSCDLDETAAM

************************************************************

JHN_LecRK2 VQYEMTPIDRINWPLSDYEQYSPIDETECRRLCVIDCFCSVAVFNKPSNTCYKKKLPLSN

JHNMT1_LecRK2 VQYEMTPIDRINWPLSDYEQYSPIDETECRRLCVIDCFCSVAVFNKPSNTCYKKKLPLSN

JHN19525_LecRK2 VQYEMTPIDRINWPLSDYEQYSPIDQTECRRLCVIDCFCSVAVFNKASNTCYKKKLPLSN

JHN09962_LecRK2 VQYEMTPIDRINWPLSDYEQYSPIDQTECRRLCVIDCFCSVAVFNKASNTCYKKKLPLSN

JHN12005_LecRK2 VQYEMTPIDRINWPLSDYEQYSPIDQTECRRLCVIDCFCSVAVFNKASNTCYKKKLPLSN

JHN00004_LecRK2 VQYEMTPIDRINWPLSDYEQYSPIDQTECRRLCVIDCFCSVAVFNKPSNTCYKKKLPLSN

JHN19578_LecRK2 VQYEMTPIDRINWPLSDYEQYSPIDQTECRRLCVIDCFCSVAVFNKPSNTCYKKKLPLSN

JHN19874_LecRK2 VQYEMTPIDRINWPLSDYEQYSPIDETECRRLCVIDCFCSVAVFNKPSNTCYKKKLPLSN

JHN21689_LecRK2 VQYEMTPIDRINWPLSDYEQYSPIDETECRRLCVIDCFCSVAVFNKPSNTCYKKKLPLSN

*************************:******************** *************

JHN_LecRK2 GNMDSSLQATVLLKVPRSTNSPSMISSGSSKWKKDKKYWILGSSLFFGSSVLVNFLLIFV

JHNMT1_LecRK2 GNMDSSLQATVLLKVPRSTNSPSMISSGSSKWKKDKKYWILGSSLFFGSSVLVNFLLIFV

JHN19525_LecRK2 GNMNSSLQGTVLIKVPRSTNSPSMISSGSSKWKKDKKYWILGSSLFFGSSVLVNFLLIFV

JHN09962_LecRK2 GNMNSSLQGTVLIKVPRSTNSPSMISSGSSKWKKDKKYWILGSSLFFGSSVLVNFLLIFV

JHN12005_LecRK2 GNMNSSLQGTVLIKVPRSTNSPSMISSGSSKWKKDKKYWILGSSLFFGSSVLVNFLLIFV

JHN00004_LecRK2 GNMNSSLQGTVLIKVPRSTNSPSMISSGSSKWKKDKKYWILGSSLFFGSSVLVNFLLIFV

JHN19578_LecRK2 GNMNSSLQGTVLIKVPRSTNSPSMISSGSSKWKKDKKYWILGSSLFFGSSVLVNFLLIFV

JHN19874_LecRK2 GNMNSSLQGTVLIKVPRSTNSPSMISSGSSKWKKDKKYWILGSSLFFGSSVLVNFLLIFV

JHN21689_LecRK2 GNMNSSLQGTVLIKVPRSTNSPSMISSGSSKWKKDKKYWILGSSLFFGSSVLVNFLLIFV

***:****.***:***********************************************

JHN_LecRK2 LLFGTYCSITSRKKTQLSQLPSNSGLPSKIFTYRELEKATGGFHEVLGTGASGIVYKGQL

JHNMT1_LecRK2 LLFGTYCSITSRKKTQLSQLPSNSGLPSKIFTYRELEKATGGFHEVLGTGASGIVYKGQL

JHN19525_LecRK2 LLFGTYCSITSRKKTQLSQLPSNSGLPSKIFTYRELEKATGGFHEVLGTGASGIVYKGQL

JHN09962_LecRK2 LLFGTYCSITSRKKIQLSQLPSNSGLPSKIFTYRELEKATGGFHEVLGTGASGIVYKGQL

JHN12005_LecRK2 LLFGTYCSITSRKKTQLSQLPSNSGLPSKIFTYRELEKATGGFHEVLGTGASGIVYKGQL

JHN00004_LecRK2 LLFGTYCSITSRKKIQLSQLPSNSGLPSKIFTYRELEKATGGFHEVLGTGASGIVYKGQL

JHN19578_LecRK2 LLFGTYCSITSRKKTQLSQLPNNSGLPSKIFTYRELEKATGGFHEVLGTGASGIVYKGQL

JHN19874_LecRK2 LLFGTYCSITSRKKTQLSQLPNNSGLPSKIFTYRELEKATGGFHEVLGTGASGIVYKGQL

JHN21689_LecRK2 LLFGTYCSITSRKKTQLSQLPNNSGLPSKIFTYRELEKATGGFHEVLGTGASGIVYKGQL

************** ******.**************************************

JHN_LecRK2 QDECGTNIAVKKIEKLQQEAQKEFLVEVQTIGQTFHRNLVRLLGFCNEGTEKLLVYEFMS

JHNMT1_LecRK2 QDECGTNIAVKKIEKLQQEAQKEFLVEVQTIGQTFHRNLVRLLGFCNEGTEKLLVYEFMS

JHN19525_LecRK2 QDECGTNIAVKKIEKLQQEAQKEFLVEVQTIGQTFHRNLVRLLGFCNEGTEKLLVYEFMS

JHN09962_LecRK2 QDECGTNIAVKKIEKLQQEAQKEFLVEVQTIGQTFHRNLVRLLGFCNEGTEKLLVYEFMS

JHN12005_LecRK2 QDECGTNIAVKKIEKLQQEAQKEFLVEVQTIGQTFHRNLVRLLGFCNEGTEKLLVYEFMS

JHN00004_LecRK2 QDECGTNIAVKKIEKLQQEAQKEFLVEVQTIGQTFHRNLVRLLGFCNEGTEKLLVYEFMS

JHN19578_LecRK2 QDECGTNIAVKKIEKLQQEAQKEFLVEVQTIGQTFHRNLVRLLGFCNEGTEKLLVYEFMS

JHN19874_LecRK2 QDECGTNIAVKKIEKLQQEAQKEFLVEVQTIGQTFHRNLVRLLGFCNEGTEKLLVYEFMS

JHN21689_LecRK2 QDECGTNIAVKKIEKLQQEAQKEFLVEVQTIGQTFHRNLVRLLGFCNEGTEKLLVYEFMS

************************************************************

JHN_LecRK2 NGSLNTFLFNDTHPHWSLRVQVALGVSRGLLYLHEECNKQIIHCDMKPQNILLDDNFVAK

JHNMT1_LecRK2 NGSLNTFLFNDTHPHWSLRVQVALGVSRGLLYLHEECNKQIIHCDMKPQNILLDDNFVAK

JHN19525_LecRK2 NGSLNTFLFNDTHPHWSLRVQVALGVSRGLLYLHEECNKQIIHCDMKPQNILLDDNFVAK

JHN09962_LecRK2 NGSLNTFLFNDTHPHWSLRVQVALGVSRGLLYLHEECNKQIIHCDMKPQNILLDDNFVAK

JHN12005_LecRK2 NGSLNTFLFNDTHPHWSLRVQVALGVSRGLLYLHEECNKQIIHCDMKPQNILLDDNFVAK

JHN00004_LecRK2 NGSLNTFLFNDTHPHWSLRVQVALGVSRGLLYLHEECNKQIIHCDMKPQNILLDDNFVAK

JHN19578_LecRK2 NGSLNTFLFNDTHPHWSLRVQVALGVSRGLLYLHEECNKQIIHCDMKPQNILLDDNFVAK

JHN19874_LecRK2 NGSLNTFLFNDTHPHWSLRVQVALGVSRGLLYLHEECNKQIIHCDMKPQNILLDDNFVAK

JHN21689_LecRK2 NGSLNTFLFNDTHPHWSLRVQVALGVSRGLLYLHEECNKQIIHCDMKPQNILLDDNFVAK

************************************************************

JHN_LecRK2 ISDFGLAKLLPVNQTQTNTGIRGTRGYVAPEWFKKIGITSKVDVYSFGVILLELVCCRKN

JHNMT1_LecRK2 ISDFGLAKLLPVNQTQTNTGIRGTRGYVAPEWFKNIGITSKVDVYSFGVILLELVCCRKN

JHN19525_LecRK2 ISDFGLAKLLPVNQTQTNTGIRGTRGYVAPEWFKNIGITSKVDVYSFGVILLELVCCRKN

JHN09962_LecRK2 ISDFGLAKLLPVNQTQTNTGIRGTRGYVAPEWFKNIGITSKVDVYSFGVILLELVCCRKN

JHN12005_LecRK2 ISDFGLAKLLPVNQTQTNTGIRGTRGYVAPEWFKNIGITSKVDVYSFGVILLELVCCRKN

JHN00004_LecRK2 ISDFGLAKLLPVNQTQTNTGIRGTRGYVAPEWFKNIGITSKVDVYSFGVILLELVCCRKN

JHN19578_LecRK2 ISDFGLAKLLPVNQTQTNTGIRGTRGYVAPEWFKNIGITSKVDVYSFGVILLELVCCRKN

JHN19874_LecRK2 ISDFGLAKLLPVNQTQTNTGIRGTRGYVAPEWFKNIGITSKVDVYSFGVILLELVCCRKN

JHN21689_LecRK2 ISDFGLAKLLPVNQTQTNTGIRGTRGYVAPEWFKNIGITSKVDVYSFGVILLELVCCRKN

**********************************:*************************

JHN_LecRK2 VELEVADEEQTILTYWANDCYRCGRIDLLVEGDDEAIFNIKKVERFVAVALWCLQEEPSM

JHNMT1_LecRK2 VELEVADEEQTILTYWANDCYRCGRIDLLVAGDDEAIFNIKKVERFVAVALWCLQEEPSM

JHN19525_LecRK2 VELEVADEEQTILTYWANDCYRCGRIDLLVAGDDEAILNIKKVERFVAVALWCLQEEPSM

JHN09962_LecRK2 VELEVADEEQTILTYWANDCYRCGRIDLLVAGDDEAILNIKKVERFVAVALWCLQEEPSM

JHN12005_LecRK2 VELEVADEEQTILTYWANDCYRCGRIDLLVAGDDEAILNIKKVERFVAVALWCLQEEPSM

JHN00004_LecRK2 VELEVADEEQTILTYWANDCYRCGRIDLLVAGDDEAILNIKKVERFVAVALWCLQEEPSM

JHN19578_LecRK2 VELEVADEEQTILTYWANDCYRCGRIDLLVAGDDEAILNIKKVERFVAVALWCLQEEPSM

JHN19874_LecRK2 VELEVADEEQTILTYWANDCYRCGRIDLLVAGDDEAILNIKKVERFVAVALWCLQEEPSM

JHN21689_LecRK2 VELEVADEEQTILTYWANDCYRCGRIDLLVAGDDEAILNIKKVERFVAVALWCLQEEPSM

****************************** ******:**********************

JHN_LecRK2 RPTMHKVMQMLDGAVQIPTPPDPSSYISSLA

JHNMT1_LecRK2 RPTMHKVMQMLDGAVQIPTPPDPSSYISSLA

JHN19525_LecRK2 RPTMHKVMQMLDGAVQIPTPPDPSSYISSLA

JHN09962_LecRK2 RPTMHKVMQMLDGAVQIPTPPDPSSYISSLA

JHN12005_LecRK2 RPTMHKVMQMLDGAVQIPTPPDPSSYISSLA

JHN00004_LecRK2 RPTMHKVMQMLDGAVQIPTPPDPSSYISSLA

JHN19578_LecRK2 RPTMHKVMQMLDGAVQIPTPPDPSSYISSLA

JHN19874_LecRK2 RPTMHKVMQMLDGAVQIPTPPDPSSYISSLA

JHN21689_LecRK2 RPTMHKVMQMLDGAVQIPTPPDPSSYISSLA

*******************************
